# Supplementary material for: Population genomics of apricots unravels domestication history and adaptive events
Source: Nat Commun. 2021 Jun 25;12:3956. doi: 10.1038/s41467-021-24283-6 (PMC8233370; doi:10.1038/s41467-021-24283-6)
Supplement: Supplementary file 1 — Supplementary Information [file 41467_2021_24283_MOESM1_ESM.pdf]

Population genomics of apricots unravels domestication history and adaptive events

Groppi *et al.*

Supplementary Note 1. Background information on the sequenced accessions and plant material.

Cultivated apricot accessions were kindly provided by: French Centre de Ressources Biologiques of INRAE-GAFL Avignon, Nikita Botanical Garden in Crimea, Ukraine, ARS-USDA National Clonal Germplasm Repository (Davis, California) and Liaoning Pomology Institute and the Nanjing Agricultural University, Faculty of Horticulture, in China. Supplementary Data 1 gives the description of the 578 *Prunus* genomes sequenced in this study. In addition, 348 available *Prunus mume* genome sequences were reanalyzed[1,2]. We labelled as European apricots (EIC) accessions originating from Irano-Caucasia (Iran, Azerbaijan, Armenia, Turkey), Eastern and Western Europe, North Africa (Morocco, Tunisia) and former European colonies (North America, South Africa and New Zealand). In a former study, we showed that they formed a single genetic cluster[3]. Central Asian cultivated apricots were named CA and Chinese cultivars CH (Supplementary Data 1).

Natural populations of *Prunus armeniaca*, *Prunus sibirica* and *Prunus mandshurica* were sampled from 2011 to 2015[3,4], except for *Prunus brigantina*[5]. Details on the sampling sites, GPS coordinates and conditions of sampling are detailed in previous papers[4,3,5]. Wild *P. armeniaca* trees were collected along the Fergana ranges (called S_Par for Southern *P. armeniaca*, Figure 1b) and along the Tian-Shan mountains (called N_Par for Northern *P. armeniaca*, Figure 1b). Putative wild *P. sibirica* were collected from three Western (NW_Psib) and three Eastern (NE_Psib) populations (Supplementary Data 1). No sampling was performed in Southern China because *P. armeniaca* does not grow there and hybrid cultivars between *P. armeniaca* and other *Prunus spp.* did not move far from Chinese center of domestication[6]. The geographic location of all accessions and sampling sites are represented in Figure 1a-b. Among the accessions selected for whole genome *de novo* assembly, Marouch #14 originates from Moroccan oases where it was propagated over many generations by seedlings. The cultivar Stella was first registered in the North American ARS-USDA repository in 1935 (number 11007), provided as budsticks by Mr H. Brayard, Marrakech, Morocco, from, however, an unknown origin. The cultivar Stella's phenotype and phenology are nevertheless similar to wild *P. armeniaca* trees from Central Asia (small leaves, small fruits, late flowering, high chilling requirement). cv. Stella therefore most likely originates from Central Asian or Chinese forests. cv. Stella was chosen here because it serves as a genitor for resistance to pests and pathogens in apricot breeding programs, in particular for its resistance to sharka disease[7]. CH320_5 which served as a representative of *P. sibirica* was collected in the Western Chinese province of

Gansu, at Pingliang, while the Mandchourian apricot (CH264_4) from far-Eastern China was collected along the Khanka Lake, in the Heilongjiang province. The GPS locations of those samples were provided in Liu *et al*[3].

Supplementary Note 2. DNA/RNA preparation and sequencing DNA/RNA extraction and purification.

Genomic DNA was extracted from ~1-2g of fresh, young leaves following the CTAB method[8]. For long-range sequencing, DNA was further purified by equilibrium centrifugation on a cesium chloride gradient. Cesium chloride (1.15 mg / mL) was added to each DNA extraction mixed with 4′,6-diamidino-2-phenylindole dihydrochloride (DAPI) (1 mg/mL) to obtain a relative density of 1.65. This DNA solution was loaded in a Quick-Seal tube (Beckman, ref # 342412) and centrifuged in a VTi 65 vertical-tube rotor at a relative centrifugal field of 220,000 x g at the average radius of the rotor (78.7 mm) for 15 hours. The DNA band was collected from the gradient under ultraviolet light after puncturing the tube with an 18-gauge needle fitted in a plastic syringe. DAPI was extracted with isoamyl alcohol saturated with 10 mM TRIS pH8 and 1 mM EDTA (TE pH8). The cesium chloride was eliminated by dialysis against TE pH8 for two days with changes of dialysis solution on a twice-daily basis, and a final 24 h dialysis against 10mM TRIS pH8. When necessary, the DNA was concentrated by water extraction with butanol-1 and dialysis for 24 h against 10 mM TRIS pH8. Quality controls of the purified DNAs were performed by optic density measurements on a DS-11 DeNovix spectrophotometer and gel electrophoresis to check the DNA size.

Total RNA was extracted from 13 *P. armeniaca* organs and stages of development (Supplementary Data 2) following the method described in Chang *et al*[9]. CH320-5 and CH264.4 total RNAs were extracted from young leaves following the same procedure.

Illumina sequencing

For Illumia sequencing, 1.5 µg of DNA was sonicated to a 100- to 1,500-bp size range using a Covaris E220 sonicator (Covaris, Woburn, MA, USA). Fragments (1 µg) were end-repaired, 3′-adenylated and Illumina adapters were added using the Kapa Hyper Prep Kit

(KapaBiosystems, Wilmington, MA, USA). Ligation products were purified twice with AMPure XP beads (Beckmann Coulter Genomics, Danvers, MA, USA). Libraries were quantified by qPCR using the KAPA Library Quantification Kit for Illumina Libraries (KapaBiosystems), and library profiles were assessed using a DNA High Sensitivity LabChip kit on an Agilent Bioanalyzer (Agilent Technologies, Santa Clara, CA, USA). Four libraries were too low in concentration (qPCR) and were prepared again following this modified protocol: DNA was sonicated to a 100- to 1,500-bp size range using a Covaris E220 sonicator (Covaris, Woburn, MA, USA). After being purified using AMPure XP (Beckmann Coulter Genomics, Danvers, MA, USA), fragments (100 ng) were end-repaired, 3′-adenylated and Illumina adapters (Bioo Scientific, Austin, TX, USA) were added using the NEBNext Sample Reagent Set (New England Biolabs, Ipswich, MA, USA). Ligation products were purified using Ampure XP (Beckmann Coulter Genomics, Danvers, MA, USA) and DNA fragments (>200 bp) were PCR amplified using Illumina adapter-specific primers and the KAPA HiFi HotStart polymerase (KapaBiosystems, Wilmington, MA, USA).

All the libraries were sequenced on an Illumina HiSeq4000 instrument (Illumina, San Diego, CA, USA) using 150 base-length read chemistry in a paired-end mode (minimum coverage: 15x).

Illumina sequencing for genome assembly of CH320-5 and CH264.4

DNA (1.5 µg) was sonicated to a 100- to 1,500-bp size range using a Covaris E220 sonicator (Covaris, Woburn, MA, USA). Fragments (1µg) were end-repaired, 3′-adenylated and Illumina adapters (Bioo Scientific, Austin, TX, USA) were then added using the Kapa Hyper Prep Kit (KapaBiosystems, Wilmington, MA, USA). Ligation products were purified with AMPure XP beads (Beckman Coulter Genomics, Danvers, MA, USA). Libraries were then quantified by qPCR using the KAPA Library Quantification Kit for Illumina Libraries (KapaBiosystems), and library profiles were assessed using a DNA High Sensitivity LabChip kit on an Agilent Bioanalyzer (Agilent Technologies, Santa Clara, CA, USA). Libraries were sequenced on an Illumina HiSeq4000 instrument (Illumina, San Diego, CA, USA) using 150 base-length read chemistry in a paired-end mode.

After the Illumina sequencing, an in-house quality control process was applied to the reads that passed the Illumina quality filters as described in Alberti *et al*[10]. These trimming and removal steps were achieved using Fastxtend tools (http://www.genoscope.cns.fr/externe/fastxtend/). This processing resulted in high-quality data and improvement of the subsequent analyses (Supplementary Data 3).

PacBio long-read sequencing of Marouch #14 and Stella apricots

Library preparation and sequencing were performed according to the PacBio manufacturer's instructions shared protocol-20kb template preparation using BluePippin size selection system (15kb -size Cutoff). At each step, DNA was quantified using the Qubit dsDNA HS Assay Kit (Life Technologies). DNA purity was tested using the nanodrop (Thermofisher) and size distribution and degradation assessed using the fragment analyzer (AATI) high sensitivity large fragment 50kb analysis kit. Purification steps were performed using AMPure PB beads (PacBio). For each library, 10 µg of DNA was purified and then sheared at 40 kb using the megaruptor1 system (Diagenode). Using SMRTBell template Prep Kit 1.0 (PacBio), a DNA end damage repair step was performed on 5µg of the samples. Then blunt hairpin adapters were ligated to the library. The library was treated with an exonuclease cocktail to digest unligated DNA fragments. A size selection step using a 10kb cutoff was performed on the BluePippin size selection system (Sage Science) with 0.75% agarose cassettes, Marker S1 high Pass 15-20kb. Conditioned sequencing primer V2 was annealed to the size-selected SMRTbell. The annealed library was then bound to the P6-C4 polymerase using a ratio of polymerase to SMRTbell at 10:1. For Marouch #14 two SMRTbell libraries were sequenced after a magnetic bead-loading step (OCPW) on 20 SMRT cells on RSII instrument at 0.14nM with a 360 min movie. A total of 19.8 Gb of data was obtained with an estimated coverage of 73X. For Stella, two SMRTbell libraries were sequenced after a magnetic bead-loading step (OCPW) on 16 SMRT cells on RSII instrument at 0.09nM with a 360 min movie. A total of 15.4 Gb of data was obtained with an estimated coverage of 60X.

Preparation of ultra-high molecular weight (uHMW) DNA for Bionano optical mapping

One plant of cv. Stella was put in a dark room for three days before collecting leaf tissues. No dark treatment was applied on the Marouch #14 tree. cv. Stella uHMW DNA was purified from 0.5 gram of very young fresh leaves and Marouch #14 uHMW DNA from one gram of very

young fresh leaves according to the Bionano prep plant tissue DNA isolation base protocol (30068 - Bionano Genomics). Briefly, the leaves were fixed using a solution (Bionano Genomics) containing formaldehyde (Sigma-Aldrich) and then disrupted with a rotor-stator homogenizer (Qiagen) in a homogenization buffer. Nuclei were washed and purified by density gradients and then embedded in agarose plugs. After overnight proteinase K digestion in the presence of lysis buffer (Bionano Genomics) and one-hour treatment with RNAse A (Qiagen), plugs were washed four times in 1x wash buffer (Bionano Genomics) and five times in 1x TE buffer (ThermoFisher Scientific). Then, plugs were melted two minutes at 70°C and solubilized with 2 µL of 0.5 U/µL AGARase enzyme (ThermoFisher Scientific) for 45 minutes at 43°C. A dialysis step was performed in 1x TE Buffer (ThermoFisher Scientific) for 45 minutes to purify DNA from any residue. The DNA samples were quantified by using the Qubit dsDNA BR assay (Invitrogen). The presence of megabase size DNA was visualized by pulsed field gel electrophoresis (PFGE).

Labeling and staining of the uHMW DNA were performed according to the Bionano prep direct label and stain (DLS) protocol (30206 - Bionano Genomics). Briefly, labeling was performed by incubating 750 ng genomic DNA with 1× DLE-1 enzyme (Bionano Genomics) for 2 hours in the presence of 1× DL-Green (Bionano Genomics) and 1× DLE-1 buffer (Bionano Genomics). Following proteinase K (Qiagen) digestion and DL-Green cleanup by membrane adsorption, the DNA backbone was stained by mixing the labeled DNA with DNA Stain solution (Bionano Genomics) in presence of 1× flow buffer (Bionano Genomics) and 0.1M DTT (Bionano Genomics), and incubating overnight at room temperature. The DLS DNA concentration was measured with the Qubit dsDNA HS assay (Invitrogen).

Marouch #14 and cv. Stella optical maps

Labelled and stained DNA for optical maps was loaded on the Saphyr chip. Loading of the chip and running of the Bionano Genomics Saphyr system were all performed according to the Saphyr system user guide (30247 Bionano Genomics). Data processing was performed using the Bionano Genomics Access software (https://bionanogenomics.com/support-page/bionano-access-software/).

For Marouch #14, a total of 354.6 Gb of data was generated. From this data, molecules with a size larger than 150kb, the threshold for map assembly, represented 172 Gb of data. These filtered data (> 150kb), corresponding to 688x coverage of the estimated size of the Marouch #14 genome, were compiled from 578,666 molecules with N50 of 255.8 kb and an average label density of 15.3/100kbp. The filtered molecules were aligned using RefAligner with default parameters. It produced 30 genome maps with a N50 of 14 Mbp for a total genome map length of 244.6 Mbp. A hybrid scaffolding was performed between the PacBio assembly and the optical genome maps using the hybridScaffold pipeline with default parameters. We obtained 19 hybrid scaffolds ranging from 343 kbp to 24.7 Mbp (total length 215.7 Mbp with N50 = 14.6 Mbp) and 103 scaffolds remaining from the PacBio assembly (4.3 Mbp with N50 = 56.1 kbp).

For cv. Stella, a total of 1,249 Gb data was generated. From this data, molecules with a size larger than 150kb represented 322 Gb of data. These filtered data (> 150kb), corresponding to 1,288x coverage of the estimated size of the cv. Stella genome, were compiled from 1,139,316 molecules with N50 of 242.4 kb and an average label density of 18.2/100kbp. The filtered molecules were aligned using RefAligner with default parameters. It produced 53 genome maps with a N50 of 11.3 Mbp for a total genome map length of 292.6 Mbp. A hybrid scaffolding was performed between the PacBio assembly and the optical genome maps with hybridScaffold pipeline with default parameters. We obtained 38 hybrid scaffolds ranging from 308 kbp to 20.8 Mbp (total length 238.2 Mbp with N50 = 14.1 Mbp) and 175 scaffolds remaining from the PacBio assembly (7.2 Mbp with N50 = 54.4 kbp). The optical map step produced assemblies of 215.8 Mb (19 scaffolds) for Marouch #14 and 238.2 Mb (38 scaffolds) for cv. Stella.

CH320_5 and CH264_4 Nanopore sequencing

The libraries were prepared according to the protocols 'Genomic DNA by ligation SQK-LSK108' for MinION and 'Genomic DNA by ligation SQK-LSK109' for PromethION provided by Oxford Nanopore Technologies. Genomic DNA was first repaired and End-prepped with the NEBNext FFPE repair mix (New England Biolabs, Ipswich, MA, USA) and the NEBNext® Ultra™ II end repair/dA-tailing module (NEB). DNA was then purified with AMPure XP beads (Beckman Coulter, Brea, CA, USA) and sequencing adapters (Oxford Nanopore Technologies) were ligated using concentrated T4 DNA Ligase 2M U/mL (NEB).

After purification with AMPure XP beads (Beckman Coulter) using wash buffer (ONT, Oxford Nanopore Technologies) and elution buffer (ONT), the library was mixed with the sequencing bBuffer (ONT) and the library loading bead (ONT), and loaded on the MinION or PromethION flow cells (R9.4.1). Output reads were basecalled using the Albacore basecaller (version 2.3.1 for the MinION runs and version 2.1.10 for the PromethION runs) with default parameters. No data cleaning was performed, the nanopore long reads were used raw for all the assemblies (Supplementary Data 3).

CH320_5 and CH264_4 optical maps

High molecular weight (HMW) DNA was extracted from fresh young leaves using the Bionano prep plant tissue DNA isolation kit, according to the Bionano prep plant tissue DNA isolation base protocol (Bionano Genomics, Inc., San Diego, CA, USA). Briefly, about 1.8 g of young leaves were first fixed in formaldehyde, then ground and homogenized with a tissue ruptor apparatus. Nuclei suspensions were filtered, washed and then purified by Iodixanol density gradient centrifugation. The intact nuclei were embedded in low melting agarose; the plugs were then digested with Proteinase K and RNase A (Qiagen). The purified HMW DNA was obtained by plug lysis with agarase (ThermoFisher) and drop dialysis (Millipore).
The NLRS labeling (BspQI) protocol was performed according to Bionano with 600ng of DNA. The DLS labeling (DLE-1) was performed with 750ng of DNA. Loading of the chip was performed as recommended by Bionano Genomics.

Supplementary Note 3. Genome assembly details.

Assembly of the Marouch #14 and Stella genomes

Genome assembly was performed following the pipeline depicted in Supplementary Figure 1. We used in the first step FALCON v0.7 from PacBio long-reads[11]. Error correction and pre-assembly were carried out with the FALCON-Unzip pipeline after filtering out the reads with a length < 3 kb. The resulting draft assembly was polished using the Quiver pipeline[12], which mapped PacBio reads to the assembled genome with BLASR[13]. Haplotypes were separated during assembly using FALCON-Unzip. The preliminary genome assembly was approximately 209 Mb (302 primary-contigs) for Marouch #14 and 218.3 Mb (391 primary-contigs) for Stella. A summary of assemblies' statistics can be found in the Supplementary Data 4. Assemblies

were then processed with Purge Haplotigs[14] that uses mapped read coverage and Minimap2[15] alignments to determine which contigs to keep for the haploid assembly and the reassigning of allelic contigs.

To further improve these assemblies, we used optical maps to perform hybrid scaffolding and Illumina short read sequences[7] to perform gap-closing with GapCloser 1.12_r6[16]. A last round of polishing was performed with Arrow (https://github.com/PacificBiosciences/GenomicConsensus) that is a consensus model based on a hidden Markov model approach. The metrics of the resultings assemblies were 203.9 Mb (19 scaffolds) for Marouch #14 and 212 Mb (38 scaffolds) for cv. Stella.

Genome assembly of the CH320_5 and CH264_4 genomes

In order to assemble Nanopore long-range reads, we used two different assemblers: SMARTdenovo (git commit 5cc1356)(https://github.com/ruanjue/smartdenovo) and Ra (git commit d388a2f)[17] with all Nanopore reads or subsets of reads. We generated two subsets of reads corresponding to either the longest reads or to reads selected by the Filtlong software (v0.2.0, git commit cf65a48)(https://github.com/rrwick/Filtlong) with default parameters. The following options were given as input to SMARTdenovo: -c 1 to generate a consensus sequence, -J 5000 to remove sequences smaller than 5kb and -k 17 to use 17-mers as advised by the software developers for large genomes. We kept the SMARTdenovo assemblies with all reads (for both *P. mandshurica* and *P .sibirica*) as they had the highest cumulative size and contig N50 (Supplementary Data 3). Both assemblies were then polished three times with the Racon software (v1.3.1, git commit f5af1c7)[17] using Nanopore reads and three times using the Pilon software (v1.23, git commit be801ec)[18] with Illumina reads. Both Racon and Pilon were used with default parameters.

The genome map assemblies of *P. sibirica* and *P. mandshurica* have been generated using the Bionano Solve Pipeline version 3.3 and the Bionano Access version 1.3 (Supplementary Data 3). The assemblies were performed using the following parameters: non-haplotype without extend and split and add Pre-assembly. These parameters enabled a draft assembly as a reference for generating the final assembly. We filtered out molecules smaller than 180 Kb and molecules with less than nine labeling sites (Supplementary Data 3). The size of the resulting

optical maps (with BspQI and DLE-1 enzymes) were nearly two fold higher than expected, due to the level of heterozygosity of both genomes. A high fraction of the genome maps was present in two copies, representing the two alleles. For each genome, we filtered the optical maps and kept a single map for each genomic region. This filtering was made manually by visualizing the mapping of the genome maps against the *P. sibirica* contigs and choosing the maps that show the highest number of aligned labels. Following this process, we obtained filtered genome maps with a size near the expected genome size, and a higher N50 (Supplementary Data 3).

For each genome, the Bionano scaffolding workflow was launched with the Nanopore contigs and the two Bionano filtered maps (Supplementary Data 3). As previously reported[19], we found in several cases that the Nanopore contigs were overlapping (based on the optical map) without being detected by the hybrid scaffolding procedure. We corrected these negative gaps using the BiSCoT software[20] with default parameters (Supplementary Data 3). As recommended, after the BiSCoT output, we performed a last round of polishing using Hapo-G (with default parameters), a polisher dedicated to heterozygous genomes[21].

Allelic duplication removal: The resulting assemblies of the CH264_4 and CH320_5 genomes still contained some allelic duplications due to heterozygous regions which were not collapsed during the assembly. To remove these allelic duplications, we aligned small scaffolds (<1Mb) against the largest ones (>1Mb) using minimap2[15]. Scaffolds with an alignment covering at least 50% of its length were filtered out. BUSCO scores were compared before and after removing allelic duplications, especially the number of duplicated BUSCO genes, that decreased from 57 to 27 for CH264_4 and 51 to 24 for CH320_5[22].

Scaffold ordering using comparative genomics: Scaffolds of CH264_4 and CH320_5 were organized into chromosomes using the Marouch #14 v3.1 reference genome and the RaGoo software version 1.1 (with -b and -C options) (https://github.com/malonge/RagTag)[23].

Supplementary Note 4. Genome quality assessment.

Comparison with previously published *de novo* assembled apricot genomes

Two other *P. armeniaca* genotypes had previously been sequenced using long reads (cv. Chuanzhihong[24]) or a hybrid strategy (cv. Rojo Pasion[25]) (by gamete binning) that allows the separation and assembly of the two haplotypes within diploid genomes. We compared these three existing assemblies with the four that we generated in this study. All the assemblies had large contigs (contig N50 >1Mb), in particular the two haplotypes obtained by gamete binning (contig N50 >25Mb). Not surprisingly, gene content completeness, calculated with BUSCO v4.0.5 dataset[22], is very similar for all seven assemblies (Supplementary Data 5) and the whole genome alignments revealed high synteny, although many rearrangements occurred in centromeres and neighboring regions (Supplementary Figure 2).

Genome heterozygosity

Heterozygosity rate was estimated from the corrected reads by Falcon 0.7 (preads4falcon.fasta). The histogram of k-mer frequencies was computed with jellyfish/2.3.0[26]. This k-mer count distribution was used as input in GenomeScope 2.0 to compute the genome properties and heterozygosity[27]. K-mer spectra are presented in Supplementary Figure 3.

Evaluation of the genome quality with Busco

The quality of the assembled genomes, predicted proteins and assembled transcripts were assessed using the benchmarking universal single-copy orthologs (BUSCO v4.0.5) with the embryophyta_odb10 dataset[22]. BUSCO assessment results are presented in Supplementary Figure 4.

Chromosome anchoring with apricot genetic maps

Scaffolds were aligned to apricot genetic maps to create pseudomolecules covering each chromosome. We used microsatellite (SSR or Simple Sequence Repeat) markers from the cv. Lito x BO81604311[28], cv. Lito x cv. Lito[29], cv. Goldrich x cv. Moniqui[30], cv. Harlayne x cv. Marlén[31] genetic maps. A complete list of the markers tested and primer sequences are available in the Supplementary Data 6. We also benefited from unpublished cv. Bergeron x cv. Bakour map and developed Single Nucleotide Polymorphism (SNP) markers for linkage mapping in the cv. Bergeron x cv. Bakour progenies. SNP markers were developed from previous apricot

ILLUMINA sequences[7]. We designed SSR markers and improved the cv. Goldrich x cv. Moniqui linkage map (Supplementary Data 6). Apricot genetic maps and unpublished SNP/SSR sequences used for genome anchoring are available in the Supplementary Data 6.

Primers from all genetic markers (SNP and SSR) were used to perform an *in silico* PCR with isPCR 33.2[32] on the Marouch #14 and cv. Stella assemblies. *In silico* PCR results were parsed (custom script) to provide input files to ALLMAPS 0.8.4[33] to generate the corresponding anchored maps. These maps were then used by ALLMAPS to order and orient scaffolds into the final chromosome build (Supplementary Figure 5).

Supplementary Note 5. Gene prediction and annotation.

Transcriptome analysis

Illumina paired-end reads were quality filtered with fastp 0.20.0[34]. Mapping against each corresponding newly assembled genome was performed with STAR 2.6.0a[35] by using the ENCODE options adapted to the size of the apricot genome:

```
--outFilterType BySJout \
--outFilterMultimapNmax 20 \
--alignSJoverhangMin 8 \
--alignSJDBoverhangMin 1 \
--outFilterMismatchNmax 999 \
--outFilterMismatchNoverReadLmax 0.04 \
--alignIntronMin 20 \
--alignIntronMax 20000 \
--alignMatesGapMax 20000
```

Resulting bam files were sorted and used as input to perform genome guided RNAseq assembly with Trinity 2.8.4[36,37]. Transdecoder 5.5.0 was used to identify candidate coding regions within transcript sequences from *de novo* Trinity RNA-Seq transcript assembly. The RNAseq data used for transcriptome analysis is detailed in Supplementary Data 2.

Structural annotation

Gene prediction was performed with BRAKER2 starting from the mapped RNAseq and the proteome from a closely related species, *Prunus persica*

(GCF_000346465.2_Prunus_persica_NCBIv2_protein.fa)[38]. The pipeline for gene prediction used BRAKER2[39] and AUGUSTUS[40] (Supplementary Figure 6). Further transcriptome functional annotation and analysis were performed by following the steps described in the pipeline Trinotate[41]. Blast homologies (blastx and blastp)[42] were captured against Uniprot Plant (ftp://ftp.uniprot.org/pub/databases/uniprot/current_release/knowledgebase/taxonomic_divisions/uniprot_sprot_plants.dat.gz) with parameters described by Shah *et al*[43] to produce only the best match result for each query. Hmmscan (HMMER 3.2.1) was run to identify protein domains with E-value < 1e-23 and coverage > 0.2 against PfamA[44,45].

Functional annotation

Protein coding genes were annotated using a pipeline integrating the sources of information described below. Statistics on the predicted proteins are listed in the (Supplementary Data 7). Results were successively integrated depending on the expected accuracy of the source of information. Priority was successively given to: *i)* a BLASTp search of reciprocal best hits with the 244 Rosaceae protein datasets tagged as reviewed in the Uniprot 20200918 database (90% span, 80% identity); *ii)* EC (Enzyme Commission, https://en.wikipedia.org/wiki/Enzyme_Commission_number) numbers assigned to putative enzymes by using E2P2 (version 3.1) tool; *iii)* the transcription factors and kinases identified by ITAK release 1.7; *iv)* transcription factors identified by PlantTFCat; *v)* the Interpro (release 81.0) and BLASTp hits against NCBI NR database 20200824 restricted to *Viridiplantae* proteins as input datasets for Blast2GO annotation service to produce functional descriptions and gene ontology terms. The pipeline and associated tools (except Blast2GO) are available at https://lipm-gitlab.toulouse.inra.fr/LIPM-BIOINFO/nextflow-functionnalannotation/-/tree/1.0.0.

Repetitive elements identification and comparison

Repetitive elements were predicted using the REPET package v2.5 (https://urgi.versailles.inra.fr/Tools/REPET) for Marouch #14, cv. Stella, CH320_5 and CH264_4 genomes that were separately uploaded onto the URGI server (saruman.versailles.inra.fr). The pipeline TEdenovo was run to build the repeat element consensus library. This pipeline followed three main steps: i) align the genomic sequences with themselves to detect high scoring segment pairs (HSPs) that correspond to repeats, ii) build

clusters of the HSPs, and iii) build consensus sequences for each cluster. Each consensus sequence was then characterized using Wicker's classification. The second pipeline, TEannot, was run using the classified consensus library from TEdenovo as input. This step performed successive procedures such as removal of repeat sequence duplicates, removal of simple sequence repeats and comparison of the annotations with data banks. Curation was carried out to refine the repetitive element library by displaying and manually checking the multiple alignments of each consensus. Consensus sequences annotated as potential host genes were removed from the analysis. Only consensus sequences with full length fragments having at least one perfect match in the genome were selected as a validated repeat element library to run another TEannot round. The last round of TEannot provided a repeat element annotation with 100% matched full-length fragment library.

Repeat annotation and comparison between species

Repeat elements represented 37% to 44% of the four high-quality genome assemblies. In *P. armeniaca* (Marouch #14 and cv. Stella), the repeat sequences were predicted to represent 37.47% and 39.93% of the genomes, respectively (Supplementary Data 7 and Supplementary Figure 7). These elements occupy 40.37% of the genome in *P. sibirica* and 44.22% in *P. mandshurica*. The class I elements (retrotransposons) were more abundant (> 20%) than class II elements (> 15%) in all studied genomes (Supplementary Data 7). The most abundant retrotransposons were LTR sequences: 14.34% in Marouch #14, 17.22% in Stella, 18.29% in *P. sibirica* and 19.37% in *P. mandshurica* (Supplementary Figure 8). The non-LTR retrotransposons (LINE and SINE elements) contributed approximately to 3% of these genomes. The unclassified retrotransposons represented almost the same genome percentage (~1 to 2%) as the non-LTR elements (Supplementary Data 7).

Among the DNA transposons, the class II elements, Toll/Interleukin receptor (TIR) superfamily, are the predominant components with 10.4% in CH320_5 and more than 11% in the other three genomes. The unclassified class II elements constituted around 3%. In addition, around 1.6% of the genome in the four *Prunus* species was predicted as repetitive sequences but could not be further classified as retrotransposons or DNA transposons.

Supplementary Note 6. Genomic characterization of the Armeniaca genomes.

Multiple genome alignment and genome comparison

Dot plots of genome alignments two by two were performed by using the minimap2 software package and generated with D-Genies[46] (Supplementary Figure 9).

Localization of putative chromosomal rearrangements and segmental conservation between genotypes and species

The Marouch #14, cv. Stella, CH320_5 and CH264_4 assembled genomes were *in silico* digested with DLE-1 enzyme using fa2cmap_multi_color script provided by Bionano Genomics. The *in silico* optical map assemblies of Stella, CH320_5 and CH264_4 were aligned to the *in silico* optical map of the reference Marouch #14 using the runCharacterize script provided by Bionano Genomics, with the default settings. Alignments were imported into Bionano Access software for visualization and comparison of these genotypes (Supplementary Figure 10). Global alignment is high between the genomes, with no major chromosome rearrangement. The percentage of aligned regions (i.e. the regions that aligned between Marouch #14 and the other genome; in dark blue in Supplementary Figure 10) were 80.7 %, 77.6% and 52.8% of cv. Stella, CH320_5 and CH264_4 genomes, respectively. Thus, the more evolutionarily distant a genome is from Marouch #14 (see Figure 2a), the less alignment is observed. Nevertheless, a few large structural variations such as large inversions were identified (Figure 1d): One on chromosome 2 between Marouch #14 and cv. Stella and one on the upper part of chromosome 4 between Marouch #14 and all three other genomes (Supplementary Figure 10).

Analysis of structural variants

The assembly alignments obtained above were used to perform structural variant calling using the runSV script provided by Bionano Genomics, with default settings. The smap file resulting from this analysis was imported into Bionano Access software for visualization of the structural variations. This smap file was sorted to extract the insertions, deletions, inversions, duplications and translocations. Briefly, the insertions and deletions with confidence equal to -1 were filtered out. The two-line inversion entries were flatten into single-line entries. The duplications were

all checked manually by visualization on the Access software. For chromosome 4, we have also manually checked the concordance of this structural variation calling with the alignment of the sequence with minimap and by dot plot with D-genies. In total, 63% of the structural variations detected with the optical maps could be validated by this visualization. The other structural variations being too small to be visualized with D-genies were validated by specific alignment of the sequences with the clustalw software. A verification of the ca. 600Kb inversion was further performed by polymerase chain reaction targeting the right and left borders of the inverted fragment (Supplementary Figure 10c). It should be noted that the number of duplications and translocations detected by the algorithm is probably not exhaustive. The R package OmicCircos was used to edit the circos plot figures from the sorted smap file[47].

Structural variation analysis between Marouch #14 and the other three genomes, cv. Stella, CH320_5 and CH264_4, identified a large number of structural variations (Supplementary Data 8). CH320_5 exhibited the most SVs with 61.6 Mb of SVs, cv. Stella had a total length of 54.4 Mb of structural variations (SV) and CH264_4 had a total length of 14.6 Mb of SVs.

When compared with the Marouch #14 genome organisation, the total number of SVs (Supplementary Data 8 and 9), and the SV distribution per chromosome (Supplementary Figure 11) were similar for cv. Stella and CH320_5 while we detected less variants for *P. mandshurica* CH264_4 (Figure 1d). These results suggest a closer genetic relationship between Marouch #14, cv. Stella and CH320_5 than between Marouch #14 and CH264_4. Since the *P. mandshurica* CH264_4 genome showed less alignment with Marouch #14 (52.8%) than cv. Stella or CH320_5 (80.7 and 77.6%, respectively), we suspect that the lack of alignment limits the detection of structural variants. A majority of variants was detected over chromosome 1 (Supplementary Figure 11) but this chromosome is also the biggest among the *Prunus* chromosomes. SV distribution along the chromosomes was not related to gene or TE density (Figure 1d). In our analysis, structural variants ranged from 501 bp to 4.1 Mb. The majority of the structural variants detected were small-sized variants with a median structural variant size of 5.9 kb (Supplementary Figure 12).

Supplementary Note 7. Phylogenetic analyses of nuclear genomes.

Armeniaca nuclear phylogenetic tree

Using Orthofinder 2.3.11, we identified 298 single-copy orthologous sequences shared among the 12 following species: *Arabidopsis thaliana*, *Populus trichocarpa*, *Vitis vinifera*, *Rosa chinensis*, *Fragaria vesca*, *Prunus persica* cv. Lovell, *P. dulcis* cv. Texas, *P. mume, P. mandshurica* CH264_4, *P. sibirica* CH320_5, *P. armeniaca* Marouch #14 and *P. armeniaca* cv. Stella (Supplementary Data 10)[48,49]. Information on the proteome fasta files used in this study is depicted in Supplementary Data 10.

To estimate the divergence times among Armeniaca species, we used these 298 single-copy orthologous genes shared by all twelve species. Four-fold degenerate sites from these single-copy orthologous genes were extracted and concatenated for each species using in-house perl scripts. Sequence alignment performed with mafft 7.453 [50] was used to construct a phylogenetic tree with PhyML-aLRT 3.3[51,52] (Supplementary Figure 13).

We used BEAST 2.6.2[53] that implements Bayesian Markov chain Monte Carlo (MCMC) method to infer phylogenetic relationships of the 12 species. The BEAST xml file was constructed by using BEAUTi[54]. We used the calibrated Yule model[55] by setting the divergence time between *Fragaria vesca* and *Rosa chinensis* around 34.1 MYA[56] and the divergence between *Arabidopsis thaliana* and *Populus trichocarpa* around 110 MYA[57]. We also added a prior for the divergence dates among the four Armeniaca species *P. mandshurica* CH264_4, *P. sibirica* CH320_5, *P. armeniaca* Marouch #14 and *P. armeniaca* cv. Stella.

The length of chain, i.e. the number of steps that the MCMC will make in the chain before finishing, was set to 50,000,000. BEAST was run twice with the same parameters to check result convergence. Tracer 1.7.1[58] was used to analyze the output of the two BEAST runs. Effective sample size (ESS) of a parameter sampled from a MCMC (BEAST) is the number of effectively independent draws from the posterior distribution that the Markov chain is equivalent to. ESS values of our parameters were all high (over 200 or so), indicating that both runs had converged to the same stationary distribution. Thus, we were confident that our runs had sampled the same distribution (Figure 2a). FigTree 1.4.4 (https://github.com/rambaut/figtree) was used to display summarized and annotated trees produced by BEAST (Figure 2a).

Armeniaca and Rosaceae genome arrangement history reconstruction

The evolutionary scenario of genome arrangement was inferred following the method described in Pont *et al*[59] based on synteny relationships identified between the genomes of *P. sibirica* CH320_5, *P. armeniaca* (cv. Stella, Marouch #14), *P. mandshurica* CH264_4 delivered in the current article as well as those with the genomes of rosa (7 chromosomes, 49,767 genes)[60], Japanese apricot *P. mume* (8 chromosomes, 31,390 genes)[1], almond cv. Texas (8 chromosomes, 27,969 genes)[61,62], peach (8 chromosomes, 27,864 genes)[38], apple (17 chromosomes, 63,514 genes)[63], pear *Pyrus bretschneideri* (17 chromosomes, 42,812 genes)[64] and woodland strawberry (7 chromosomes, 32,831 genes)[65]. Briefly, the first step consisted in aligning the genomes to identify conserved/duplicated gene pairs on the basis of alignment parameters (CIP for cumulative identity percentage and CALP cumulative alignment length percentage). The second step consisted in clustering or chaining groups of conserved genes into synteny blocks (excluding blocks with less than 5 genes) corresponding to independent sets of blocks sharing orthologous relationships in modern species. In the third step, conserved gene pairs or conserved groups of gene-to-gene adjacencies defining identical chromosome-to-chromosome relationships between all the extant genomes were merged into conserved ancestral regions that were then merged into protochromosomes based on partial synteny observed between a subset (not all) of the investigated species. The ancestral karyotype can be considered as a 'median' or 'intermediate' genome consisting in protochromosomes defining sets of genes with shared order among the extant species. We identified an ancestral Rosaceae karyotype consisting of nine protochromosomes with 8,848 genes by comparing the representative genomes of *Maloideae*, *Prunoideae* and *Rosoideae* (i.e., apple, rose, peach and strawberry, respectively). We then compared the ancestral Rosaceae karyotype, following the same approach, with the Armeniaca genomes (*P. sibirica* CH320_5, *P. armeniaca* Marouch #14 and cv. Stella, *P. mandshurica* CH264_4, *P. mume*), rose and pear (*Pyrus bretschneideri*). From the reconstructed ancestral karyotype, an evolutionary scenario was inferred as the one assuming the fewest number of genomic rearrangements (i.e., inversions, deletions, fusions, fissions, translocations) required between the inferred ancestors and the modern genomes.

In order to assess the genomic rearrangement history of apricots within the Rosaceae family, we performed a comparative genomic investigation of Armeniaca genomes (*P. sibirica*, *P.*

armeniaca cv. Stella, Marouch #14, *P. mandshurica*, *P. mume*) together with almond (*P dulcis*), rose *Rosa chinensis*, peach (*P. persica*), apple (*Malus domestica*), pear (*Pyrus bretschneideri*) and strawberry (*Fragaria vesca*), using grape[66] as an outgroup and the genome alignment parameters and ancestral genome reconstruction methods described in Pont *et al*[59]. Conserved genes adjacencies identified conserved ancestral regions defining an ancestral Rosaceae karyotype with nine protochromosomes (Figure 2b). The complete synteny-based deconvolution into nine reconstructed conserved ancestral regions of the observed synteny between ancestral Rosaceae karyotype and the investigated species validated the nine proposed protochromosomes as the origin of Rosaceae (Supplementary Figure 14). Our evolutionary scenario, comparing the modern genome structures of Rosaceae genomes, established that ancestral Rosaceae karyotype evolved from the ancestral Eudicot karyotype with seven protochomosomes that experienced a whole-genome triplication (γ) to reach a 21-chromosomes karyotype[67]. From the ancestral Rosaceae karyotype, specific chromosome shuffling events shaped the *Rosoideae* ancestor (leading the modern strawberry and rose genomes) structured into eight protochromosomes, through an ancestral chromosome fission and two fusions. The duplication of the ancestral Rosaceae karyotype, followed by at least 11 chromosome fissions and 12 fusions shaped the *Maloideae* ancestor (leading to the modern apple and pear genomes) with 17 protochromosomes. The seven modern *Prunus* genomes investigated derived from the reconstructed *prunoideae* ancestor with eight protochromosomes, deriving from the ancestral Rosaceae karyotype through two ancestral chromosome fissions and four fusions. Synteny relationships between the five apricot genomes established that *P. sibirica* displayed the karyotypic structure closest to the ancestral Rosaceae karyotype (with 7,451 genes conserved), followed by the two *P. armeniaca* (cv. Stella and Marouch #14) genomes (with 7,380 and 7,389 genes conserved respectively), *P. mume* (with 7,219 protogenes conserved) and *P. mandshurica* (with 7,159 ARK genes conserved) (Figure 2c). Our comparative genomics-based evolutionary scenario approach unraveled the Rosaceae genome rearrangement history from the reconstructed ancestral Rosaceae karyotype and delivered a complete catalog of shared orthologs (8,848 genes, Supplementary Data 10c) between apricot genomes, that can now be used as a guide to perform translational research between the investigated species to accelerate the dissection of conserved agronomic traits. For that purpose the comparative genomics data described here are made available in the public web tool https://urgi.versailles.inra.fr/synteny/rosaceae, the list of shared orthologs in Rosaceae is depicted in Supplementary Data 10c. The Supplementary Data 11 illustrates the synteny

between peach and the Armeniaca genomes: *Prunus mandshurica* CH264_4, *Prunus sibirica* CH320_5, *Prunus armeniaca* Marouch #14 and cv. Stella, *Prunus mume*.

Supplementary Note 8. Chloroplast genome phylogeny and haplotype composition of the Armeniaca section.

Reconstruction of chloroplast genomes

Short-insert (Illumina) reads for 578 *Prunus* accessions (Supplementary Data 1), together with 15 additional *P. mume* data sets (SRR5046698, SRR5046700, SRR5046714, SRR5046715, SRR5046716, SRR5046727, SRR5046729, SRR5046743, SRR5046744, SRR5046746, SRR5046747, SRR5046748, SRR5046749, SRR5046750, SRR5052874; NCBI BioProject PRJNA352648) were used for reference-based reconstruction of chloroplast genomes. First, chloroplast-matching reads were extracted from fastq data sets using the filter_by_blast script from seq_crumbs (https://github.com/JoseBlanca/seq_crumbs). The filtering used a custom blast database containing a *P. mume* cpDNA (NC_023798) and our own *de novo* assembly of Marouch #14 cpDNA, and was performed in a paired-read mode with $1e^{-08}$ E-value threshold. For each accession, the extracted reads were subsequently de-duplicated retaining the quality scores, and quality-trimmed with Trimmomatic-0.38[68], using the ILLUMINACLIP function for adapter removal. The resulting reads were then individually mapped onto the Marouch #14 cpDNA reference in Geneious 6.1 (https://www.geneious.com), using only paired reads mapping within expected distance, word length 16, allowing up to 2% mismatches and 4% gaps. Individual consensus sequences resulting from the assembly were then aligned to the Marouch #14 cpDNA reference, including a cpDNA assembly available for *P. padus* (KP760072). The second inverted repeat region was deleted from the alignment. The entire alignment was visually inspected, and ambiguously aligned regions were either manually corrected or removed.

Chloroplast genealogy

For phylogenetic inference, we selected 2-4 reconstructed chloroplast genomes per species, representing the cpDNA diversity of wild and cultivated *P. armeniaca, P. sibirica, P. mume* and *P. brigantina.* The cpDNA assembly of Chinese cherry *P. padus* (KP760072) was included as an outgroup. Monomorphic sites and sites with >50% gaps were removed from the

alignment, yielding 2,132 variable sites. A maximum likelihood tree was constructed with IQ-TREE 1.6[69], using ascertainment bias correction, nonparametric bootstrap (1,000 replicates) and Shimodaira–Hasegawa likelihood ratio test SH-aLRT (1,000 replicates). Supplementary Figure 15 shows a maximum likelihood tree computed with the TVMe+ASC+R2 iqtree evolutionary model chosen by ModelFinder as the best-fitting model with support values (SH-aLRT / bootstrap).

The tree reconstructed from the whole chloroplast genomes confirmed, with maximum statistical support, most of the known relationships within the genus *Prunus* (Supplementary Figure 15). Some of the *P. sibirica* chloroplast genomes were differentiated from wild and cultivated apricots and were resolved, as expected, as a sister group to *P. brigantina* with maximum support (100%); however, other *P. sibirica* chloroplast genomes were indistinguishable from those found in *P. armeniaca* (Supplementary Figure 15). Hence, *P. sibirica* harbors two diverged chloroplast lineages that do not form a clade, possibly indicating that some populations are either: misclassified, a product of *P. armeniaca* x *P. sibirica* hybridization, or there has been cpDNA introgression from *P. armeniaca* into *P. sibirica*.

We further computed a haplotype network from the full dataset of the reconstructed chloroplast genomes. For this purpose, we excluded *P. padus* and *P. persica* and used *P. dulcis* (almond) as an outgroup and extracted all polymorphic sites from coding sequences and rRNA genes, yielding an alignment of 574 positions. A median-joining network constructed with Network5[70] summarizes the evolutionary relationships between the identified haplotypes, as well as their prevalence (Figure 3). The four groups of cultivated *P. armeniaca,* differentiated by colour in the network, are based on the geography of sampling sites. Node sizes are proportional to the number of accessions with given haplotypes and the numbers near edges indicate the number of mutations separating two haplotypes.

The network mirrors the pattern observed on the maximum likelihood tree and confirms that a substantial part of *P. sibirica* haplotypes are identical to those found in *P. armeniaca*. The corresponding *P. sibirica* individuals (including the CH320-5 assembled genome) were sampled from the Western natural populations from Gansu and Shaanxi provinces in China (Supplementary Data 1 and Figure 1). Three closely related haplotypes were found in most *P. armeniaca* individuals (A1, A2, A3), which were also found in those Western *P. sibirica* samples. As in the maximum likelihood tree, *P. mume* (haplogroup B) was resolved as a sister group to this *P. armeniaca*-*P. sibirica* cluster (haplogroup A), while the distinct *P. sibirica* haplotypes (haplogroup D) and the *P. brigantina* haplogroup C were found to have diverged

earlier. Regarding cultivated apricots, Central Asian and Chinese cultivars were composed of the three major haplotypes (A1, A2 and A3). In contrast, all European/Irano-Caucasian cultivars carried either the haplotypes A1 or A2, without a single occurrence of the haplotype A3, which was 8-9 mutations distant from the former two.

Supplementary Note 9. Population genomics of Armeniaca.

Population genome sequencing

We sequenced the genomes of 558 Armeniaca accessions comprising 165 cultivated European, 322 Central Asian (64 cultivated and 258 wild) and 71 Chinese (27 cultivated and 44 wild trees assigned to the *P. sibirica* *N*=43 and *P. mandshurica* (*N*=1) species) (Supplementary Data 1). We also sequenced the genomes of four *P. mume* landraces, 14 wild *P. brigantina*, one peach (cv. Honey Blaze) and one almond (cv. Del Cid). Over 2,510 Gb of high-quality cleaned sequence data were generated, with an average of 4.3 Gb and $3.4*10^7$ reads per accession (equivalent to approximately 21x coverage) (Supplementary Data 1). We also downloaded the *P. mume* raw data from 333 landraces and 15 wild genomes[2]. A total of 926 Armeniaca and Armeniaca related genomes were used in the current study and processed following the pipeline summarized in Supplementary Figure 16).

SNP calling

For SNP (Single Nucleotide Polymorphism) calling, the non-Armeniaca samples were removed (peach cv. HoneyBlaze, almond cv. Del Cid) as well as the single *P. mandshurica* representative (CH_264_4, Supplementary Data 1) and the two plum x *P. armeniaca* interspecific hybrids detected in the cp haplotype network (i.e., A3865 and US196) (Figure 3). The number of accessions used for population genomics analyses were as follows: 348 *P. mume* and 555 apricot accessions including cultivated (*N*=254) and wild (*N*=211) *P. armeniaca*, 43 *P. sibirica* from the Western (*N*=21) and Eastern (*N*=22) Chinese Armeniaca-related species sampled in natural populations. In total, we ended up with genomes of 903 accessions, whose sampling sites are depicted in Figure 1.

Illumina raw reads were aligned to the European *P. armeniaca* genome Marouch #14, with an average mapping rate of 70% after elimination of poor sequencing quality reads and PCR duplicates (Supplementary Data 1). Paired raw sequencing reads were trimmed using cutadapt (version 1.2.1)[71] to remove the adapter sequences, and filtered using the NGS QC-toolkit (version 2.3.3)[72] to remove bases with average quality scores below 20. Trimmed reads were mapped to the diploid *Prunus armeniaca* Marouch #14 reference genome with modified parameters implemented in BWA (version 0.7.17)[73] using the BWA-MEM algorithm. To account for the occurrence of PCR duplicates introduced during library construction, we used MarkDuplicates in picard-tools version 2.9.2 (Picard Toolkit. 2019. Broad Institute, GitHub Repository. http://broadinstitute.github.io/picard/) to remove reads with identical external coordinates and insert lengths. We used HaplotypeCaller and GenotypeGVCFs implemented in GATK (version 3.8)[74] to obtain a set of preliminary SNPs and to call genotypes for all samples. Due to the absence of reference data for the GATK analysis steps of indel realignment" and base quality score recalibration (BQSR), we used a self-training strategy of performing three rounds of variant calling by GATK. Briefly, we implemented an initial round of SNP calling on the original uncalibrated data. Then we used the variant filtration function with default parameters in GATK to filter the SNPs. The SNPs with the highest confidence are then used as the database of known SNPs by feeding it as a variant call format (VCF) file to the base quality score recalibration. This step is repeated in the second round calling analysis. We validated the final round of SNP calling with the recalibrated data. After three rounds of calling variants, a final VCF file without obvious difference was obtained. The genomic variants, in GVCF format for each accession, were identified with the HaplotypeCaller module and the GVCF model. All GVCF files were merged and a raw population genotype file with the SNPs and indels was created in the HaplotypeCaller module and was filtered with the following parameters:--filterExpression function QD < 2.0 || FS > 60.0 || MQ < 40.0 || MQRankSum < -12.5 || ReadPosRankSum < -8.0 for snps and QD < 2.0 || FS > 200.0 || ReadPosRankSum < -20.0 for indels.

A subset of 15,111,266 bi-allelic SNPs (SNP quality > 30 and missing data < 15%) in the 903 Armeniaca accessions from the entire SNP dataset was identified, reaching on av. 74 SNPs per Kb.

Linkage disequilibrium analysis

First, we estimated linkage disequilibrium separately for the 348 *P. mume* samples and the 555 genomes from other Armeniaca species (Supplementary Figure 17). 50,000 SNPs were randomly selected from each chromosome. We quantified LD using the squared correlation coefficient (r^2) between pairs of SNPs over a 300 Kb physical distance as implemented in PLINK v1.9[75]. The decayed physical distance between SNPs was identified as the distance at which the maximum r^2 dropped by half (averaged in short range of 10 bp)[76] (www.cog-genomics.org/plink/1.9/). LD decay did not show significant differences among the eight chromosomes, so that all chromosomes were pooled. *Prunus mume* showed a higher LD than the rest of the Armeniaca species (Supplementary Figure 17 A). We observed that the slope of all curves flattened within 1 Kb. A previous study found a similar range of LD decay in a set of cultivated European and North-American apricots[7] .

Second, we estimated LD separately for the apricot groups detected with *fast*STRUCTURE *i.e.*, cultivated European (EIC), Chinese (CH), wild Central Asian *P. armeniaca* (S_Par and N_Par) and wild Western-Chinese populations (NW_Psib). The European cultivars displayed the highest LD level (grey curve in Supplementary Figure 17 B), whereas the Chinese cultivars and the apricot wild relatives displayed the lowest LD (Supplementary Figure 17B). The average distance over which LD decayed to ~50% of its maximum value was lower for the Central Asian wild group (1,215 and 405 bp for the Southern and Northern populations, respectively) than for the European cultivated group (8,295 bp) (Supplementary Figure 17).

Supplementary Note 10. Extensive gene flow between Armeniaca genetic clusters.

Several statistics (e.g., per-individual missingness and identity-by-descent in PLINK v1.9)[75] indicated that our variant-calling pipeline did not perform optimally on species that were phylogenetically distant from the Marouch #14 reference genome, leading to high proportions of missing data and underestimation of inter-specific genetic differentiation and structure. This appears to be a general problem of haplotype callers[77]. For this reason, we removed all 37 accessions with per-individual missingness >25%, including *P. dulcis, P. davidiana, P. salicina, P. persica* and all *P. brigantina* trees. We removed all indels, sites with outlier mean depth (<0.425x and >22.7x), sites with heterozygosity excess >3, sites with minor allele count <4, and sites with per-site missingness >2%, yielding a nucleotide diversity matrix with 6,944,899 SNPs out of the 15,111,266 initially called SNPs. We investigated the occurrence of

gene flow among populations using the ABBA-BABA test implemented in *D*-suite[78,79]. This approach infers ancestral ('A') and derived ('B') alleles across the genomes of quartet of populations; when two derived alleles are present, one expects to find them in the most closely related taxa, *i.e.*, to be found as AABB or BBAA. Without introgression, two particular allelic patterns 'ABBA' and 'BABA' should occur equally frequently, *e.g.*, due to incomplete lineage sorting. An excess of either ABBA or BABA, resulting in a *D* statistic that is significantly different from zero, is indicative of gene flow between two taxa. *D*-suite does not assume a population tree *a priori*, but infers it from the data for each possible quartet of populations with a fixed outgroup. Populations are then assigned to P1, P2 and P3 according to a tree topology (((P1,P2),P3), outgroup) in such a way that P1 and P2 share the highest number of derived variants (the BBAA count), and P3 shares more derived alleles with P2 than it does with P1 (*i.e*. ABBA>BABA). Finally, introgression/gene flow between P2 and P3 is quantified using the *D* statistics.

For the ABBA-BABA analysis, we fixed the North-Eastern *P. sibirica* (NE_Psib) as the outgroup and split the dataset into 100 jackknife blocks. Very high proportions of shared derived variants (BBAA) were found between the cultivated European and Central Asian apricots (EIC and CA), and between cultivated and wild Central Asian *P. armeniaca* (Supplementary Data 12). Note that the Central Asian natural populations (S_Par and N_Par, Figure 1a) were not distinguishable in this analysis. However, in the quartet where all three of these populations were analysed together, the Central Asian cultivated apricots and the wild *P. armeniaca* were sister populations, and highly significant footprints of gene flow was detected between European and Central Asian cultivated apricots (EIC and CA).

The situation was less clear in the case of the Chinese populations, Chinese cultivated apricots and North_Western Chinese *P. sibirica* (CH and NW_Psib in Figure 1a). While principal component analysis (PCA, Figure 4a) indicated that these populations were closely related, *D*-suite resolved them as sister populations (Supplementary Data 12). All quartets featuring CH identified this population as early-diverging (P3), and often involved in gene flow (with wild and cultivated Central Asian *P. armeniaca*). In all quartets with Chinese cultivated apricots (CH) and North_Western *P. sibirica* (NW_Psib), these two populations were never resolved as the most recently diverged, and North_Western *P. sibirica* shared more derived variants with

European and Central Asian cultivated and with Central Asian wild *P. armeniaca*. This suggests that Chinese cultivated apricots and North_Western *P. sibirica* may not be sister populations, and that North_Western *P. sibirica* diverged more recently, most probably having a feral origin. Moreover, the proportions of shared derived variants suggested that North_Western *P. sibirica* was most closely related to wild and cultivated Central Asian *P. armeniaca*. Gene flow with North_Eastern *P. sibirica*, the genuine *P. sibirica* populations, was not examined by this test as *D*-suite does not test gene flow with outgroups.

Supplementary Note 11. Genetic relatedness and structure among Armeniaca populations and apricot accessions.

We used population genetics methods to (i) determine the genetic structure in the wild and cultivated Armeniaca species and then (ii) infer the evolutionary history of these identified wild and cultivated Armeniaca genetic groups using approximate Bayesian computation (Supplementary Note 12).

Genetic subdivision among the Armeniaca species

To get rid of any potential bias for demographic inference using SNPs under selection, we filtered SNPs with minor allele frequency (MAF) <0.05, and kept only bi-allelic SNPs resulting in a dataset of 95,686 SNPs. We pruned SNPs using the mean LD decay r^2 value (0.0428) of *P. armeniaca*. To that aim, we used PLINK v1.90[75] with the following options: 50bp, 50 SNP windows, move every 5 SNPs. From this set of 95,686 SNPs, we ran Bayesian clustering analysis (fastStructure[80]) on three distinct datasets: (1) the whole Armeniaca dataset made of 917 individuals (after removal of the other *Prunus* species, outside of the Armeniaca section, and of interspecific hybrids) (Figure 4d) (Supplementary Figure 18) , (2) *P. mume* (*N*=348) (Supplementary Figure 19) and (3) the rest of the Armeniaca species that were sequenced in the current study (later referred as the ‘other Armeniaca’, *N*=555) (Supplementary Figure 20). fastStructure analysis was performed ten times on the filtered 95,686 SNPs dataset. The marginal likelihood of the *K* values was the highest for *K*=12 for the whole Armeniaca dataset (Supplementary Figure 21 A), for *K*=8 for the *P. mume* accessions (Supplementary Figure 21 B), similar to previous publication[2] and for *K*=7 for the other Armeniaca accessions (Supplementary Figure 21 C).

We considered the seven and eight clusters as the most relevant subdivisions for *P. mume* and other Armeniaca, respectively (Supplementary Figure 19 and Supplementary Figure 20). We considered hereafter a genotype to be unequivocally assigned to a population when its assignment probability was ≥ 90% to one of the above clusters, which was the case for 41% (143 out of 348) of the *P. mume* individuals and 62% (345 out of 555) of other Armeniaca species (Supplementary Figure 22 a and b). *Prunus mume* was highly admixed, as described previously[2] while the European Irano-Caucasian (EIC) samples split into four subgroups: EIC_A to EIC_D (Supplementary Figure 20). The four genetic groups corresponded to the four geographical groups identified in Bourguiba *et al*[81]: Mediterranean and Continental Europe, North-American and North-African. However, the North-American group and European modern breeding accessions (EIC_C) were entirely admixed (i.e., with a membership coefficient < 90% to any cluster, e.g., the membership coefficient was 83% for the Stark Early Orange cultivar ('SEO'). The North-American and European modern breeding accessions were therefore not retained for further analyses. fastSTRUCTURE revealed that the Chinese cultivars, the North Western *P. sibirica*, and the Central Asian (CA) cultivars were also highly admixed. For example, in the Central Asian cultivated apricots, only four accessions had membership coefficient ≥ 90% to a given genetic cluster, and all four are related to EIC_D with *PI_hat* > 0.5 by IBD analysis (see below). fastSTRUCTURE further supported the high level of wild-to-crop gene flow in Central Asia. Genome-wide admixture revealed with fastSTRUCTURE was consistent with the hypothesis of wild-to-crop gene flow: varying K from 2 to 9, we consistently observed wild ancestry in the cultivated apricot groups, both in the Central Asian and Chinese cultivated accessions (Supplementary Figure 20; Supplementary Figure 23). For K=5, 100% of the Central Asian cultivated accessions showed a membership coefficient >10% into the Southern and/or Northern wild *P. armeniaca* gene pools (Supplementary Figure 23b). Gene flow from the North Eastern *P. sibirica* was detected only in the Chinese cultivated apricots (Supplementary Figure 23c).

Pedigree construction and clonal relationships

For the second round of subdivision analysis, we retained cultivated and wild Armeniaca accessions that displayed an assignment probability ≥ 90%. From this set of individuals, we used PLINK v1.90[75] to estimate IBS (*Identity-By-State*) for each pair of individuals based on

the average proportion of alleles shared at genotyped SNPs among the Armeniaca accessions. The degree of recent shared ancestry, i.e. the identity by descent (IBD) can be estimated from the genome-wide IBS. We considered a pair of accessions to be genetically identical (i.e., clonemates) if they had an IBD P(Z2) or P(IBD=2) >95% (empirical cut off value for excluding clones in grape[82]). The IBD P(Z2) values for a large majority of pairs of accessions were close to zero (Supplementary Figure 24 A and B). Five *P. mume* accessions of the G1 cluster displayed an IBD P(Z2) value >0.95 (SRR5046580, SRR5046581, SRR5046582, SRR5046626, SRR5046664) of which we retained one single clone, SRR5046580. To identify parent-offspring and other close pedigree relationships, we used the Z1 and Z2 values observed from our confirmed parent-offspring pairs, through the calculation of the *PI-hat* score computed by PLINK v1.90[75] (Supplementary Figure 24 C and D). Indeed, the proportion of IBD (*Identity-by-Descent*) between two individuals is returned by a *PI_hat* score that assesses the relatedness among two samples such as P(Z2) + 0.5*P(Z1)[75]. From 30 confirmed parent-offspring relationships (19 with 'Bergeron', 7 with 'Canino' and 4 with 'Goldrich', Supplementary Data 1), the lowest pairwise *PI-hat* score was 0.5178 and the highest 0.588 (mean value of 0.551) (Supplementary Data 13). We considered all pairwise comparisons of accessions to be likely first-degree relatives if they had a *PI-hat* score ≥0.50 and retained one individual per pair of first-degree relatives (Supplementary Data 13).

After removing admixed individuals (i.e. individuals with a coefficient membership < 90% to a given gene pool, see above in 'Genetic subdivision among the Armeniaca species') and first-degree relatives (*PI-hat* score ≥0.50), we further reduced the size of the Central Asian Northern wild *P. armeniaca* (N_Par) and *P. mume* clusters to obtain balanced numbers of individuals across clusters; for this goal, we selected, at random, 44 individuals among Northern Central Asian wild *P. armeniaca* and four accessions for seven of the eight *P. mume* clusters (Supplementary Data 14). For the *P. mume* G1 cluster (Supplementary Figure 19), we retained only a single individual (SRR5046580) since the rest of the accessions of this genetic group were either clonemates or first-degree relatives (Supplementary Data 13 mumeG1). None of the Central Asian cultivated apricots were retained since they were admixed with wild *P. armeniaca* (Supplementary Figure 23b) or first-degree relatives of EIC apricots (Supplementary Data 13). From these filtering steps, we finally obtained a set of 202 unique Armeniaca accessions comprising 173 non-mume and 29 *P. mume* wild and cultivated accessions without close relatives.

Supplementary Note 12. Evolutionary history of apricots using random forest approximate Bayesian computation.

Genetic subdivision and variation among the set of Armeniaca unique accessions

We explored the relationships and genetic variation among the 202 unique accessions identified above. We inferred population structure with fastSTRUCTURE and we estimated genetic differentiation and variation among the genetic clusters identified with fastSTRUCTURE with: a principal component analysis (PCA) built with smartPCA[83], F_{ST} and Nei's *D* estimates[84], and a splitstree[85]. For those analyses, we removed non-synonymous SNPs as they can potentially be under selection. Non-synonymous SNPs were filtered out using SnpEFF software v4.3[86], and a total of 9,613 synonymous SNPs were kept for further analyses. fastSTRUCTURE analysis revealed seven distinct clusters (Supplementary Figure 25 and Supplementary Figure 21d for marginal likelihood), corresponding to European cultivated apricots (C1), *P. mume*, the wild *P. armeniaca* populations (W1 and W2) and the wild blue *P. sibirica* (W4), respectively. Chinese apricot cultivars (CH) and the wild W3 *P. sibirica* were assigned to the same purple genetic cluster but highly admixed, except ten Chinese cultivars that were assigned with membership coefficient >90% to the purple cluster (Supplementary Figure 25 and Supplementary Figure 21 D). The principal component analysis (PCA) showed a high genetic differentiation of *P. mume*, a cluster of the Chinese apricot cultivars (CH) and the wild W3 *P. sibirica* and a cluster of the yellow and red *P. armeniaca* populations (W1 and W2) (Supplementary Figure 26)[83]. F_{ST} and Nei's *D* estimates (Supplementary Data 15) and the splitstree (Supplementary Figure 27) confirmed these relationships.

Defining populations for inferring the divergence and demographic history of wild and cultivated apricots using ABC-RF

We defined the populations used in the ABC framework based on the clusters detected with fastSTRUCTURE for K=7 for the 202 unique Armeniaca accessions (Supplementary Figure 25 and Supplementary Figure 21 D). We removed for ABC-RF analyses admixed individuals (*i.e.* individuals with a membership coefficient <90% for a given cluster for a conservative approach), that included the green *P. sibirica* population in North Western China (N=19, Supplementary Figure 25) and admixed Chinese cultivars. We therefore retained 163

individuals for demographic inferences: 25 European (C1) and 10 Chinese (CH) cultivated accessions, 33 and 43 Central-Asian accessions from W1 and W2 *P. armeniaca* natural populations, respectively, 23 wild *P. sibirica* from the W4 genetic cluster and 29 *P. mume* individuals (Figure 5a). Genetic diversity estimates for each population are provided in Supplementary Data 15.

Evolutionary scenarios to reconstruct the apricot evolutionary history using ABC-RF

We used approximate Bayesian computation[87] to unravel the evolutionary history of the cultivated and wild apricots. We tested the history of two types of key evolutionary events: i) the divergence of populations, and ii) the occurrence of gene flow among populations. We used the newly developed ABC method, based on machine learning and named random forest (ABC-RF), to perform model choice and parameter estimation[88-90]. This approach allows distinguishing among numerous and complex demographic models[88] by comparing groups of scenarios differing by specific types of evolutionary events instead of considering all scenarios separately[91]. We used a four-step nested ABC-RF approach to infer the following evolutionary events: 1) the speciation of the two most divergent wild apricots, pink *P. mume* and the blue wild *P. sibirica*, 2) the divergence of the red and yellow wild *P. armeniaca* populations, 3a) the domestication of the Chinese cultivated apricots, 3b) the domestication of the European cultivated apricots, 4) the relative timing of domestication between the European and Chinese cultivated apricots. For step 1, we made the assumptions described below to build the tested scenarios. For step 2, we used as backbone the evolutionary history inferred at the previous step and tested where the wild populations branched and whether gene flow occurred. The evolutionary histories of the Chinese and European cultivated apricots were first run as independent steps 3a and 3b, using as a backbone for both the evolutionary history inferred for the wild apricots at step 2. We then tested a final scenario including the two cultivated apricot populations using the evolutionary histories inferred in steps 3a and 3b. Such a nested ABC approach avoids comparing too complex models with too numerous populations and parameters to simulate, and is more powerful than testing individually all scenarios to disentangle the main evolutionary events characterizing demography and divergence[91].

All scenarios tested are described in the Supplementary Data 16 and Supplementary Figure 28 available at https://data.inrae.fr/dataset.xhtml?persistentId=doi:10.15454/A15AKV.

We built scenarios based on: prior historical and archeological information, the observed patterns of genetic differentiation, and population structure (Supplementary Figure 27 and Supplementary Data 16). *Prunus brigantina* was previously shown to be an outgroup of the *P. mume* / *P. armeniaca* group[5], divergent in the splitstree (Supplementary Figure 27), and highly genetically differentiated (Supplementary Data 15). Additionally, the number of reads mapped and SNPs called onto the *P. armeniaca* reference genome for the *P. brigantina* Illumina sequenced genomes was low compared to those for *P. mume* and *P. armeniaca*. Such a low number of reads mapped and thus SNPs called for *P. brigantina* further indicated a high divergence of *P. brigantina* genome from the *P. armeniaca* reference genome. We therefore removed *P. brigantina* from the ABC analyses. We kept the 9,613 common unlinked synonymous SNPs among *P. mume* and *P. armeniaca* to avoid biases associated with SNP calling onto the *P. armeniaca* reference genome.

On the phylogenetic trees (Supplementary Figure 27d), *P. mume* always appeared as the earliest diverging population followed by the blue wild North Eastern *P. sibirica* (W4). *Prunus mume* and the blue wild North Eastern *P. sibirica* (W4) also showed the highest genetic differentiation from other populations (Supplementary Data 15). For ABC step 1, we therefore inferred the history of divergence of *P. mume* and the blue wild North Eastern *P. sibirica* (W4), and tested the occurrence of gene flow between the two populations. For ABC step 2, we picked the most likely divergence scenario inferred in step 1, and then tested the origin of the two wild *P. armeniaca* populations that appeared genetically close in the phylogenetic and distance trees (red W1 and yellow W2, Figure 5a and Supplementary Figure 27bd). We tested all possible combinations of divergence histories of these two populations on the backbone of the divergence events inferred in step 1 (Supplementary Data 16). In step 2, we also tested three scenarios of gene flow: i) between each of the two wild *P. armeniaca* populations and *P. mume* (as inferred from the ABBA-BABA tests, Supplementary Figure 27c and Supplementary Data 17), ii) among all populations, *P. sibirica* included, and iii) the complete absence of gene flow involving the wild *P. armeniaca* W1 and W2 (Supplementary Data 17).

For ABC step 3, in parallel, we tested the evolutionary history of the cultivated apricots. We inferred the origin of the Chinese and European cultivated apricots in two separate ABC runs, with or without the occurrence of gene flow among wild and cultivated apricots (steps 3a and 3b, Supplementary Data 16). We took the most likely scenarios inferred at steps 3a and 3b, and ran simulations following scenarios of domestication including both the Chinese and

European cultivated populations, and testing for their relative timing of domestication, and the occurrence of gene flow among the two cultivated populations (step 4, Supplementary Data 16).

We designed four and 12 scenarios for sets 1 and 2, respectively, to reconstruct the evolutionary history of the wild apricots (Supplementary Data 16). We simulated 12 scenarios for step 3 to infer the evolutionary history of the cultivated apricots separately (six scenarios for each of steps 3a and 3b), for testing for the origin of the Chinese and European cultivated apricots, respectively (Supplementary Data 16), and four scenarios for testing the relative timing of divergence the two cultivated populations together (step 4, Supplementary Data 16). Each ABC step included tests for the two types of evolutionary events (history of divergence and the occurrence of gene flow). For each step, we compared, as much as possible, all possible scenarios regarding both divergence histories and gene flow possibilities without grouping (Supplementary Data 16).

Random-forest ABC simulations

We used ABCtoolbox [92] with fastsimcoal 2.5 [93] to simulate datasets of 9,613 synonymous SNPs. We inferred the following model parameters: divergence time between *X* and *Y* populations ($T_{X\text{-}Y}$), effective population size of population *X* ($N_{E\text{-}X}$), migration rate per generation between the *X* and *Y* populations ($m_{X\text{-}Y}$). The unit of divergence time in fastsimcoal2 is expressed in number of generations. We set prior distributions for historical and demographic parameters taking into account historical and available information from previous studies on apricots. We assumed a non-overlapping generation time of 10 years[94]. The boundaries of the uniform (or log-uniform) prior distributions are presented in Supplementary Data 18.

For each simulation, we computed the following summary statistics with arlsumstats under the ARLEQUIN Suite v3.5[95]: the number of sites with segregating substitutions for the population i (S_i, i = {1, 2, 3, 4, 5}). We also added summary statistics based on the joint frequency spectrum (JFS) between all pairs of populations[96,97] computed with a home-made script: the sites that are polymorphic in population *i*, but monomorphic in population *j*, and vice-versa ($S_{x1\_i\_j}$ and $S_{x2\_i\_j}$ respectively); the number of shared polymorphic sites between population *i* and population *j* (S_{si_j}); and the number of sites showing fixed differences between two populations *i* and *j* ($S_{f\_i\_j}$).

We performed 10,000 simulations per scenario. The ABC-RF analysis provides a classification vote representing the number of times that a scenario is selected as the best one among 500 trees in the constructed random forest. For each ABC step (Supplementary Data 16), we selected the scenario, or the group of scenarios, with the highest number of classification votes as the best scenario, or group of scenarios, among a total of 500 classification trees[98]. We computed the posterior probabilities and prior error rates (i.e. the probability of choosing a wrong group of scenarios when drawing model index and parameter values into the priors of the best scenario) over 10 replicated analyses[91] for each ABC step.

We used the abcrf v.1.7.0 R statistical package[88] to conduct the ABC-RF. We also checked visually that the simulated models were compatible with the observed dataset by projecting the simulated and the observed datasets on the two first linear discriminant analysis (LDA) axes with the abcrf R statistical package[88] and checking that the observed dataset fell within the clouds of simulated datasets. We then performed parameter inferences using the final selected model following the three-round ABC procedure. Note that the ABC-RF approach includes the model checking step that was performed *a posteriori* in previous ABC methods.

Independent domestication events of apricots inferred with ABC-RF

For all ABC-RF steps, the projection of the reference table datasets and the observed dataset on a the two LDA axes that explained most of the summary statistics variance showed that the observed data fell in (steps 3 and 4, Supplementary Figure 30), or were close (steps 1 and 2, Supplementary Figure 29), to the distribution of the simulated summary statistics, that formed distinct clouds for each scenario or groups of scenarios. This visual inspection of the LDA plots indicated high power to discriminate and choose among scenarios that were further validated by the ABC-RF inferences presented below.

For all ten replicates, the ABC-RF step 1 supported an early divergence of *P. mume,* and the blue wild *P. sibirica* from an ancestral population, with the occurrence of gene flow among populations (scenario sc3, average of 380 votes out of the 500 RF-trees; posterior probabilities=84.4%, prior error rate = 35.5%, Supplementary Figure 29a, Supplementary Data 16).

Using as a backbone the supported scenario selected in step 1 (Supplementary Figure 29a), the ABC-RF step 2 supported for all ten replicates a divergence of the blue wild *P. sibirica* and the yellow wild *P. armeniaca* population (W2), followed by a divergence of the blue wild *P. sibirica* and the red wild *P. armeniaca* population (W1), without the occurrence of gene flow among any populations (scenario sc313, average of 118 votes out of the 500 RF-trees; posterior probabilities=62%, prior error rate = 13.6 %, Supplementary Figure 29b, Supplementary Data 16).

Using as backbone the supported scenario selected in step 2 (Supplementary Figure 29), the ABC-RF step 3a supported for seven of the ten replicates a divergence of the cultivated Chinese apricots from the red wild *P. armeniaca* population (W1) with the occurrence of gene flow between each of the two *P. armeniaca* populations and the blue wild *P. sibirica* population after the divergence (scenario sc309_g2_CHN_2_GF, average of 175 votes out of the 500 RF-trees; posterior probabilities=100%, prior error rate = 0.02 %, Supplementary Figure 30a, Supplementary Data 16). Using as backbone the most supported scenario in set 2, the ABC-RF step 3b supported for all ten replicates a divergence of the cultivated European apricots from the yellow wild *P. armeniaca* population (W2) with the occurrence of gene flow between each of the two *P. armeniaca* populations and the blue wild *P. sibirica* population after the divergence (scenario sc309_g2_C1a_3_GF, average of 244 votes out of the 500 RF-trees; posterior probabilities=95%, prior error rate = 0.11 %, Supplementary Figure 30b, Supplementary Data 16).

Using as backbone the supported scenarios selected in steps 3a and 4b, the ABC-RF step 4 supported for all ten replicates a divergence of the Chinese cultivated apricots before the European cultivated apricots, with the occurrence of gene flow between the cultivated European and Chinese apricots (Sc309_g2_CC_1_GF, average of 222 votes out of the 500 RF-trees; posterior probabilities=95.4%, prior error rate = 0.008 %, Supplementary Figure 30c, Supplementary Data 16).

Altogether ABC-RF supported an evolutionary history of wild apricots with gene flow among diverging lineages (except between the blue *P. sibirica* and the red wild *P. armeniaca* W2 and the blue wild *P. sibirica* W4), with successive divergence of *P. mume* and the blue wild *P. sibirica* population, and then of the red wild *P. armeniaca* populations (W1) from the blue wild *P. sibirica* lineage (W4), followed by a divergence of the yellow wild *P. armeniaca*

populations (W2) from the blue wild *P. sibirica* lineage (W4). ABC-RF inferences also supported the occurrence of gene flow during apricot domestication and independent domestication events of the two cultivated populations: Chinese cultivated apricots diverged from the red wild *P. armeniaca* populations (W1) while the European cultivated apricots diverged from yellow wild *P. armeniaca* populations (W2) populations. ABC-RF inferences also showed that the Chinese cultivated apricots diverged before the European cultivated apricots. Parameter estimates are provided in Figure 5a and Supplementary Data 16.

Supplementary Note 13. Identification of signatures of selection associated with apricot domestication.

We investigated patterns of selection in the European (C1) and Chinese (CH) genomes. We eliminated the admixed apricot genotypes based on the population structure analysis (Supplementary Figure 20, *N*=555). Finally, 136 individuals comprising 33 red (W1) and 43 yellow (W2) wild *P. armeniaca* from Central Asia that were used for Bayesian computation, the 10 non-admixed Chinese cultivars and 50 European cultivated apricots (Supplementary Data 14) were chosen for the investigation. The 50 European individuals are a mixture of the EIC A to D groups as depicted in Supplementary Figure 20, we selected individuals with coefficient membership ⩾ 0.9 from the whole IBD related or unrelated set of European cultivars. The initial set of 15,111,266 SNPs was filtered for SNPs with minor allele frequency <0.01 [--*maf=0.01*] with vcftools (version 0.1.16) and for LD > *0.0428* [--*indep-pairwise= 50, 5, 0.0428*] with PLINK V1.9[75]. It resulted in a dataset of 517,817 SNPs which was used for selective sweep analyses.

Model of demography history among the cultivated and wild apricots using SMC++

We used the Sequential Markovian Coalescent Prime (SMC) algorithm implemented in SMC++ (version v1.15.2)[99], to infer the historical demographics of cultivated (C1 and CH) and wild apricots (W1 and W2). From this we retrieved estimates of initial population size, population size changes and divergence time to be used as a background demographic effect for selective sweep analyses. We assumed a generation time of five years and a mutation rate of 4.46 E^{-9} mutations per nucleotide per year as previously estimated for *P. sibirica*[100]. The vcf2smc function was used to generate the input file and all estimations were performed by running the following command example: # smc++ estimate [4.6e-9] chr*.169_w1_33_smc.gz --timepoints

[30 100000]. The parameter <--timepoints> specified the demographic scale in terms of generations in SMC++.

The *N*e dips we observed could be associated with evolution and domestication events for wild and cultivated apricots. SMC++ showed a decrease in population size for the four apricot groups. However, while Chinese cultivated apricots experienced a continuous reduction of N_e since the last glaciation maximum (Supplementary Figure 31), wild Central Asian apricots encountered an expansion of their population sizes before decreasing steadily (red and orange lines). There were in fact two major fluctuation peaks in the wild Central Asian (W1) and cultivated European apricots (C1), one that expanded at the beginning of the LGM period and the second, declined in the 14-thousand-year period that followed the last glaciation maximum. The rapid increase of European population size N_e (up to a maximum of $7.5e^5$ about 1,6~9,02 Kya) coincided with the European apricot domestication time estimated by Liu *et al*[3] (~3.98 Kya) and in the current study (between 3 and 2 Kya, Figure 5). The ancestral effective population size of Chinese cultivated apricots (purple line in Supplementary Figure 31) reached $5.8e^4$ around 2.43 Kya, and then underwent a decline. SMC++ inferred a steady increase of the population size of the Chinese cultivated apricot ancestor after the last glaciation maximum. Over the last 1,000 years, the effective population size of Chinese and European cultivated apricots dropped to $4.3e^3$ and $1.62e^3$, respectively while the effective population size of Central asian apricots (W1 and W2) decreased down to $2.29e^3$ and $7.17e^2$ (Supplementary Figure 31).

Tests used to detect signatures of selection

A selective sweep results from natural selection acting on a locus, making the beneficial allele rise in frequency, leading to one abundant allele (the selected variant), an excess of rare alleles and increased LD around the selected locus. For detecting positive selection, we therefore used the composite-likelihood ratio test (CLR) and the Tajima's *D*, that detect an excess of rare alleles in the site-frequency spectrum (SFS). We also used the McDonald-Kreitman test (MKT), that detects more frequent non-synonymous substitutions than expected under neutral evolution (Supplementary Data 19 for raw data; Supplementary Data 20 for annotation of selective sweeps among the top 0.5% CLR values). In addition, we looked for regions of increased LD. However, selection scan approaches based on haplotype frequency can be strongly affected by population demography, as population expansion can also lead to an excess of rare variants.

We therefore analysed population size variation across time (Supplementary Note 13). With a mutation rate estimate of 4.46 E^{-9} mutations per generation and a generation time of five years, we inferred population bottlenecks over the last 15,000 years that corresponded with domestication and reduced areas of natural populations (Supplementary Figure 31, see above section). The inferred history was further supported by ABC-RF (Figure 5). SMC++ generated demography parameter estimates (N_e, bottleneck, number of generations since domestication) were integrated in the CLR computation for controlling for demographic effects in the site-frequency spectrum.

To identify the signals of positive selection along the wild and cultivated apricot genomes, we used SweeD, which implements a composite likelihood ratio (CLR) test to detect hard selective sweeps by identifying an excess of rare alleles in the site frequency spectrum (SFS) of single-nucleotide polymorphisms (SNPs)[101]. We also computed ratios of variation within each group (polymorphism) to divergence between groups (substitutions) at two types of sites, neutral (usually synonymous coding sites) and non-neutral (non-synonymous coding sites) through the McDonald-Kreitman test (MKT) as implemented in PopGenome[84]. Genome-wide genetic diversity was estimated by calculating pairwise nucleotide differences within populations, *Pi*(π)[102] and Tajima's D[103]. Genetic diversity was then computed as a ratio of $\pi_{\mathrm{wild}}/\pi_{\mathrm{cutlivated}}$. The genetic differences F_{ST} and D_{XY} between wild and cultivated apricots were calculated in non-overlapping windows of 10 Kb in size with vcftools (v0.1.16)[104]. *Pi* (π), F_{ST} and D_{XY} were calculated in PopGenome[84]. We also used Omegaplus, a high-performance implementation of the ω statistic, to detect increased signals by linkage disequilibrium (LD)[105]. All selective sweep analyses focused on sliding 10-Kb windows, reflecting the genome-wide pattern of linkage disequilibrium decline and the maximum decay distance (Supplementary Note 9 Linkage disequilibrium analysis).

Parameters used for each test

The alignment position for CLR calculation was specified in <-*grid*> function by a given value that was defined as the total number of 10 Kb non-overlapping windows in one certain chromosome. Both are from the Exelixis Lab, *Omegaplus* software (v2.0.0) displays execution commands similar to SweeD (v3.0.0), with the difference in setting the LD measures as follows: [-minwin=100], [-maxwin=5000]. The Mcdonald-Kreitman test was run with the *mkt*() function in

PopGenome R package[84], the chromosomal Marouch #14 transcripts gff3 files were used to assign SNP information to each coding region as defined in the GFF files. The neutrality index (NI) quantifies the direction and degree of departure from neutrality, a neutrality index greater than 1 indicates negative selection, while a neutrality index lower than 1 indicates positive selection. The Dxy, *Pi* (π) and F_{ST} tests were calculated by *get.diversity*() function in PopGenome R package[84] and summarized within non-overlapping windows of 10 Kb along the chromosome. Tajima's *D* was measured in vcftools (version 0.1.16) following the same rules as above.

All scripts used to run the above tests are available under the link https://forgemia.inra.fr/amandine.cornille/apricot_evolutionary_history_2021

Data filtering

We defined different levels of thresholds to select highly significant selective sweeps: (i) at top 0.1%, 0.5% and 1% of CLR, ω, D_{XY}, *Pi* (π) and F_{ST} scores, (ii) at lowest 0.1%, 0.5% and 1% of Tajima's *D* values. Furthermore, the simulated demographic populations derived from SMC++ were also used to define a cut-off value for CLR, ω, *Pi* (π) and Tajima's *D*, any region with a score less than this cut-off is possibly confounded with a demographic effect. Raw data as well as cut-off values are displayed in Supplementary Data 19 (available at https://data.inrae.fr/dataset.xhtml?persistentId=doi:10.15454/A15AKV).

Genomic signatures of selection

Composite likelihood ratio (CLR) tests identified 856 and 450 selective sweep regions in the genomes of cultivated European and Chinese apricots, respectively (0.42% and 0.22% of the genome affected, respectively; Supplementary Data 20 and 21). The selective sweep regions did not overlap at all between the European and Chinese cultivated populations, suggesting the lack of parallel selection on the same loci despite convergent phenotypic traits. Using the top 0.5% of CLR scores as a threshold for European apricots, we detected 54 regions under selection, half of which were located over the middle position of chromosome 4 (from 7 Mbp to 18 Mbp), indicating a potential hotspot of human selection targets (Figure 6a and 6b) since

only 12 selective sweeps would be expected on chromosome 4 under the assumption of a random distribution of selection targets across the genome. In Chinese apricots, one-third of the selective sweeps mapped onto chromosome 1 and no particular enrichment was observed for chromosome 4 (Figure 6c and 6d).

We also used Tajima's *D,* where peaks of negative values in genomes indicate an excess of rare alleles and thus positive selection, and linkage disequilibrium LD estimates along the genomes since selective sweeps increase LD locally[106]. Because LD decay in apricots was <1Kb (Supplementary Figure 17), the genome scans were performed in non-overlapping windows of 10 Kb. *Pi* (π), Tajima's *D* and LD are less robust than CLR and MKT in detecting positive selection, therefore, for a gene annotated region to be detected as evolved under positive selection at least two of the different selection tests, CLR and MKT included needed to identify the gene (Supplementary Data 20). For European apricots, using as a threshold the top 0.5% of $\pi_{\mathrm{Wild}}/\pi_{\mathrm{Cultivated}}$, Tajima's *D* or LD, we identified 310 regions under positive selection (Supplementary Data 19), 33 of which were shared between at least two tests, most of them being detected by either CLR or MKT (Supplementary Data 20 and 21, Supplementary Figure 32). In Chinese apricots, we detected 292 regions, of which 15 were shared between at least two tests (Supplementary Data 2, Supplementary Figure 32). Finally, based on the McDonald-Kreitman (MK) test, we identified 88 loci evolving under positive selection in the European cultivars and only 15 in the Chinese apricots (Supplementary Data 21).

We also compared differentiation between cultivated populations and their genetically closest wild populations through the population differentiation-based tests (F_{ST}, D_{XY} computed with the PopGenome R package[84]) to detect genomic regions more differentiated than genome-wide expectations. With the top 0.5% values of the two pairwise statistics (F_{ST}, D_{XY}), we identified 294 10Kb-long genomic regions as evolving under divergent selection in the genome of cultivated European apricots and 196 regions in the Chinese, cultivated apricots (Supplementary Data 19). Only four regions overlapped between the F_{ST} and D_{XY} tests, both for European and Chinese cultivated apricots (Supplementary Data 20 and 21).

Supplementary Note 14. Candidate gene identification.

Gene ontology analysis

Gene ontology (GO) annotation terms were extracted from the annotated Marouch #14 genome and used to construct three GMT files (Gene Matrix Transposed file format, https://software.broadinstitute.org/cancer/software/gsea/wiki/index.php/Data_formats#GMT:_Gene_Matrix_Transposed_file_format_.28.2A.gmt.29) corresponding to the three compartments of the GeneOntology (Biological Process, Molecular Function and Cellular Component). The list of candidate genes mapping in selective sweeps intervals were analysed against these Marouch #14 GMT files with the library R Gprofiler2 v0.2.0 (https://cran.r-project.org/web/packages/gprofiler2/index.html) and the results were filtered by the Benjamini-Hochberg multiple test correction (p-adjusted < 0.05). Results of GO enrichment are displayed in Supplementary Data 22 for European apricots and Supplementary Data 23 for Chinese apricots. The Venn diagrams (Supplementary Figure 32) show the 10Kb intervals unique for each test or shared by at least two tests.

Gene enrichment in the regions under selection

Among the genes within the top 0.5% highest values of F_{ST} and D_{XY}, we found, as enriched functions, components of the auxin metabolic process and histone methyltransferase activity for European apricots (Supplementary Data 22), and DNA integration and triterpenoid biosynthesis for Chinese apricots (Supplementary Data 23). Only four regions overlapped between the F_{ST} and D_{XY} tests, both for European and Chinese cultivated apricots (Supplementary Data 20). The Chr7:18120001..18130000 locus encompassing a malate dehydrogenase that accumulated non-synonymous mutations (see MKT results in Supplementary Data 20) was also identified as significantly differentiated between European cultivars and both Southern and Northern wild *P. armeniaca* natural populations (Supplementary Data 20). This same locus (MacDonald and Kreitman test) was also identified as significantly differentiated between European cultivars and both Southern and Northern wild *P. armeniaca* natural populations (F_{ST} and D_{XY}, Supplementary Data 20).

A major effect of human selection on chromosome 4 during the domestication of European apricots

Selective sweep regions were associated with QTLs identified previously in apricots, *P. mume*, peaches and cherries (https://www.rosaceae.org/search/qtl). We examined overlaps between known QTLs (Quantitative Trait Locus) identified by GWAS (Genome Wide Association Studies) or linkage mapping and the genomic regions with footprints of selection identified by the above seven tests. The middle region on chromosome 4 appeared to be significantly enriched in selective sweeps (Supplementary Data 20). The region (Chr4:13280001..13290000) identified by the MK test, the CLR and Tajima's *D* encompassed a major QTL for chilling requirement and leafing date in *P. mume*[107], blooming date in apricot[108] and in peach[109] (Figure 6b). This 10-Kb interval contained two genes identified by association studies as likely involved in important traits, one for the establishment of winter dormancy, *i.e.* the WDR5 homolog COMPASS-like H3K4 histone methylase[110] and the other one for fruit aroma volatile biosynthesis, the pyruvate decarboxylase protein[111] (Figure 6b). Through π_{W1}/π_{C1} or π_{W2}/π_{C1} ratios, the MK test and F_{ST}, we identified another physically close selective sweep interval (chr4:15970001..15980000) that co-localised with the major QTLs for apricot fruit firmness[112] and peach ripening[113]. In sweet cherry (*Prunus avium*), the major QTLs for fruit development time, maturity date, firmness, and soluble solids content were located over the same narrow region of linkage group 4[114]. Interestingly, the locus displays two genes previously associated with ripening and softening of strawberry and tomato fruits, the NAC (NAM/ATAF 1,2/CUC2) like proteins[115,116] and apple and cherry fruit firmness[117,118]. A few megabases upstream, two consecutive intervals from 12.96 to 12.98 Mbp harbored signatures of selection identified with CLR, Tajima's *D* and π_{W2}/π_{C1} or π_{W1}/π_{C1} ratios encompassing five copies of the *Nerolidol Synthase1* gene coding for (-)-alpha-pinene synthase. In strawberry, this gene is involved in fruit flavour and an insertional mutation caused the depletion of these compounds in the cultivated strawberries[119]. As indicated by the π_{W2}/π_{C1} or π_{W1}/π_{C1} ratios and composite likelihood analysis (CLR), a cluster of five copies of MDHAR (monodehydroascorbate reductase) displayed a 20kb selective sweeps in the European cultivated apricot genomes (Supplementary Data 24). MDHAR activity is necessary to maintain yield in *cherry* tomato[120] and is tightly linked to glutathione turnover as part of the glutathione-ascorbate cycle in fruits[121], that is consistent with the functional annotation presented above (Supplementary Data 20). We also identified by CLR, MKT and π, an upstream region on chromosome 4, around 8Mbp, enriched in selective sweeps, and with genes associated with flowering and fruit development (Figure 6b; Supplementary Data 24). The apricot linkage group 4 from 7 Mb to 18 Mb is thus a hotspot for important phenology traits, bloom and maturity date and for fruit

quality traits (ripening, firmness, aroma) in cultivated apricots, as previously suggested in cherry and peach[117,122].

Extraction of candidate gene sequences and haplotype phasing

To determine, for a set of candidate genes (Supplementary Data 24), the haplotypes fixed during domestication in European and Chinese apricots and their geographic distribution over their cultivation areas, we retrieved the vcf files described in the Supplementary Note 9. Each resulting VCF entry displayed a diploid genotype field consisting of two alleles in the set {0,1,2} separated by either '/' or '|'. The space value is [0,1,2]/[0,1,2] or [0,1,2]|[0,1,2] and the maximum likelihood expectation (MLE) for the allele counts (MLEAC) > 0. This was performed by using the executable vcffilter from vcflib (https://github.com/vcflib/vcflib) and custom scripts. The extraction of haplotype-relevant information was performed with extractHAIRS from HapCUT2 tool v1.2[123]. The resulting fragment files were assembled into haplotype blocks with HapCUT2. The output files were converted to vcf format with the HapCutToVcf tool included in fgbio (https://github.com/fulcrumgenomics/fgbio) then compressed and indexed with bcftools 1.9 (https://github.com/samtools/bcftools). The genome coordinates of 25 loci of interest extended by 500 nt 5' and 3' was used to retrieve the haploid 1 (P1) and 2 (P2) fasta sequence from each locus (Custom script and bcftools 1.9). The corresponding sequences of these loci were also extracted from the Marouch #14 reference genome by using samtools 1.9 and custom scripts. Another custom script was used to interleave P1/P2 fasta sequences and merge all fasta for the same locus with the Marouch #14 reference genome.

Biogeographical distribution of candidate gene haplotypes

For each candidate gene, variants from individual vcf files were processed using bcftools version 1.10 (https://github.com/samtools/bcftools). They were i) normalized into bi-allelic sites, ii) annotated with custom ID, based on their chromosome location, reference sequence and alternate variation, and iii) merged and compressed into a single file to be indexed. Prior to running PCA, the merged variants file was converted into PLINK compatible formats using PLINK version 1.9[75]. The principal component analysis (PCA) was then carried out using smartpca utility in the EIGENSOFT software version 7.2.1[83]. Based on the first ten PCs, we

grouped individuals using hierarchical clustering (Euclidean distance and Ward method) and drew optimal partitions using the factoextra R package (https://CRAN.R-project.org/package=factoextra). Within each cluster, we identified cluster representatives as the closest individual haplotypes to their cluster centroïd. Finally, we explored haplotype distribution and visualized it at the geographical level using the scatterplot3d R package[124] (R Development Core Team, URL) (Figure 7). Principal component analysis, clustering and world map plot results were summarized into interactive reports using a custom R script based on R markdown (https://github.com/rstudio/rmarkdown). This pipeline was incorporated into a reproducible workflow management system, Snakemake version 5.19.2[125].

Supplementary Note 15. Tools and scripts for genome assembly and population genomic analyses.

Tools used in this study for genome assembly

SMARTdenovo (git commit 5cc1356) https://github.com/ruanjue/smartdenovo

Filtlong (v0.2.0, git commit cf65a48) https://github.com/rrwick/Filtlong

RaGOO (v1.1) https://github.com/malonge/RagTag

Falcon (v0.7) https://github.com/PacificBiosciences/FALCON

Falcon UNZIP (v0.7) https://github.com/PacificBiosciences/FALCON_unzip

Purge Haplotigs (v1.0.1) https://bitbucket.org/mroachawri/purge_haplotigs/src/master/

Arrow (v2.3.0) https://github.com/PacificBiosciences/GenomicConsensus

GapCloser (v1.12-r6) https://sourceforge.net/projects/soapdenovo2/files/GapCloser/bin/r6/

ALLMAPS (v0.8.4) https://github.com/tanghaibao/jcvi/wiki/ALLMAPS

Gprofiler2 (v0.2.0) https://cran.r-project.org/web/packages/gprofiler2/index.html

Tools used in this study for population genomic analysis

ABCtoolbox https://cmpg.unibe.ch/software/ABCtoolbox/

ARLEQUIN Suite (v3.5) http://cmpg.unibe.ch/software/arlequin35/

bcftools (v1.6) https://github.com/samtools/bcftools

BWA (v0.7.17) https://github.com/lh3/bwa/releases/tag/v0.7.17

cutadapt (v1.2.1) https://cutadapt.readthedocs.io/en/stable/

Dsuite (v0.3) https://github.com/millanek/Dsuite

eigensoft (v6.1.4) https://alkesgroup.broadinstitute.org/EIGENSOFT/

factoextra (v1.0.3) R package https://www.R-project.org

fastSTRUCTURE (v1.0) https://rajanil.github.io/fastStructure/
GATK (v3.8) https://gatk.broadinstitute.org/hc/en-us
NGS QC-toolkit (v2.3.3) http://www.nipgr.ac.in/ngsqctoolkit.html
OmegaPlus (v2.0.0) https://github.com/alachins/omegaplus/
Paup (v4.0a) https://paup.phylosolutions.com/
picard-tools (v2.9.2) https://broadinstitute.github.io/picard/
plink (v1.90) https://www.cog-genomics.org/plink2/
R markdown https://github.com/rstudio/rmarkdown
Ruby (v2.6.4) https://www.ruby-lang.org/en/news/2019/08/28/ruby-2-6-4-released/
SMC++ (v1.15.2) https://github.com/popgenmethods/smcpp
Sweed software (v3.0.0) https://app.assembla.com/spaces/sweed/git/source
vcftools (v0.1.16) https://github.com/vcftools/vcftools/releases/tag/v0.1.16

R packages

PopGenome (v2.2.4) https://cran.r-project.org/src/contrib/Archive/PopGenome/PopGenome_2.2.4.tar.gz
abcrf v1.7.0: https://cran.r-project.org/web/packages/abcrf
ggplot2 v3.6.2: https://cran.r-project.org/web/packages/ggplot2/
scatterplot3d v0.3-41: https://cran.r-project.org/web/packages/scatterplot3d
abc v2.1: https://cran.r-project.org/web/packages/abc

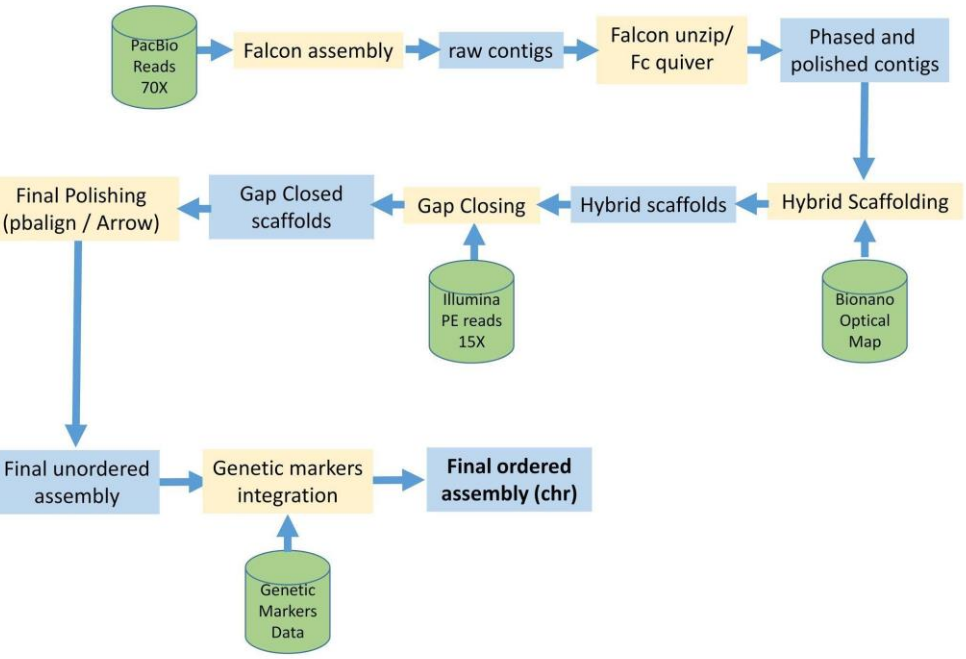

Supplementary Figure 1. The workflow for the genome assembly of *Prunus armeniaca* Marouch #14 and cv. Stella accessions

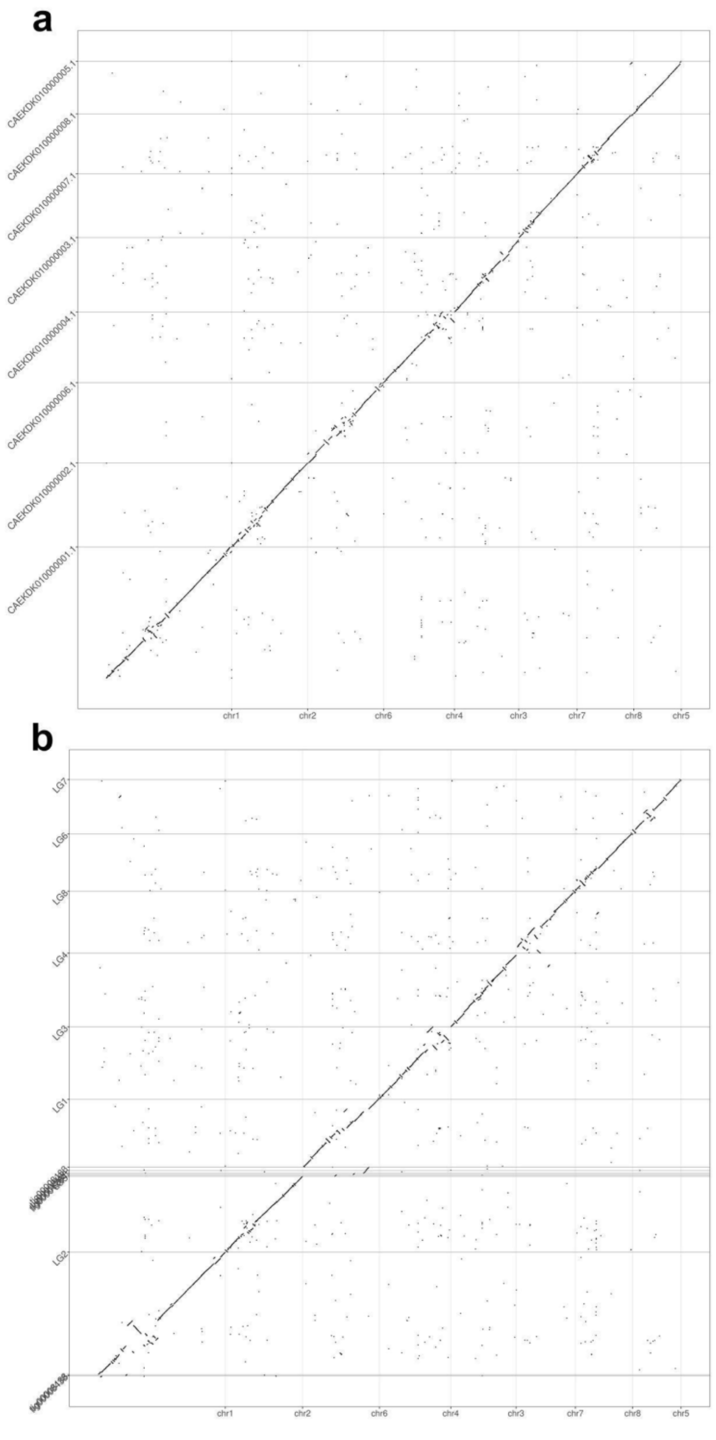

Supplementary Figure 2. Whole genome alignment visualization.

Alignments smaller than 5Kb were filtered out. **A.** The x-axis represents the *Prunus armeniaca* Marouch #14 chromosomes and y-axis the *P. armeniaca* cv. Rojo Pasion scaffolds. **B.** The x-axis represents the *P. armeniaca* Marouch #14 chromosomes and y-axis the *P. armeniaca* cv. Chuanzhihong scaffolds (short scaffolds are stacked together).

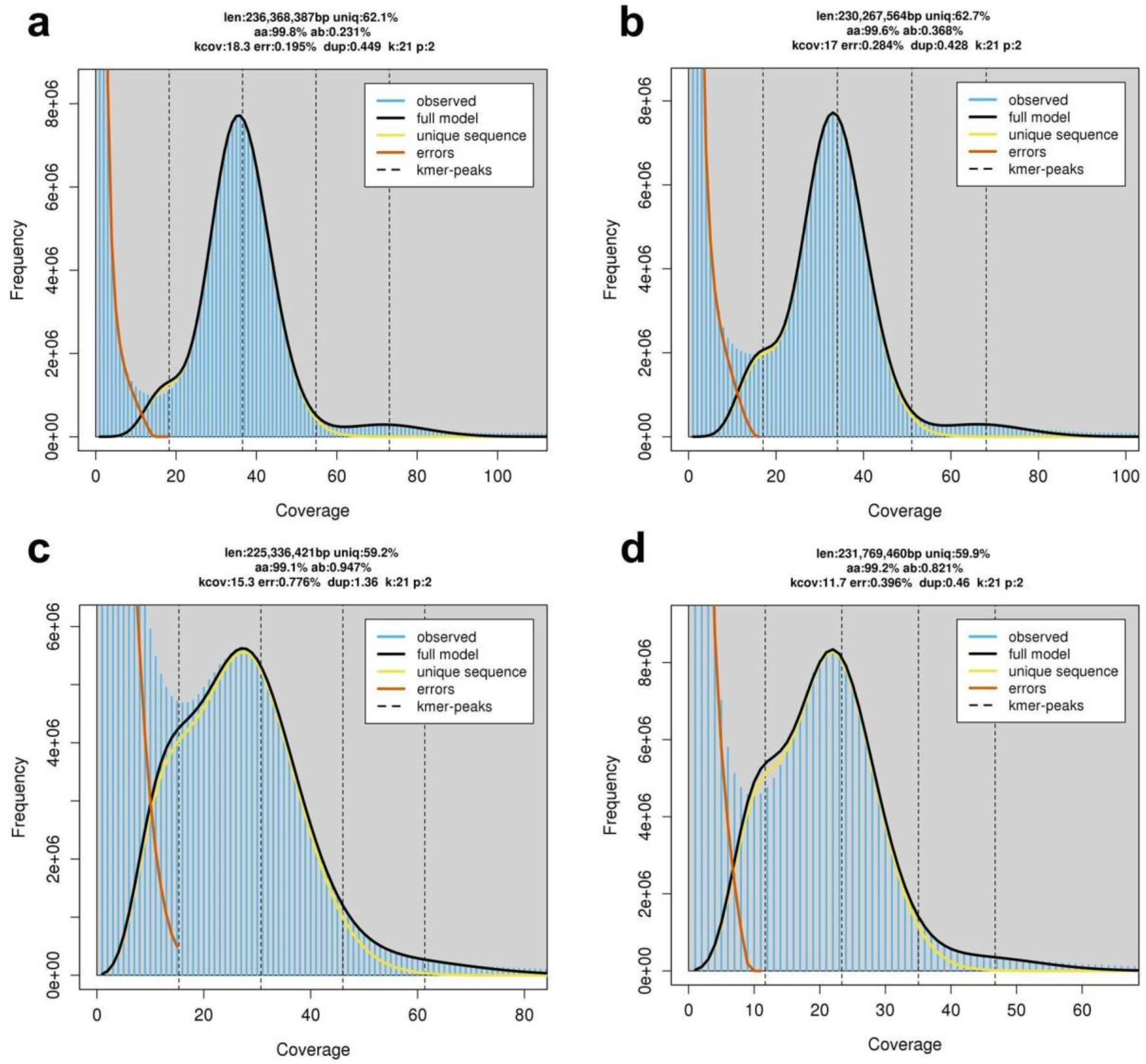

Supplementary Figure 3. GenomeScope plots for the four Armeniaca genomes.

K-mer spectra and fitted models for a. Marouch #14, b. cv. Stella, c. CH320_5 and d. CH264_4.

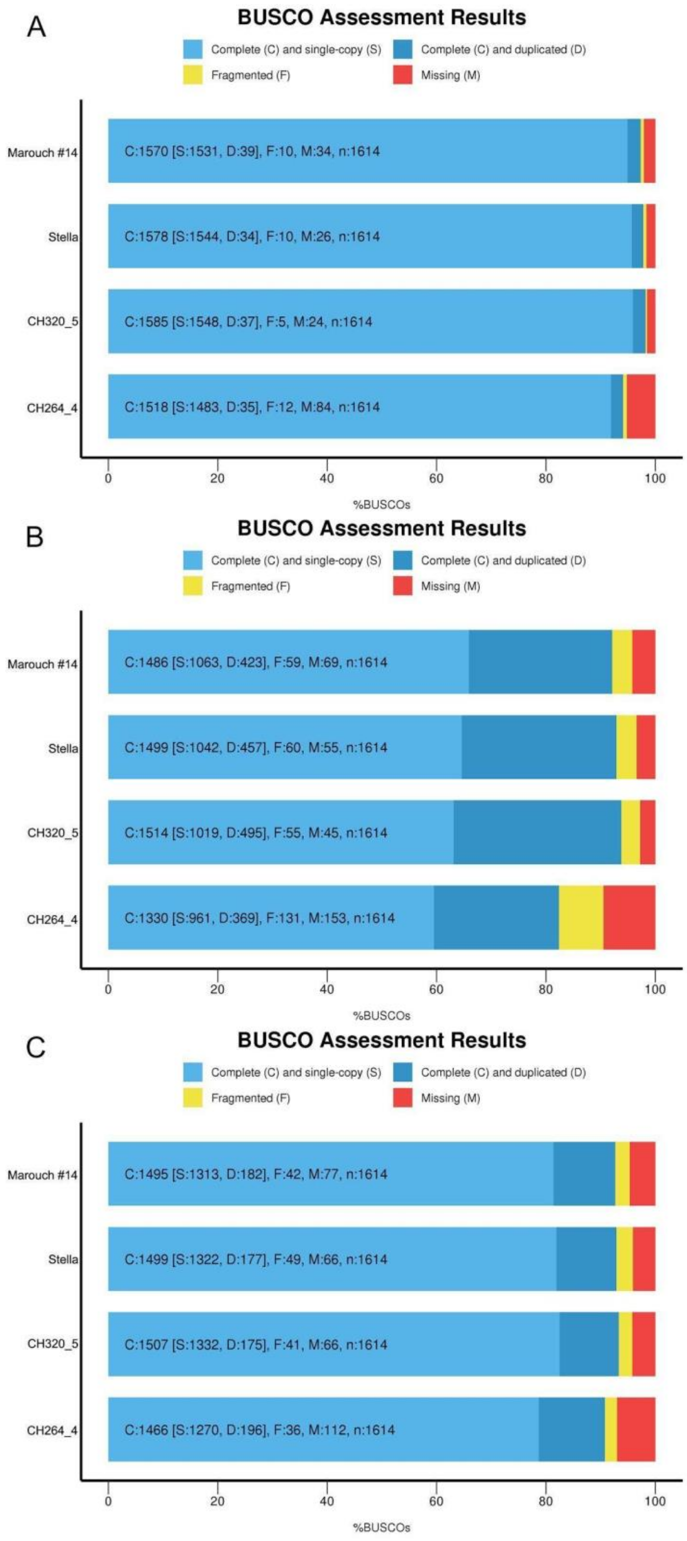

Supplementary Figure 4. Busco assessment results for Marouch #14, cv. Stella, CH320_5 and CH264_4. The different panels correspond to the following data: (A) assembled genomes, (B) assembled transcripts and (C) predicted proteins.

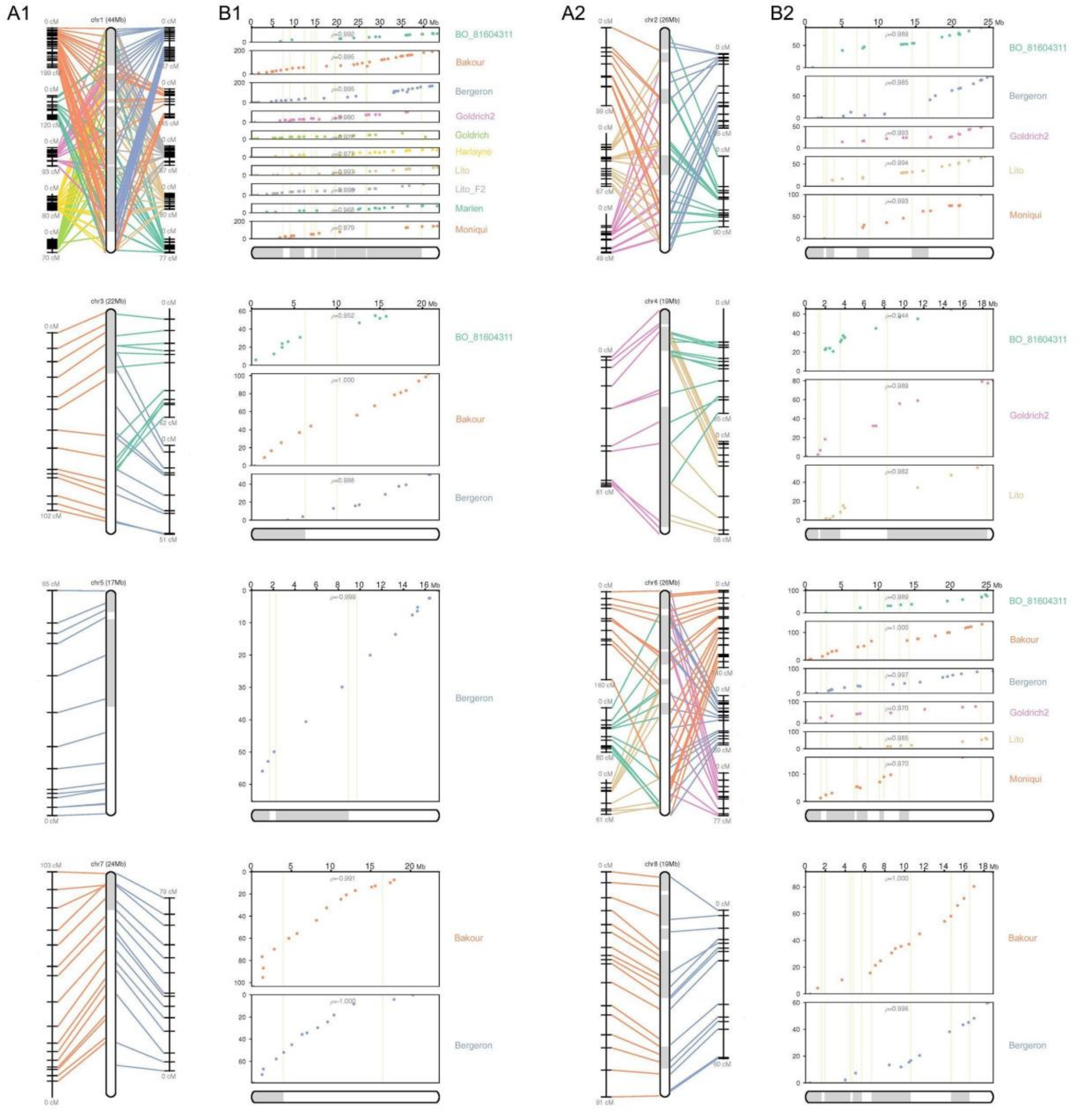

Supplementary Figure 5. Pseudochromosomes 1 to 8 of Marouch #14 genome, reconstructed from ten input maps, i.e. cv. Bakour, cv. Bergeron, BO81604311, cv. Harlayne, cv. Goldrich, self-pollinated cv. Goldrich2, cv. Harlayne, cv. Lito, self-pollinated cv. Lito2, cv. Marlén and cv. Moniqui with equal weights of 1.

(A1 and A2) CMAP-style presentation with lines connecting the physical positions on the reconstructed chromosome and the map positions. (B1 and B2) Scatter plots, with dots representing the physical position on the chromosome (x-axis) versus the map location (y-axis). Adjacent scaffolds within reconstructed chromosomes are shown as boxes with alternating shades, marking the boundaries of the component scaffolds. The ρ value on each scatter plot measures the Pearson correlation coefficient, with values in the range of -1 to 1 (values closer to -1 and 1 indicate near-perfect collinearity). Names and position of the molecular markers are depicted in Supplementary Data 6.

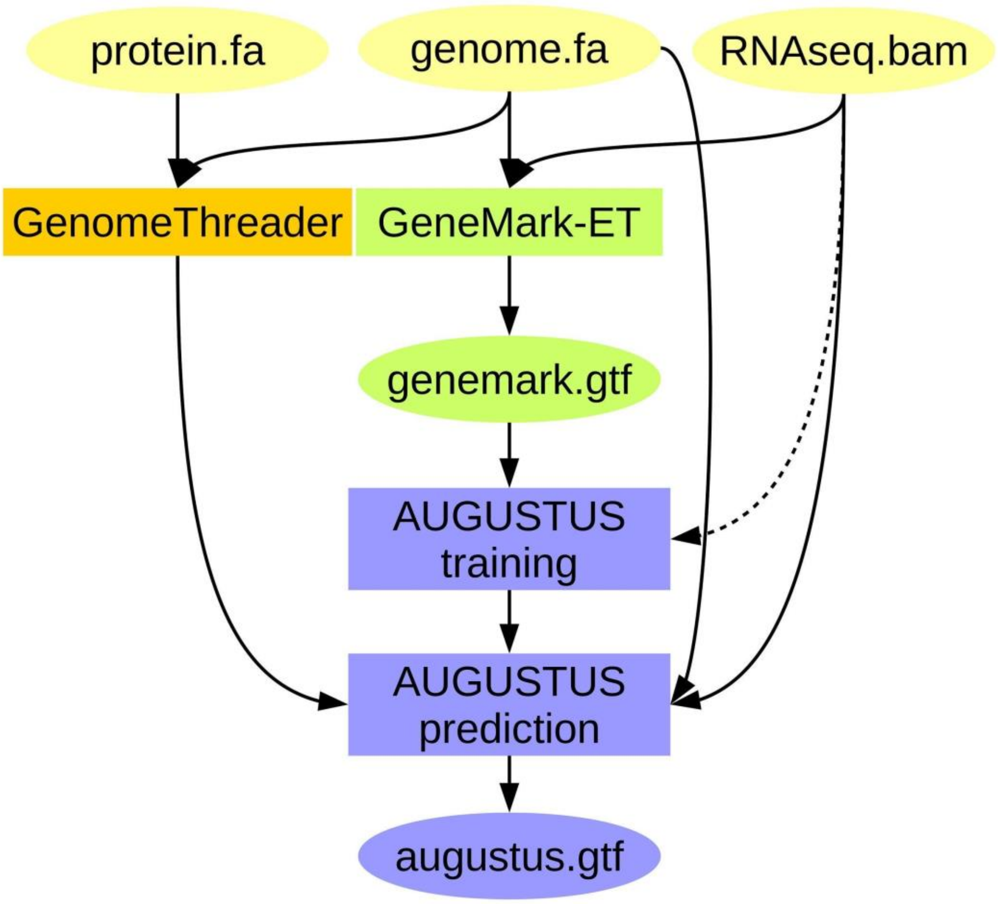

Supplementary Figure 6. BRAKER2 pipeline with GeneMark-EP+ and AUGUSTUS externally supported by cross-species protein sequences aligned to the genome[39].

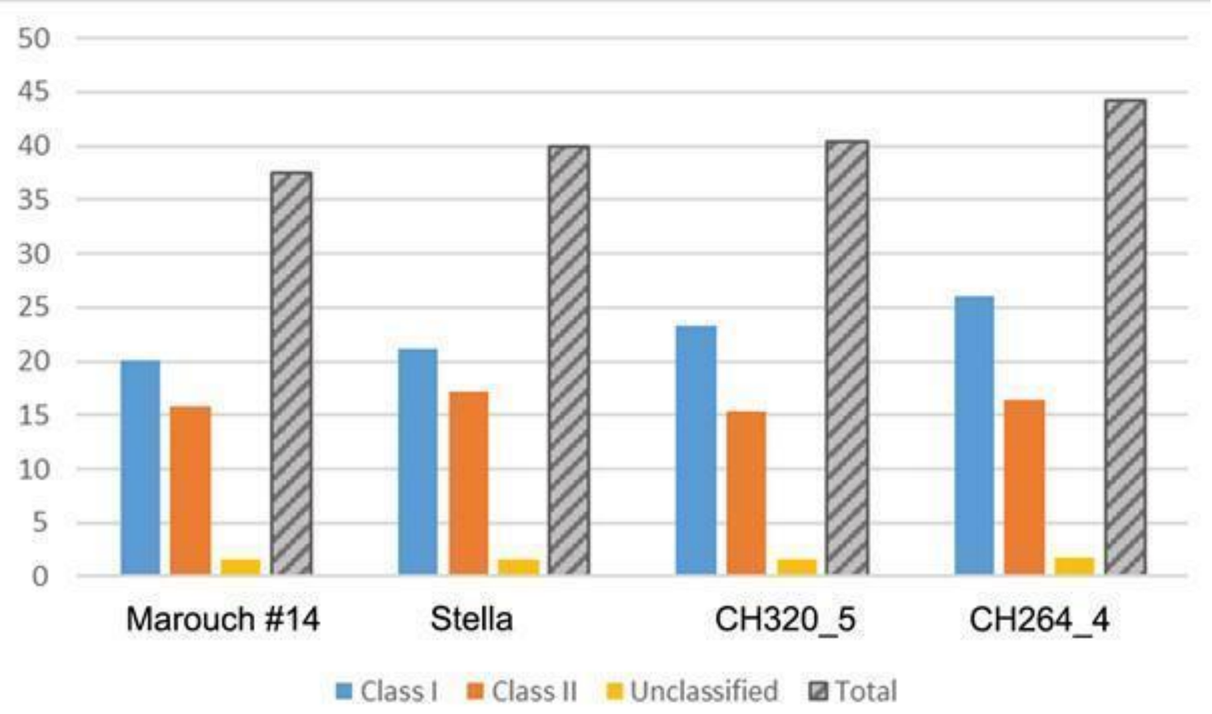

Supplementary Figure 7. Comparison of different transposable element (TE) class coverage in *Prunus armeniaca* (Marouch #14 and cv. Stella), *P. sibirica* (CH320_5) and *P. mandshurica* (CH264_4) genomes.

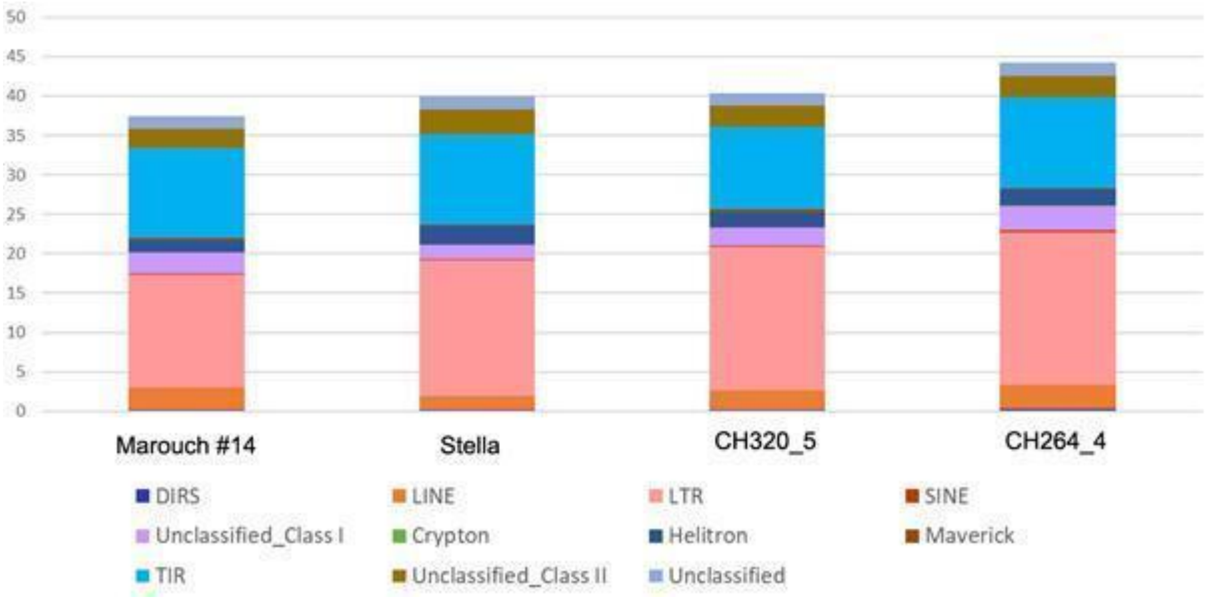

Supplementary Figure 8. The coverage percentage (%) of different transposable element superfamilies in *Prunus armeniaca* (Marouch #14 and cv. Stella), *P. sibirica* (CH320_5) and *P. manshurica* (CH264_4) genomes.

The proportion of each superfamily was calculated by the percentage of their total length in relation to the genome size. The superfamilies Dictyoctelium Intermediate Repeat Sequence (DIRS), Long interspersed nuclear elements (LINE), Long Terminal Repeats (LTR), Short interspersed nuclear elements (SINE) and Unclassified _class I are the class I repeat elements. The superfamilies Crypton, Helitron, Maverick, Terminal Inverted Repeats (TIR) and Unclassified _class II are the repeat elements belonging to the class II repeat elements. The unclassified category encompasses all repeat sequences without any clear featured classification.

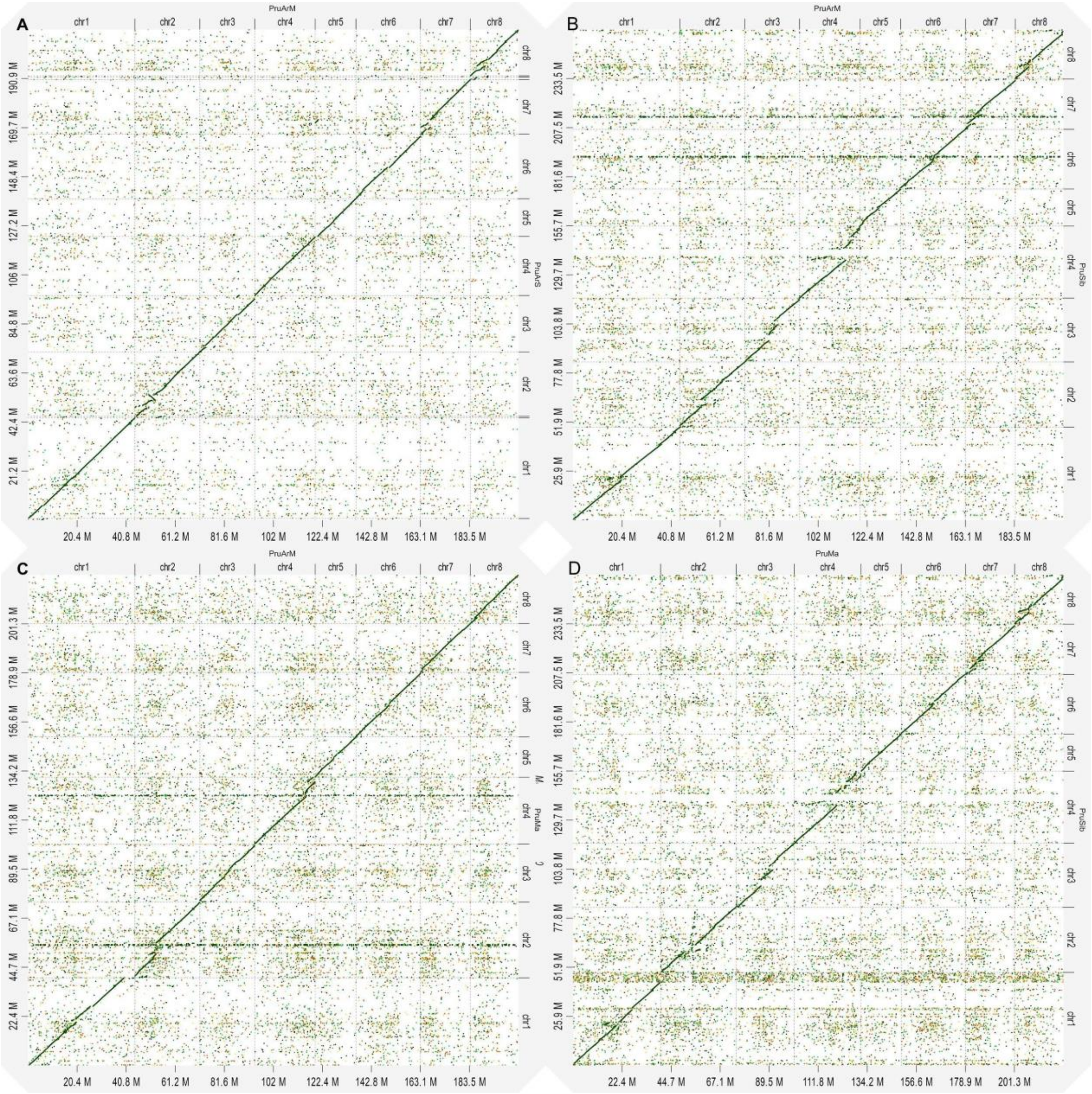

Supplementary Figure 9. Dot plots of genome alignments using minimap2 software package and generated with D-Genies[46].

(A) to (D) : cv. Stella (y-axis) vs Marouch #14 (x-axis), CH320_5 vs Marouch #14, CH264_4 vs Marouch #14 and CH320_5 vs CH264_4 respectively.

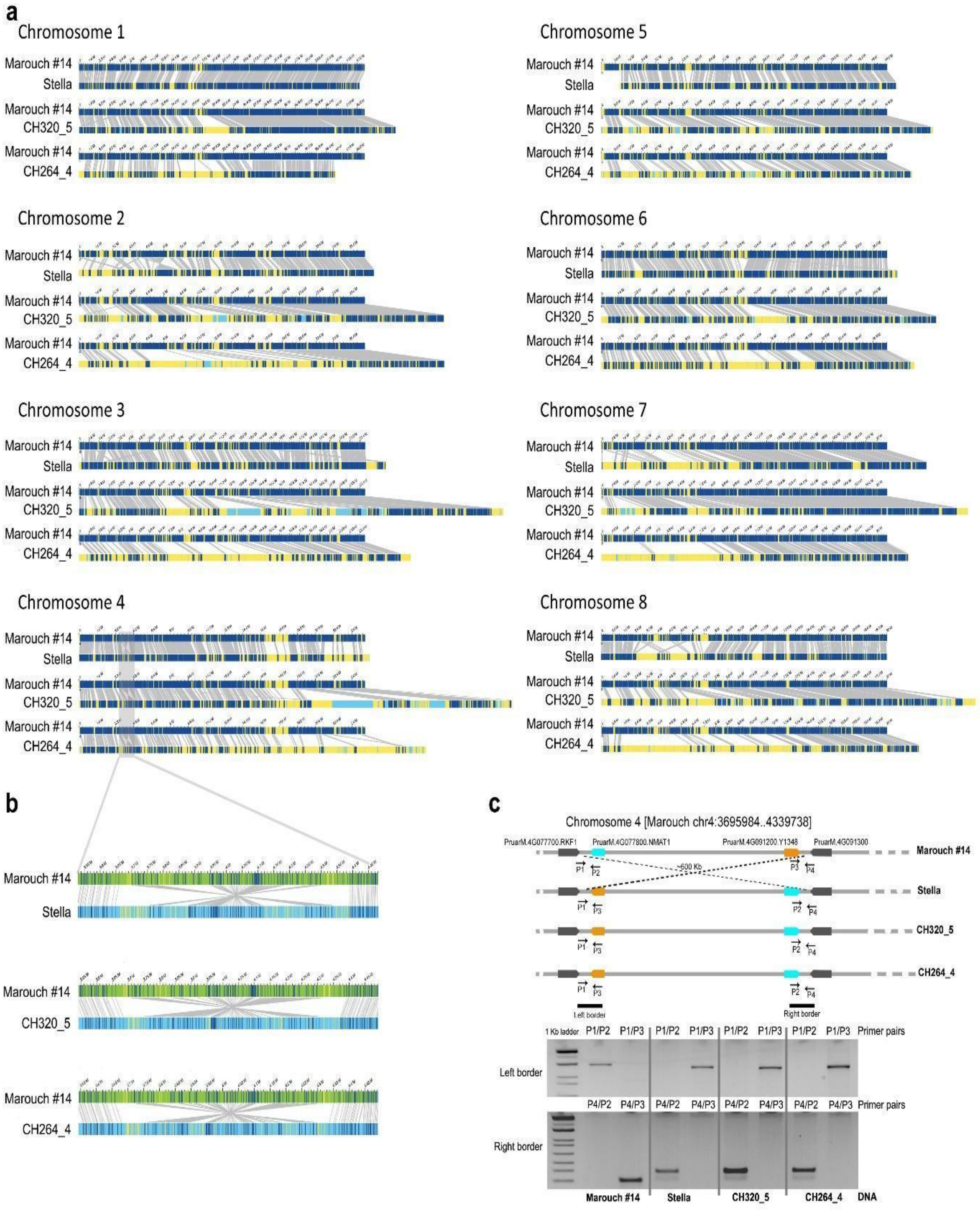

Supplementary Figure 10. Whole genome alignment of the three apricot genotypes (cv. Stella, CH320_5 and CH264_4) against Marouch #14.

a. Alignments are organised by chromosome and the genotype *Prunus armeniaca* Marouch #14 was used as reference and corresponds to the chromosome first line. The same order is conserved for each chromosome alignment and the genotypes *Prunus armeniaca* cv. Stella, *Prunus sibirica* (CH320_5) and *Prunus mandshurica* (CH264_4) are aligned, respectively, against the Marouch #14 chromosome homolog. The dark blue regions correspond to the genomic regions that could be aligned between the two genotypes. In yellow are the regions

that could not be aligned with other genomes. In light blue are genomic regions that could not be analysed due to large stretches of N introduced during the genome assembly scaffolding. **b**. Close-up of an inversion of ca. 600 kb detected in the *P. armeniaca* Marouch #14 genome when compared to *P. armeniaca* cv. Stella, *P. sibirica* CH320_5 and *P. mandshurica* CH264_4. The dark blue regions correspond to the genomic regions that could be aligned between the two genotypes. In yellow are the regions that could not be aligned with other genomes. **c.** Verification of the ca. 600 Kb inversion by polymerase chain reaction (PCR) by targeting the left and right borders. Primer sequences: P1 [AGCACGAGCTTCGCGTTT], P2 [ACCCTTGGGGTTTGGAATTA], P3 [TCACATCAACAAACCAGCAGA], P4_rev [AGGGCAGATCATTGGACAAA]. PCR conditions: 30 cycles with a melting temperature at 57°c and 1 minute of extension at 72°C. Each PCR was repeated twice from two different genomic DNA extracts. Left and right border fragments were loaded on two separate 1% and 2% agarose gels, respectively. 1Kb Ladder indicates molecular weight markers. Source data underlying Supplementary Figure 10c are provided as a Source Data file.

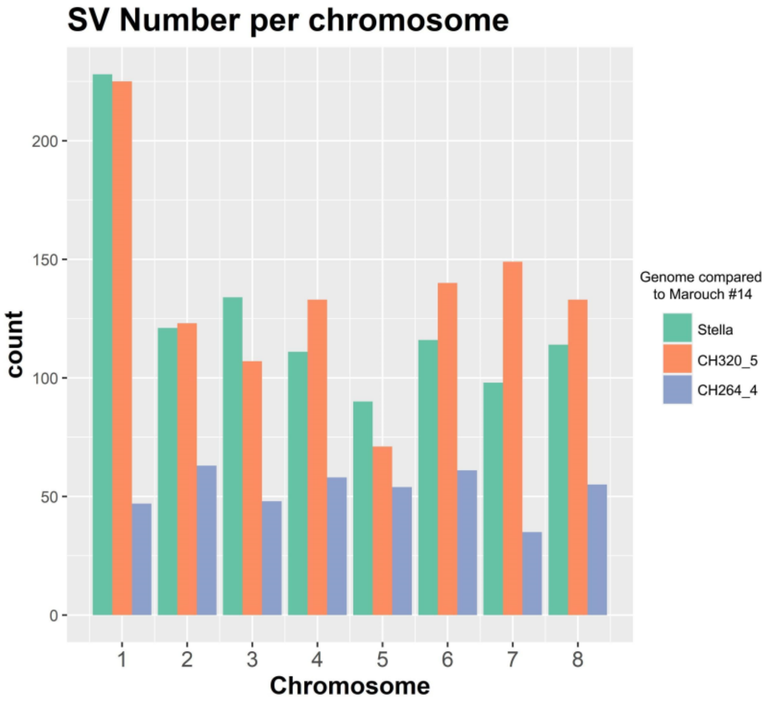

Supplementary Figure 11. Structural variant number per chromosome for cv. Stella, CH320.5 and CH264.4 genomes compared with the Marouch #14 genome.

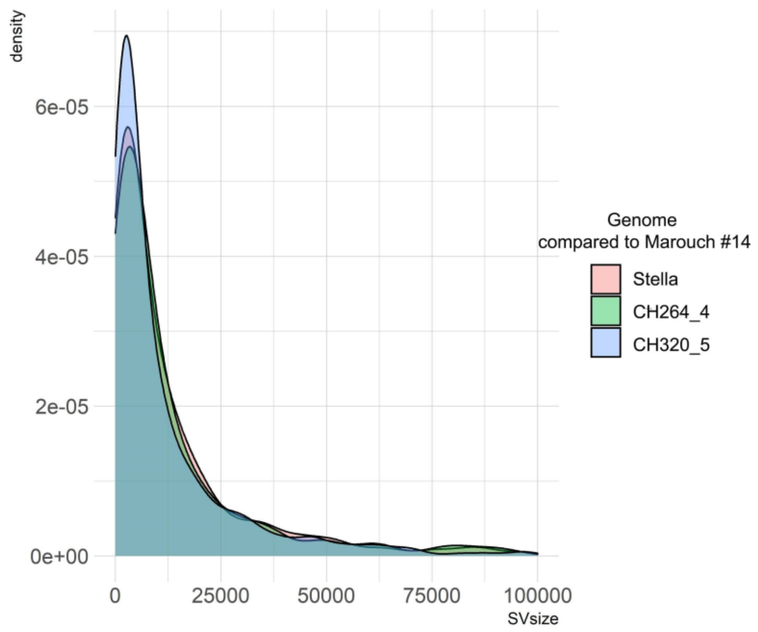

Supplementary Figure 12. Variant size density in cv. Stella, CH320.5 and CH264.4 genomes, in comparison with Marouch #14.

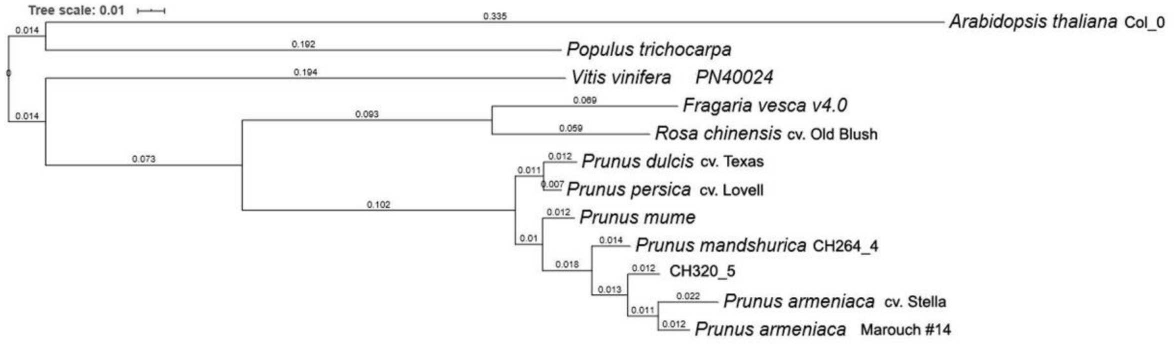

Supplementary Figure 13. PhyML species tree obtained from the concatenation of 298 single-gene orthologs.

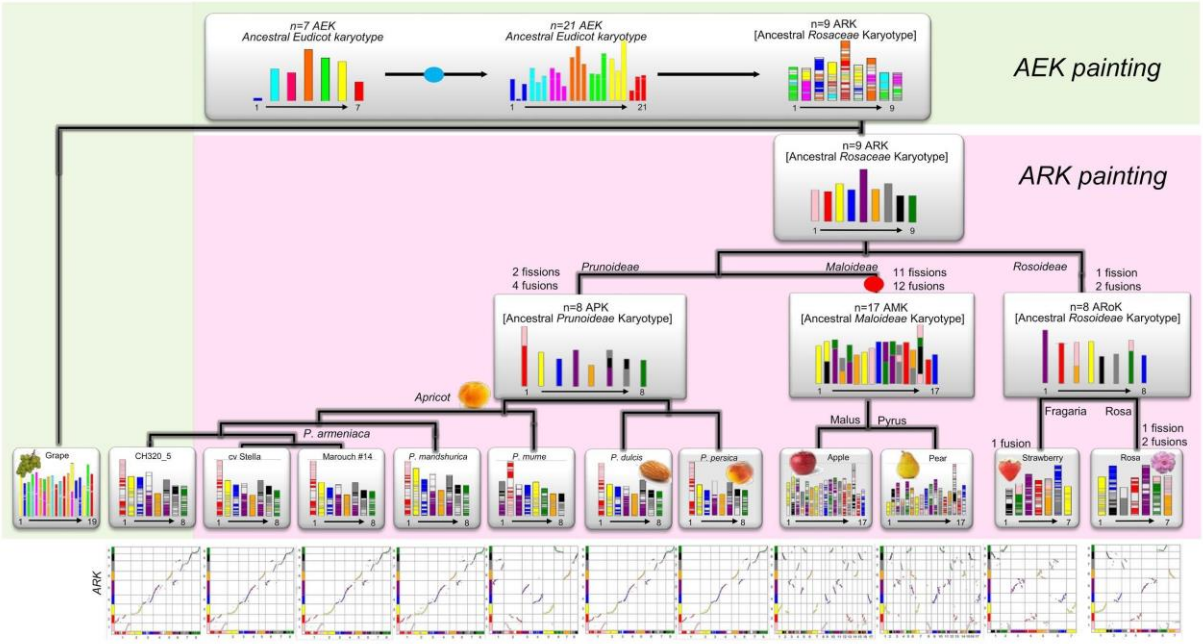

Supplementary Figure 14. Rosaceae genome arrangement evolutionary history.

Evolutionary scenario of the modern Rosaceae (peach, apple, pear, almond, strawberry, rosa and apricots) and grape as an outgroup from the ancestral Eudicot Karyotype (AEK), ancestral Rosaceae karyotype (ARK), ancestral Prunoideae karyotype (APK), ancestral Maloideae karyotype (AMK) and ancestral Rosoideae karyotype (ARoK). The modern genomes are illustrated at the bottom with different colours reflecting the origin from the nine ancestral chromosomes from ARK. Polyploidization events are shown with red (duplication) and blue (triplication) dots on the tree branches, along with the rearrangement shuffling events (fusions and fissions). Complete dot-plot based deconvolution into nine reconstructed conserved ancestral regions CARs (dot-plot y-axis in nine colours) of the observed synteny (dot-plot diagonals) between ARK (dot-plot y-axis) and *Prunus sibirica* CH320_5, *Prunus armeniaca* (Marouch #14, cv. Stella), *Prunus mandshurica* CH264_4, *Prunus mume*, *Prunus dulcis*, rose, peach, apple, pear and strawberry (x-axis).

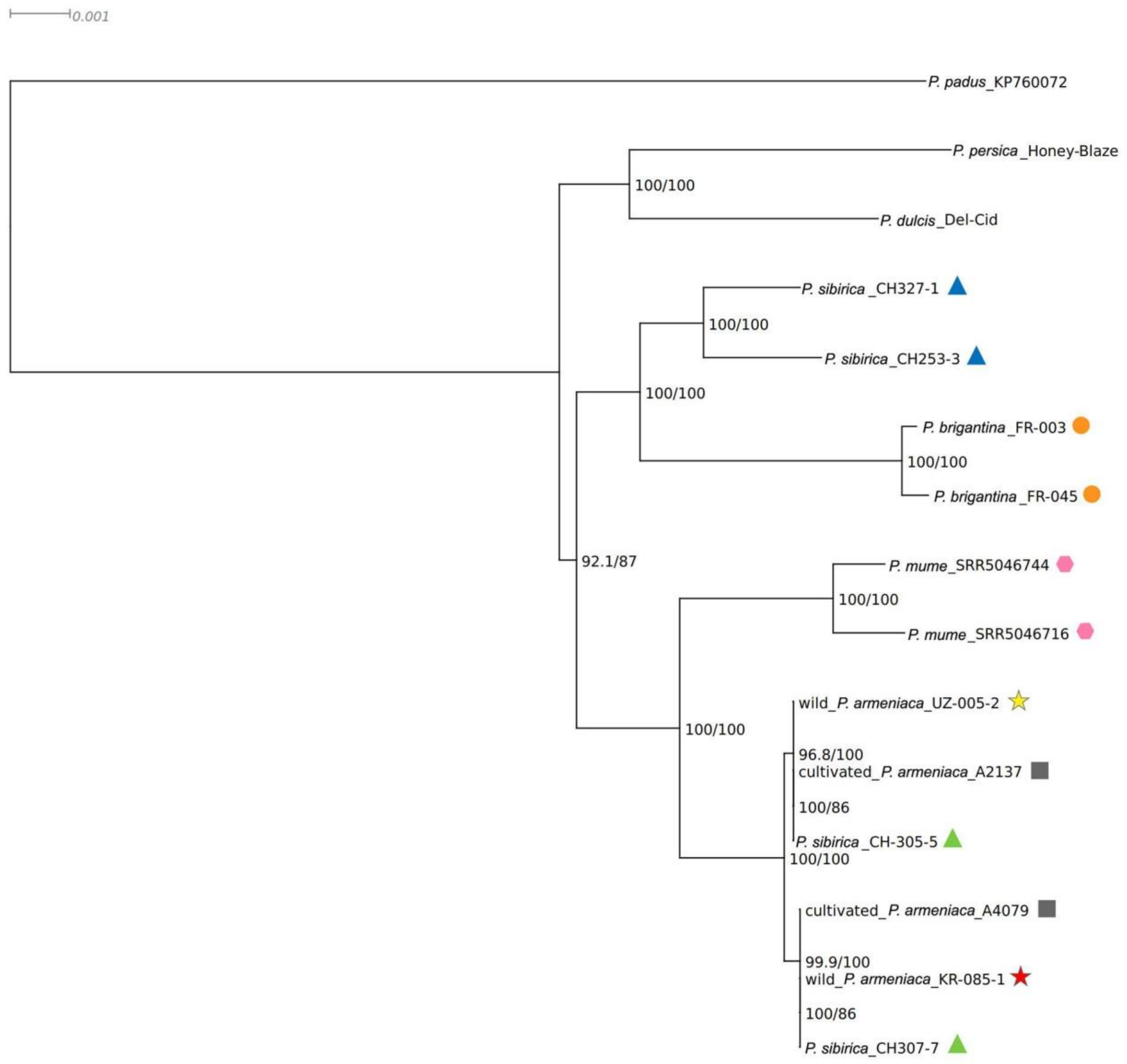

Supplementary Figure 15. Maximum-likelihood phylogenetic tree inferred using IQ-TREE 1.6 from a set of 2,132 variant sites from 15 *Prunus chloroplast* genomes.

Two to four reconstructed chloroplast genomes per species, representing the cpDNA diversity of wild and cultivated *P. armeniaca, P. sibirica, P. mume* and *P. brigantina* were used to construct a Prunus maximum likelihood tree with IQ-TREE 1.6[69], with ascertainment bias correction, nonparametric bootstrap (1,000 replicates) and Shimodaira–Hasegawa likelihood ratio test SH-aLRT (1,000 replicates). The cpDNA assembly of Chinese cherry *P. padus* (KP760072) was included as an outgroup. The colors and signs correspond to the ones depicted in Figure 1a and 1b. Orange circles: *P. brigantina*; pink circles: *P. mume*; grey rectangles: European *P. armeniaca* cultivars; red stars: wild Southern Central Asian *P. armeniaca* (S_Par); yellow stars: wild Northern Central Asian *P. armeniaca* (N_Par); blue triangles: wild Northern Eastern Chinese *P. sibirica* (NE_Psib); green triangles: wild Western Chinese *P. sibirica* (NW_Psib).

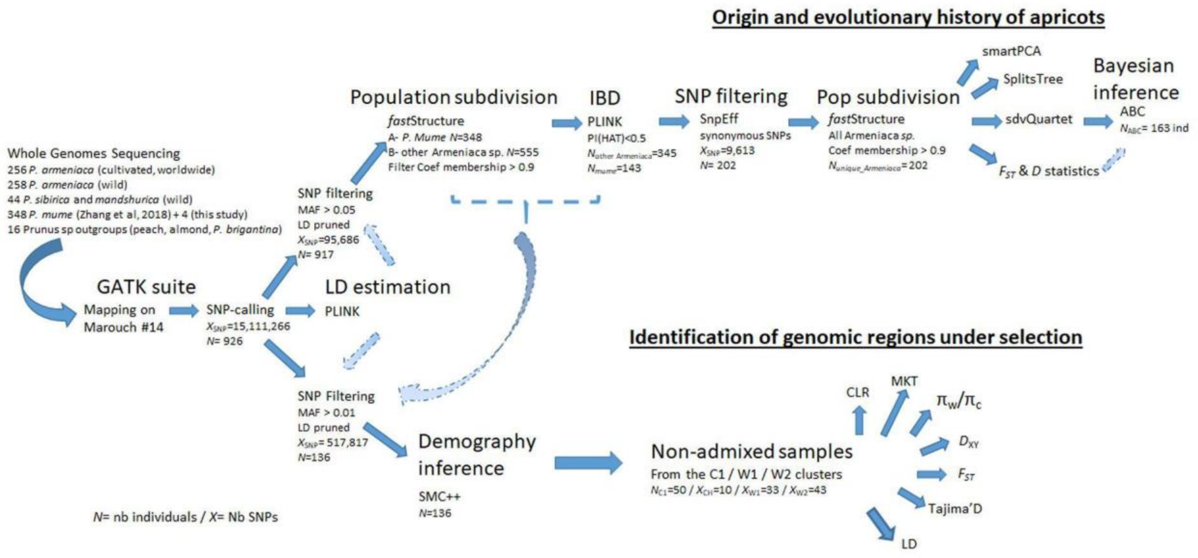

Supplementary Figure 16. Workflow of population genomic analyses.

X, number of SNPs; *N*, number of individuals used in the analysis. All genomes under study are presented in Supplementary Data 1.

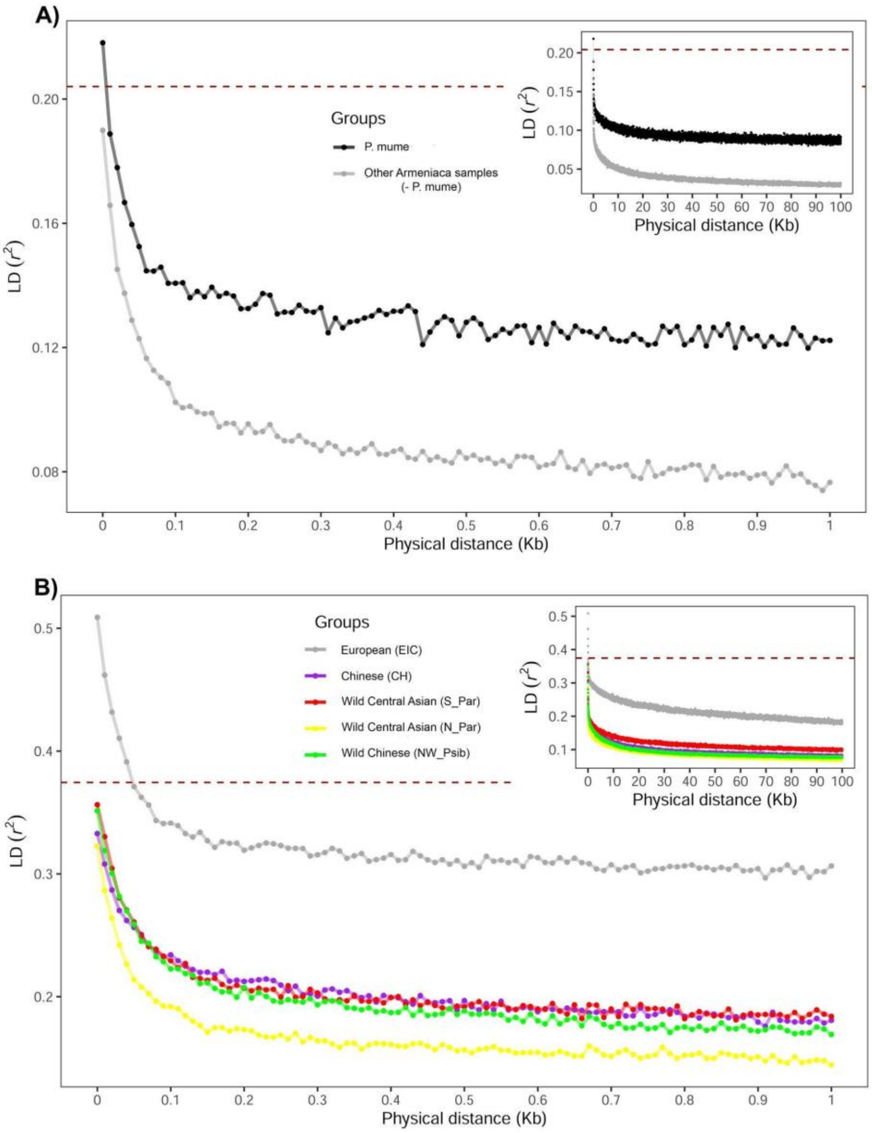

Supplementary Figure 17. Linkage disequilibrium decay.

Linkage disequilibrium (LD) was measured by the squared correlation coefficients (r2) between all pairs of SNPs. (A) LD decay estimated for 348 *P. mume* samples and the 555 genomes from other Armeniaca species. The inner plot displays a higher resolution of LD in pairwise distances of < 1Kb. The red dotted lines in both the inner plot and the main chart refer to the 0.204 threshold. Black line, *Prunus mume;* grey line, other Armeniaca species. (B) LD decay estimated for the two cultivated groups, European and Chinese apricots, and for the two Central Asian *P. armeniaca* natural populations (N_Par and S_Par, corresponding to W2 and W1 respectively). The red dotted line refers to the 0.374 threshold. Grey line, European *P. armeniaca* cultivars; purple line, Chinese *P. armeniaca* cultivars and landraces; red line: wild Southern Central Asian *P. armeniaca* (S_Par); yellow line: wild Northern Central Asian *P. armeniaca* (N_Par); green line: wild Western Chinese *P. sibirica* (NW_Psib).

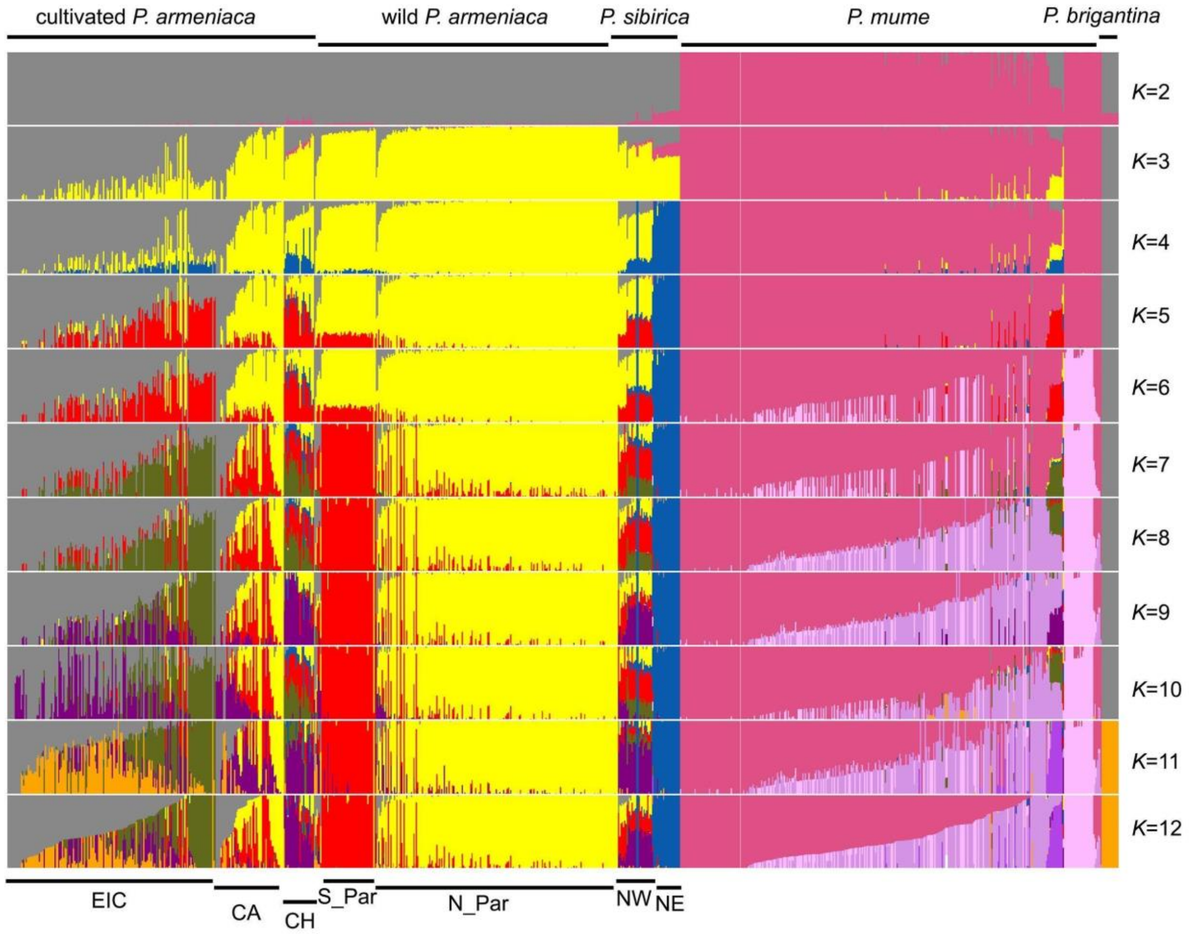

Supplementary Figure 18. Population structure inferred with fastSTRUCTURE from *K*=2 to *K*=12 for the entire Armeniaca dataset (*N*=917, 95,686 SNPs).

For marginal likelihood, see Supplementary Figure 21 A.

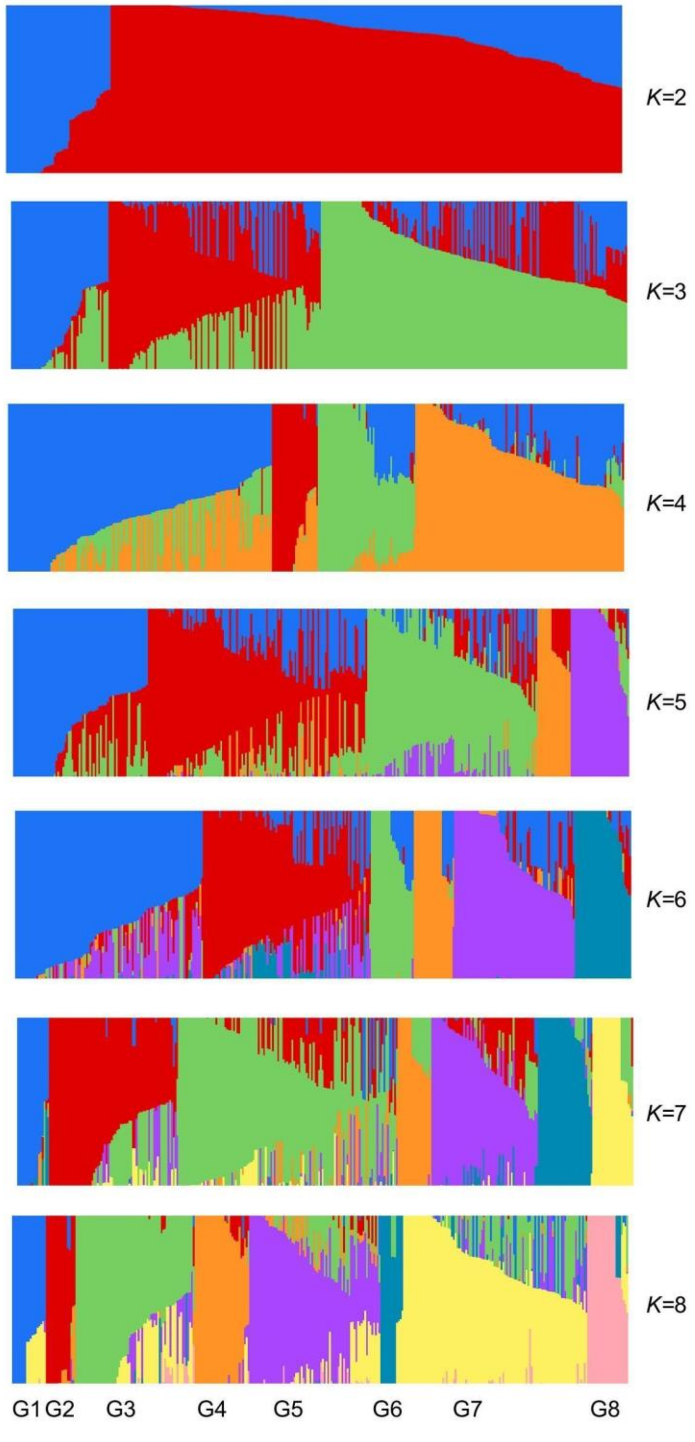

Supplementary Figure 19. Population structure inferred with fastSTRUCTURE from *K*=2 to *K*=8 for the *Prunus mume* dataset (*N*=348, 95,686 SNPs).

For marginal likelihood, see Supplementary Figure 21 B.

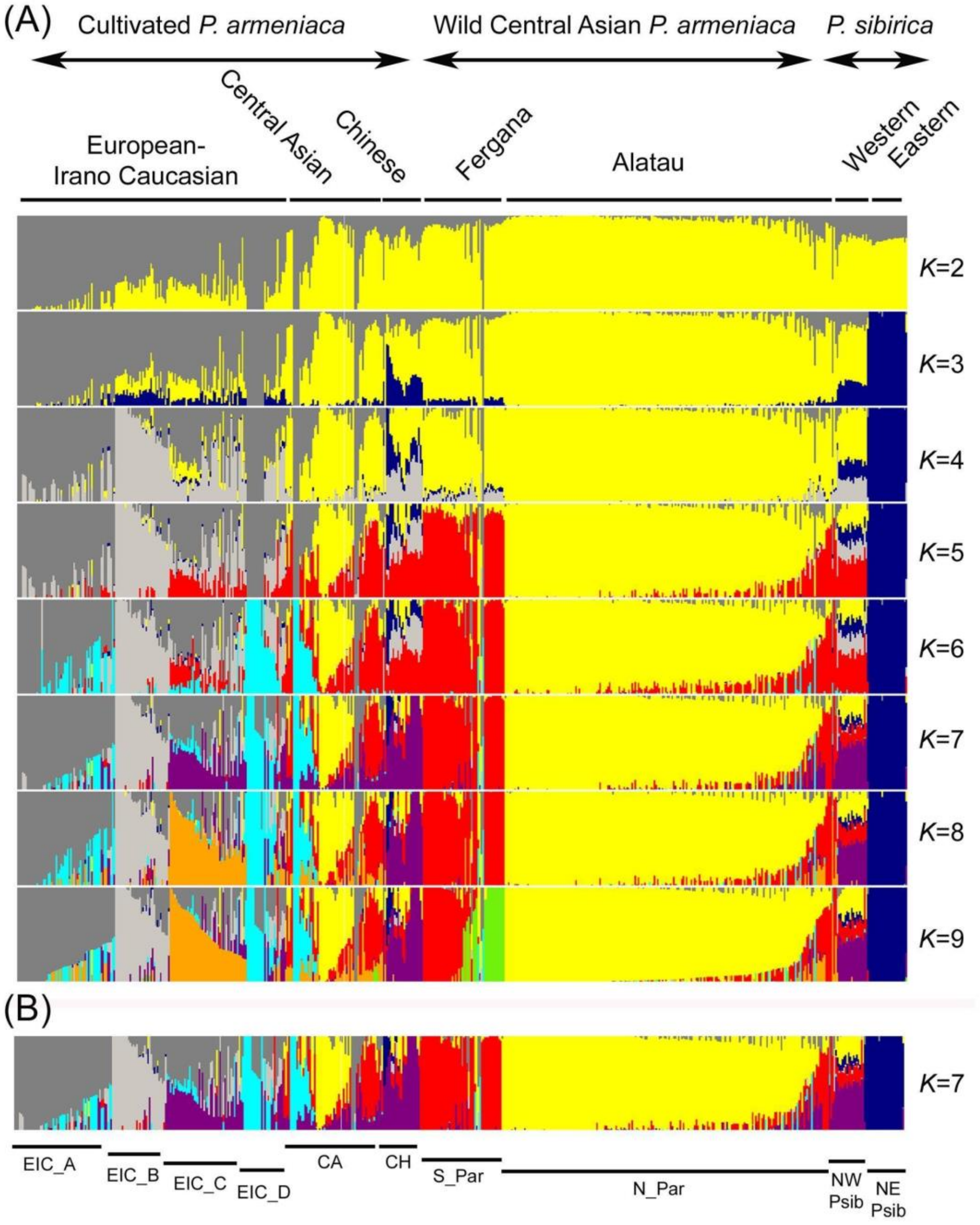

Supplementary Figure 20. Population structure inferred with fastSTRUCTURE from *K*=2 to *K*=8 for the Armeniaca restricted dataset.

Samples of *Prunus mume* were excluded from this FastStructure analysis. For marginal likelihood, see Supplementary Figure 21 C. The most likely *K* value was for *K*=7 (B) EIC, European *P. armeniaca* cultivars; CA, Central Asian *P. armeniaca* cultivars and landraces; CH, Chinese *P. armeniaca* cultivars and landraces; S_Par, wild Southern Central Asian *P. armeniaca*; N_Par, wild Northern Central Asian *P. armeniaca*; NW_Psib, wild Western Chinese *P. sibirica;* NE_Psib, wild Western Chinese *P. sibirica*. *N*=555 individuals, 95,686 SNPs.

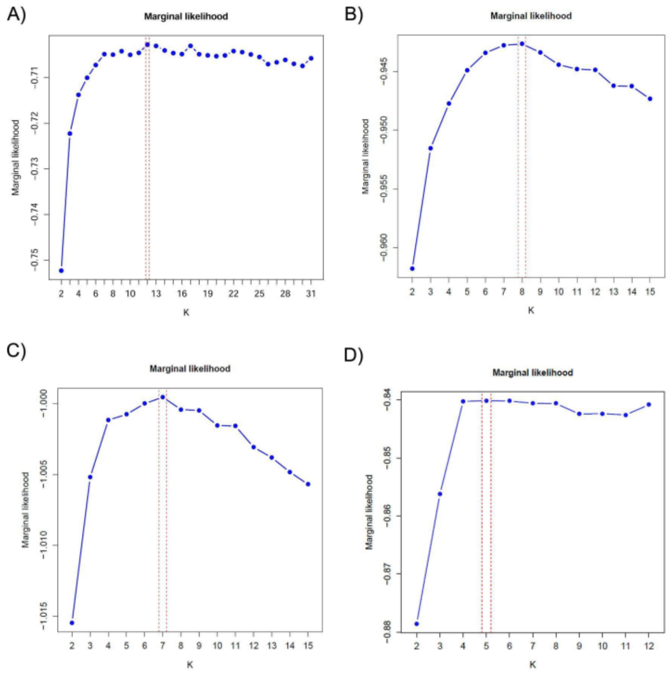

Supplementary Figure 21. Marginal likelihood obtained for the fastSTRUCTURE analyses. The different panels are for the following data: A) the entire Armeniaca dataset (*N*=917), B) the *P. mume* dataset (*N*=348), C) the 'other Armeniaca' dataset (*N*=555), D) the 202 unique accessions**.**

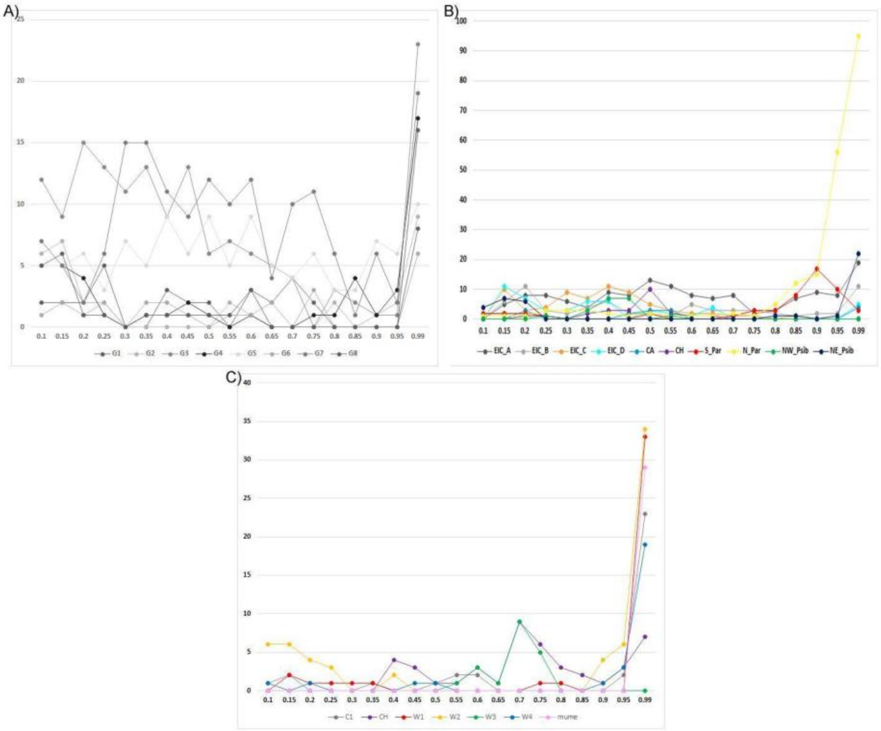

Supplementary Figure 22. The distribution of membership coefficient inferred with fastSTRUCTURE for the various genetic groups.

The genetic clusters correspond to the ones depicted in Supplementary Figures 19A, 20B and 25C. For B) and C): Grey line, European *P. armeniaca* cultivars; purple line, Chinese *P. armeniaca* cultivars and landraces; light blue line, Central Asian *P. armeniaca* landraces; pink line, *P. mume* landraces; red line: wild Southern Central Asian *P. armeniaca* (S_Par); yellow line: wild Northern Central Asian *P. armeniaca* (N_Par); green line: wild Western Chinese *P. sibirica* (NW_Psib); dark blue line: wild Eastern Chinese *P. sibirica* (NE_Psib).

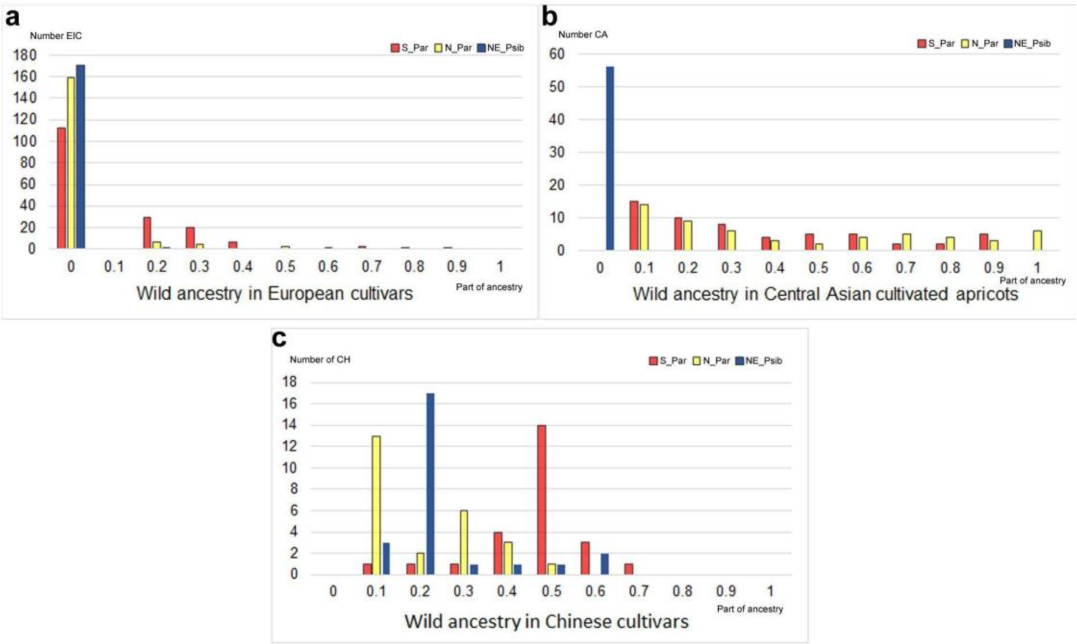

Supplementary Figure 23. Wild Armeniaca ancestry into the cultivated apricot gene pools inferred from fastSTRUCTURE analyses.

The histogram shows the proportion of cultivated apricot accessions with varying proportions of wild Armeniaca (*P. armeniaca* Southern and Northern populations; North Eastern *P. sibirica*) ancestry. Red bars: wild Southern Central Asian *P. armeniaca* (S_Par); yellow bars: wild Northern Central Asian *P. armeniaca* (N_Par); dark blue bars: wild Eastern Chinese *P. sibirica* (NE_Psib). N=555 individuals; 95,686 SNPs, Supplementary Figure 20.

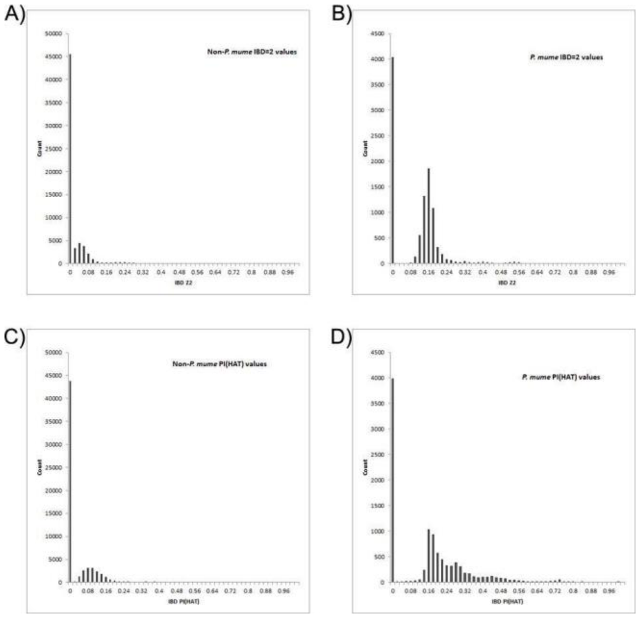

Supplementary Figure 24. Summary statistics of identity by descent values calculated from Armeniaca accessions.

A-B. Histograms of identity by descent (IBD) Z2 values from pairwise comparisons among *P. mume* (B) and the other Armeniaca (A) accessions (*P. mume* excluded). Five *P. mume* accessions with 0.950 (effectively genetically identical) were found in this study (SRR5046580, SRR5046581, SRR5046582, SRR5046626, SRR5046664). No other Armeniaca cultivars showed an IBD Z2 value ≥0.95. C-D. Histogram of PI (HAT) values from pairwise comparisons among *P. mume* (D) and the other Armeniaca (C) accessions.

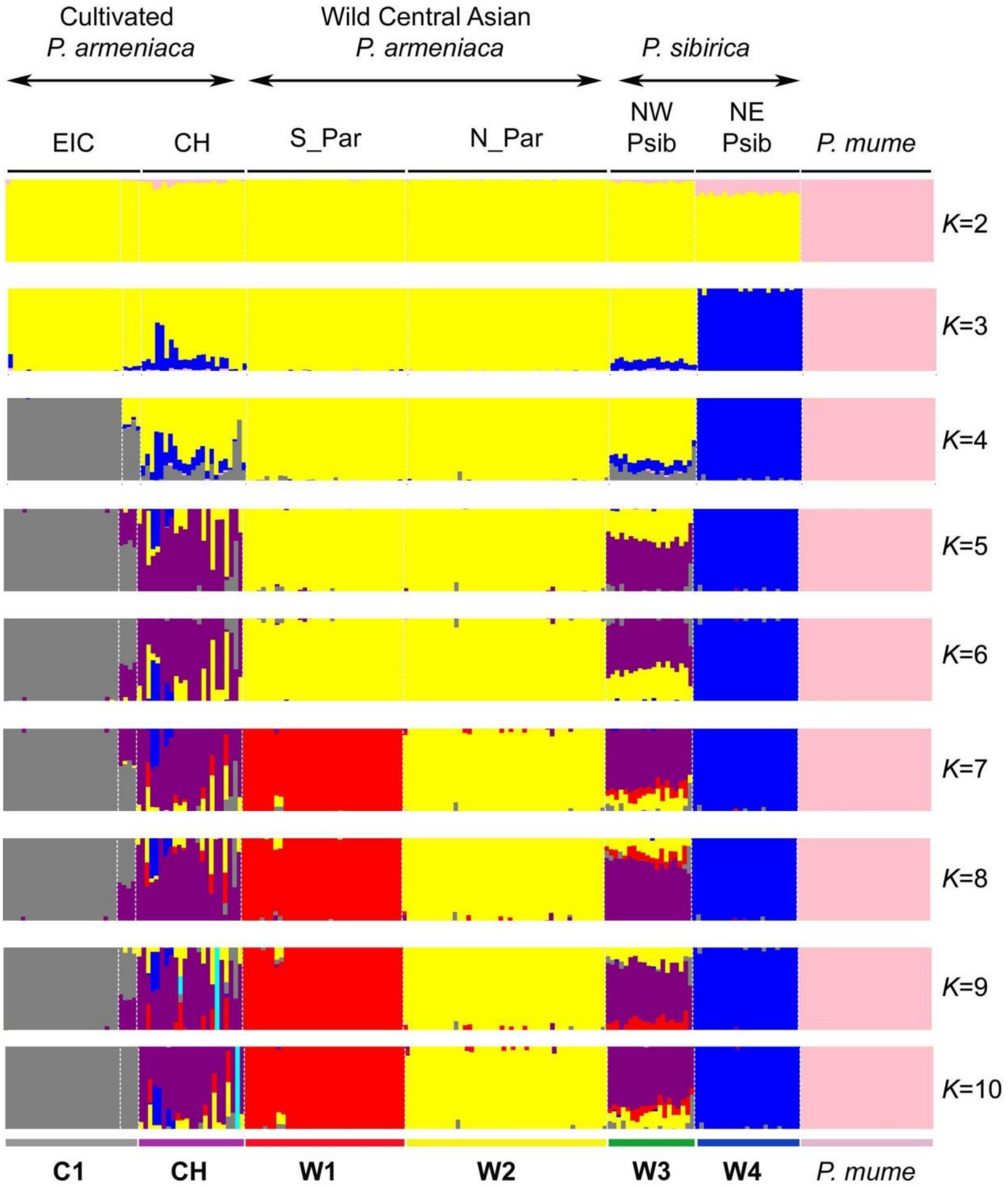

Supplementary Figure 25. Population structure inferred with fastSTRUCTURE from *K*=2 to *K*=10 for the 202 Armeniaca unique accessions using 9,613 unlinked synonymous SNPs.

As in our previous study-[3], C1 (grey) and CH (purple) represent cultivated European and Chinese apricots (*P. armeniaca*), respectively, W1 (red) and W2 (yellow) represent wild Central Asian *P. armeniaca*, W4 represent the wild Chinese North Eastern *Prunus sibirica* (dark blue). This color scheme is later used throughout the next analyses. For marginal likelihood, see above, Supplementary Figure 21d).

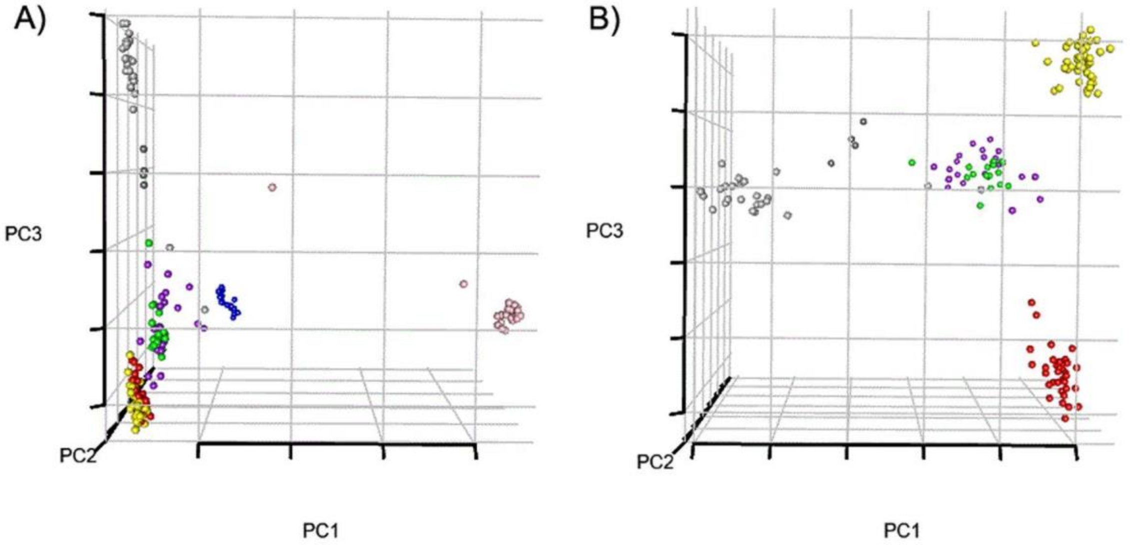

Supplementary Figure 26. Principal component analysis on different apricot genome datasets.

Principal component analysis for the 202 unique apricot accessions a) with and b) without *Prunus mume* and the blue wild *Prunus sibirica* samples (W4).The same color scheme as displayed below in fastSTRUCTURE bar plots (Supplementary Figure 25) is used here. 9,613 SNPs were used in SMARTpca utility of the EIGENSOFT software version 7.2.1[83]. Pink circles: *P. mume*; grey circles: European *P. armeniaca* cultivars and landraces; purple circles: Chinese *P. armeniaca* landraces; red circles: wild Southern Central Asian *P. armeniaca* (S_Par); yellow circles: wild Northern Central Asian *P. armeniaca* (N_Par); dark blue circles: wild Northern Eastern Chinese *P. sibirica* (NE_Psib); green circles: wild Western Chinese *P. sibirica* (NW_Psib).

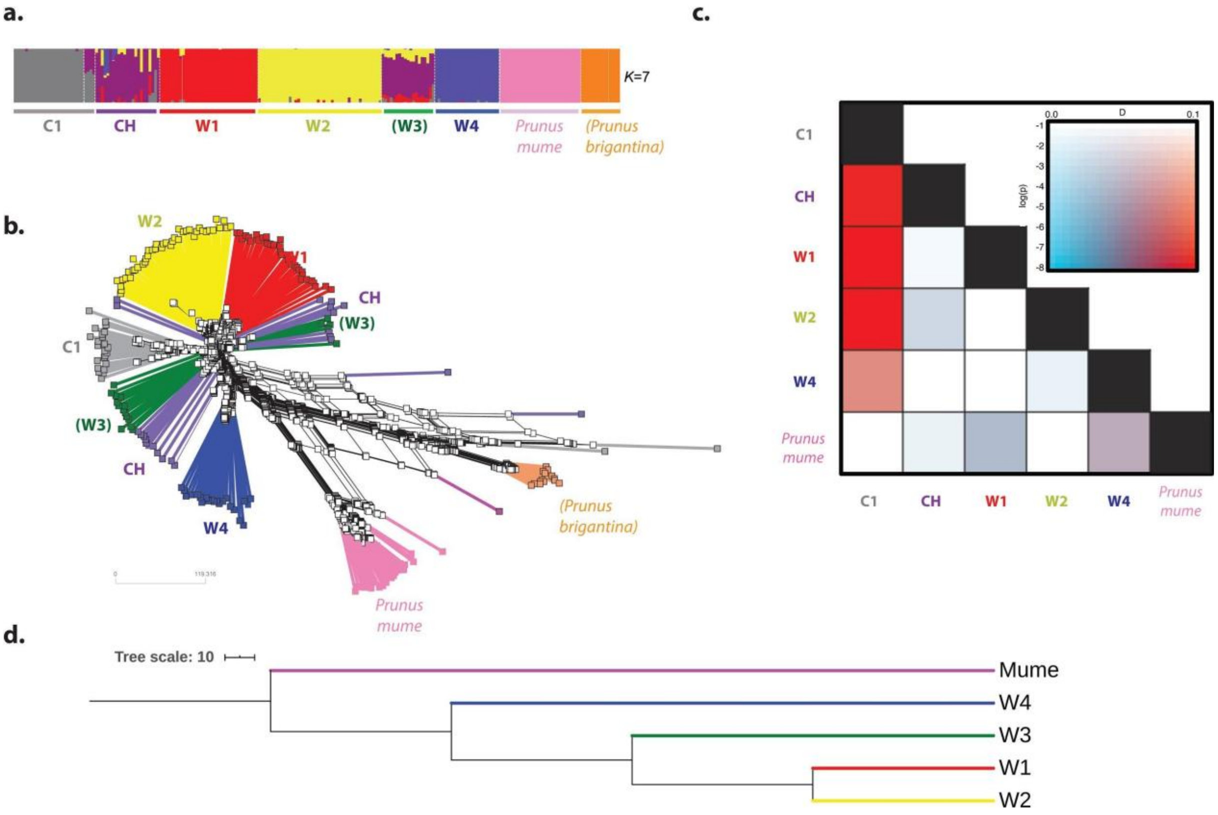

Supplementary Figure 27. Population structure, differentiation, occurrence of gene flow, and genetic relationships among populations used for random forest approximate Bayesian computation to infer the apricot evolutionary history.

a. fastSTRUCTURE barplot for *K*=7 for the 202 unique Armeniaca accessions (9,613 SNPs), in brackets the populations removed for approximate Bayesian computation (ABC-RF). Note that admixed individuals with membership <0.90 to any cluster were also removed. **b**. Splitstree of the 202 unique Armeniaca accessions coloured according to the genetic groups detected for *K*=7 with fastSTRUCTURE **c**. *D-statistic* estimates among the six populations kept for ABC-RF inferences (C1, CH, W1, W2, W4, *Prunus mume*), with the associated *p* values: the lowest the *p* values are, the reddish the color. **d**. SDVquartet tree inferred for the four wild apricots and *P. mume* genetic cluster, excluding *Prunus brigantina* and the Chinese and European cultivated apricots. Pink color: *P. mume*; orange, *P. brigantina*; grey: European *P. armeniaca* cultivars (C1); purple: Chinese *P. armeniaca* landraces (CH); red: wild Southern Central Asian *P. armeniaca* (S_Par, W1); yellow: wild Northern Central Asian *P. armeniaca* (N_Par, W2); green: wild Western Chinese *P. sibirica* (NW_Psib, W3); dark blue: wild Northern Eastern Chinese *P. sibirica* (NE_Psib, W4).

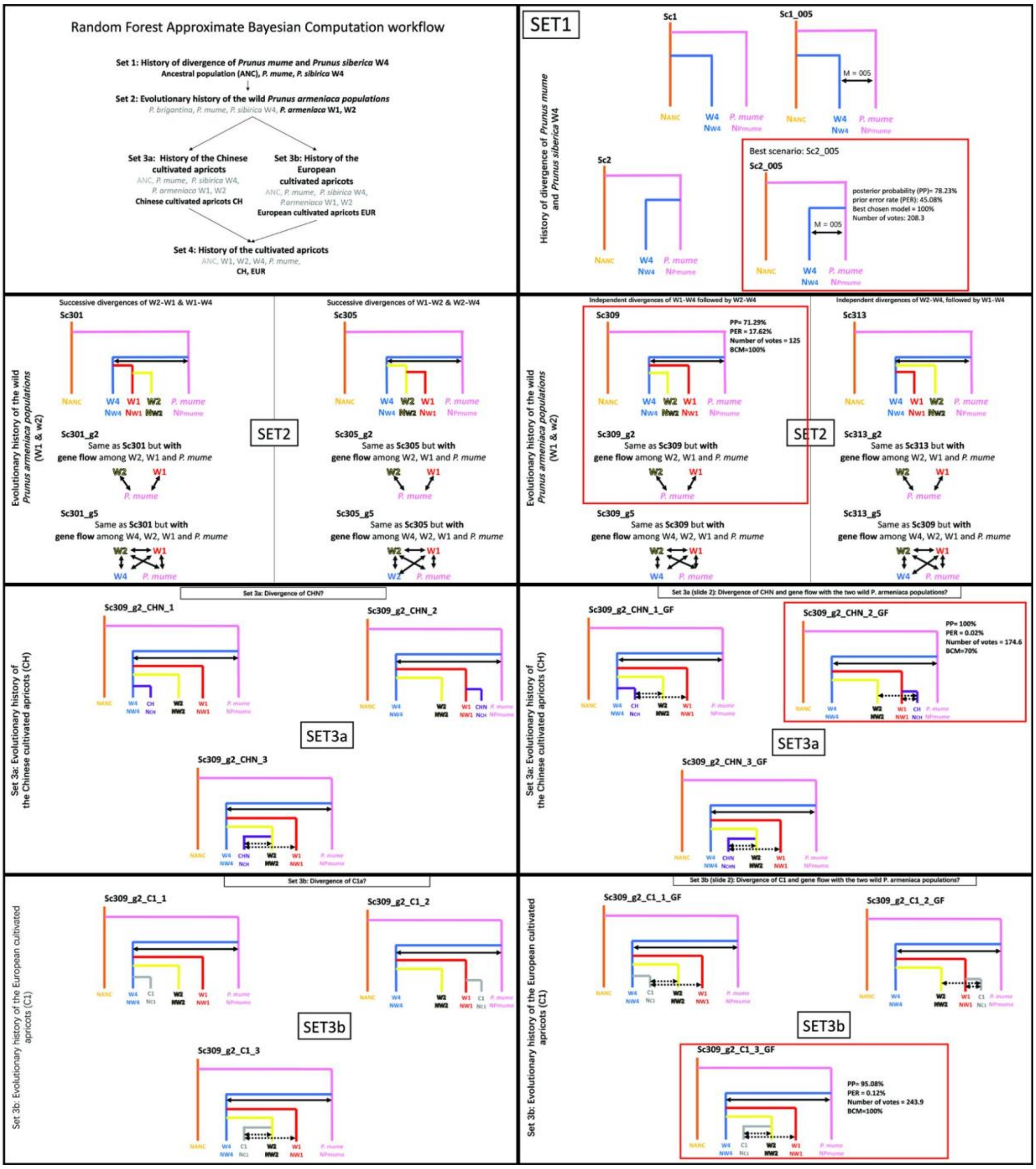

Supplementary Figure 28. The scenarios of divergence history among Armeniaca species (sets 1 and 2) and of domestication (sets 3 and 4) tested by random forest approximate Bayesian computation. The whole pipeline is first described, and then the ABC different sets (1 to 4), with no gene flow between populations, or gene flow among the populations. Populations are the ones inferred for K=7 with fastSTRUCTURE. Posterior probabilities (PP), prior error rate (PE), and number and proportions (BCM) of votes are presented for the most likely scenario at each set. N_X: Effective population size of population X; $m_{X\text{-}Y}$: bidirectional arrows representing gene flow between populations X and Y; $T_{X\text{-}Y}$: divergence time between populations X and Y.

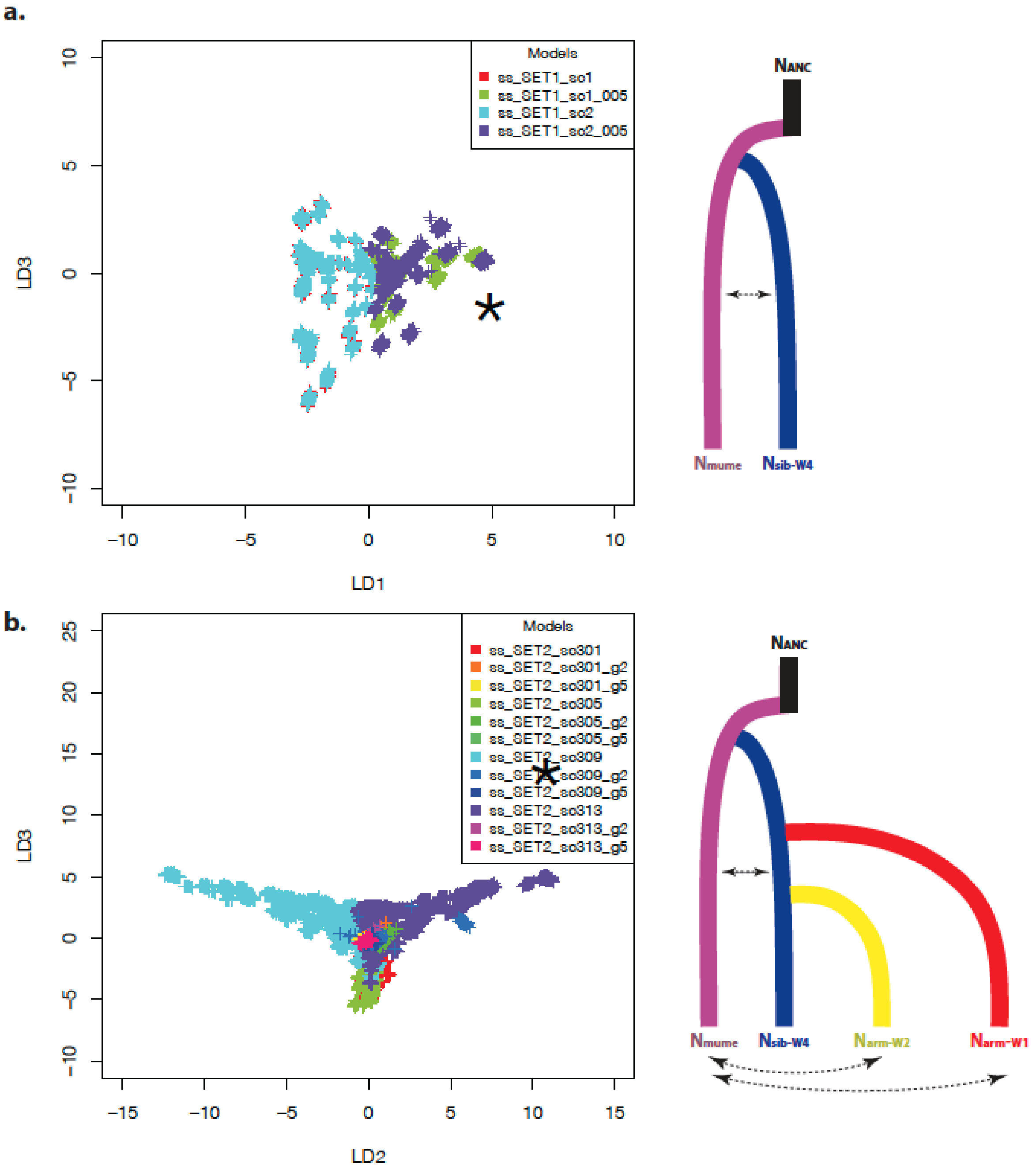

Supplementary Figure 29. Linear discriminant analysis explaining most of the variance for the two first random forest approximate Bayesian computation sets to infer wild apricot evolutionary history, and the associated most likely chosen scenario on the right side of the linear discriminant analysis.

a. Random forest approximate Bayesian computation (ABC-RF) set 1 to infer the evolutionary history of *Prunus mume* (pink) and the wild *Prunus sibirica* (W4 genetic cluster, dark blue). b. ABC-RF set 2 to infer the evolutionary history of the two *Prunus armeniaca* genetic groups (W1 and W2, red and yellow respectively).

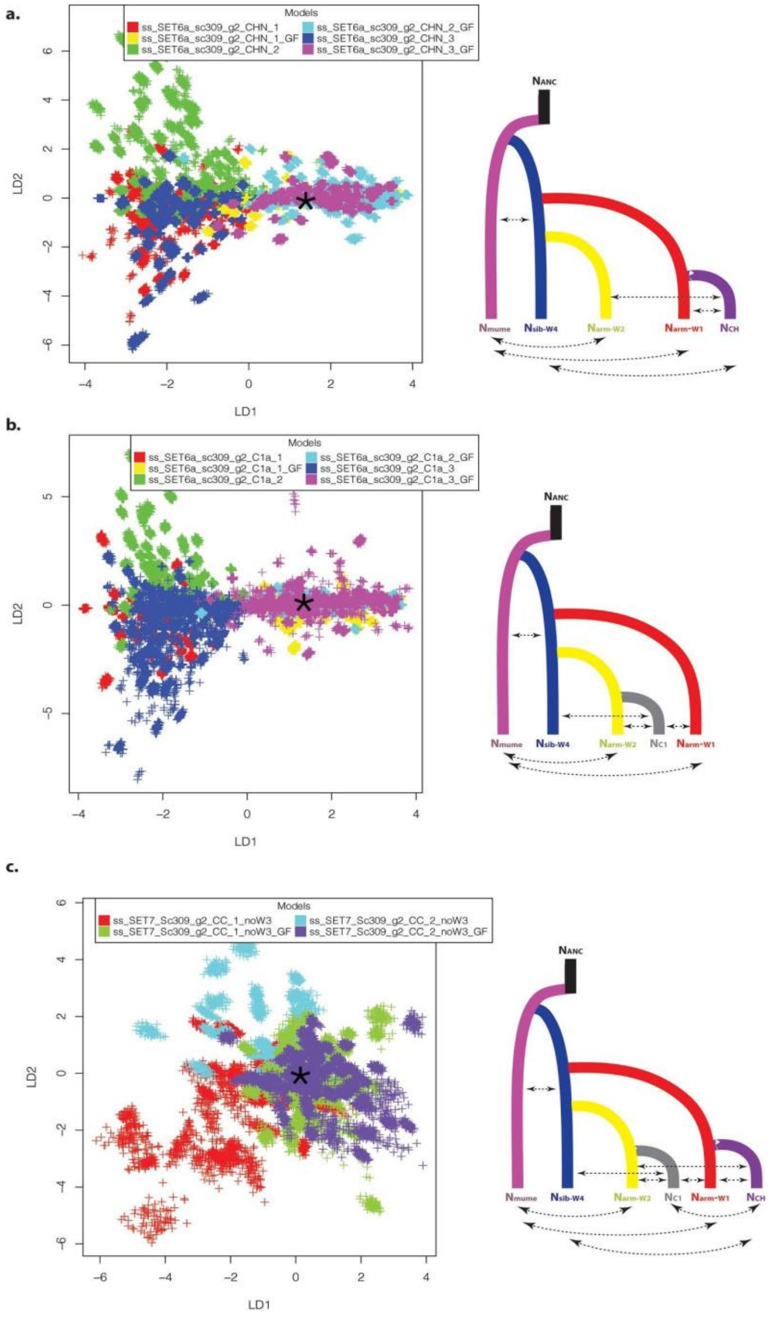

Supplementary Figure 30. Linear discriminant analysis explaining most of the variance for the two first random forest approximate Bayesian computation sets to infer apricot domestication history, and the associated most likely chosen scenario on the right side of the linear discriminant analysis.

a. Random forest approximate Bayesian computation (ABC-RF) step 3a to infer the domestication history of the Chinese cultivated apricots. b. ABC-RF step 3b to infer the evolutionary history of the European cultivated apricots c. ABC-RF step 4 to infer the relative timing of domestication of the Chinese and European cultivated apricots, and the extent of gene flow among wild and cultivated apricots (final scenario depicted in Figure 5). Color codes for the inferred trees displayed on the right: pink, *P. mume*; grey, European *P. armeniaca* cultivars (C1); purple, Chinese *P. armeniaca* landraces (CH); red, wild Southern Central Asian *P. armeniaca* (S_Par, W1); yellow, wild Northern Central Asian *P. armeniaca* (N_Par, W2); dark blue, wild Northern Eastern Chinese *P. sibirica* (NE_Psib, W4).

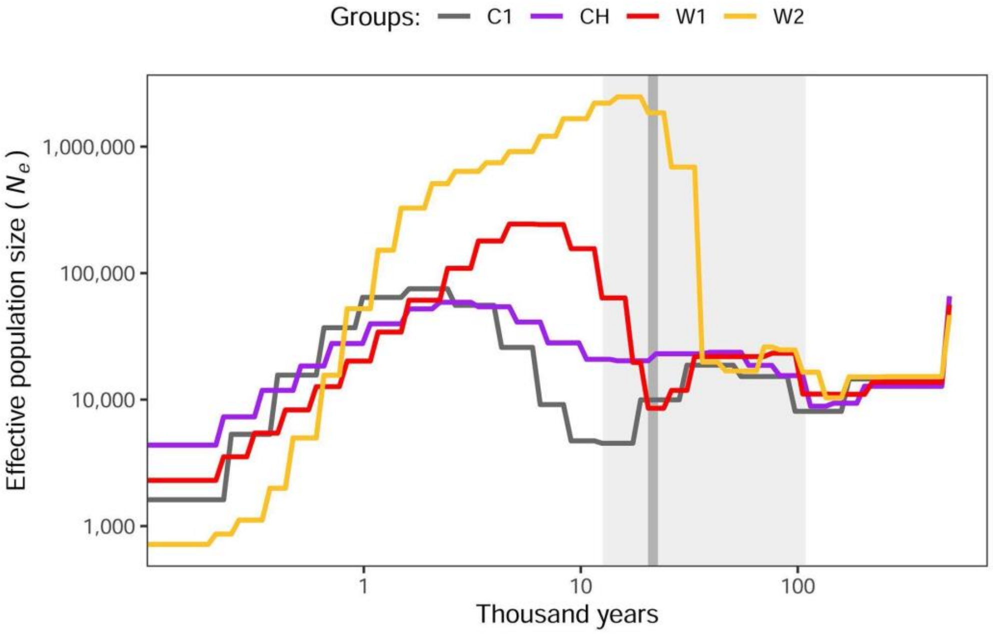

Supplementary Figure 31. Historical effective population size for *Prunus armeniaca* beginning from 0.5 million years ago to present.

Stairway plots showing that the apricot natural and cultivated populations have undergone bottlenecks mainly during the last period, following the Last Glaciation Moment (LGM; dark grey vertical line). Red line, wild Southern Central Asian *P. armeniaca* (S_Par, W1); yellow, wild Northern Central Asian *P. armeniaca* (N_Par, W2); grey line, European *P. armeniaca* cultivars (C1); purple line, Chinese *P. armeniaca* landraces (CH); Ne: effective population size.

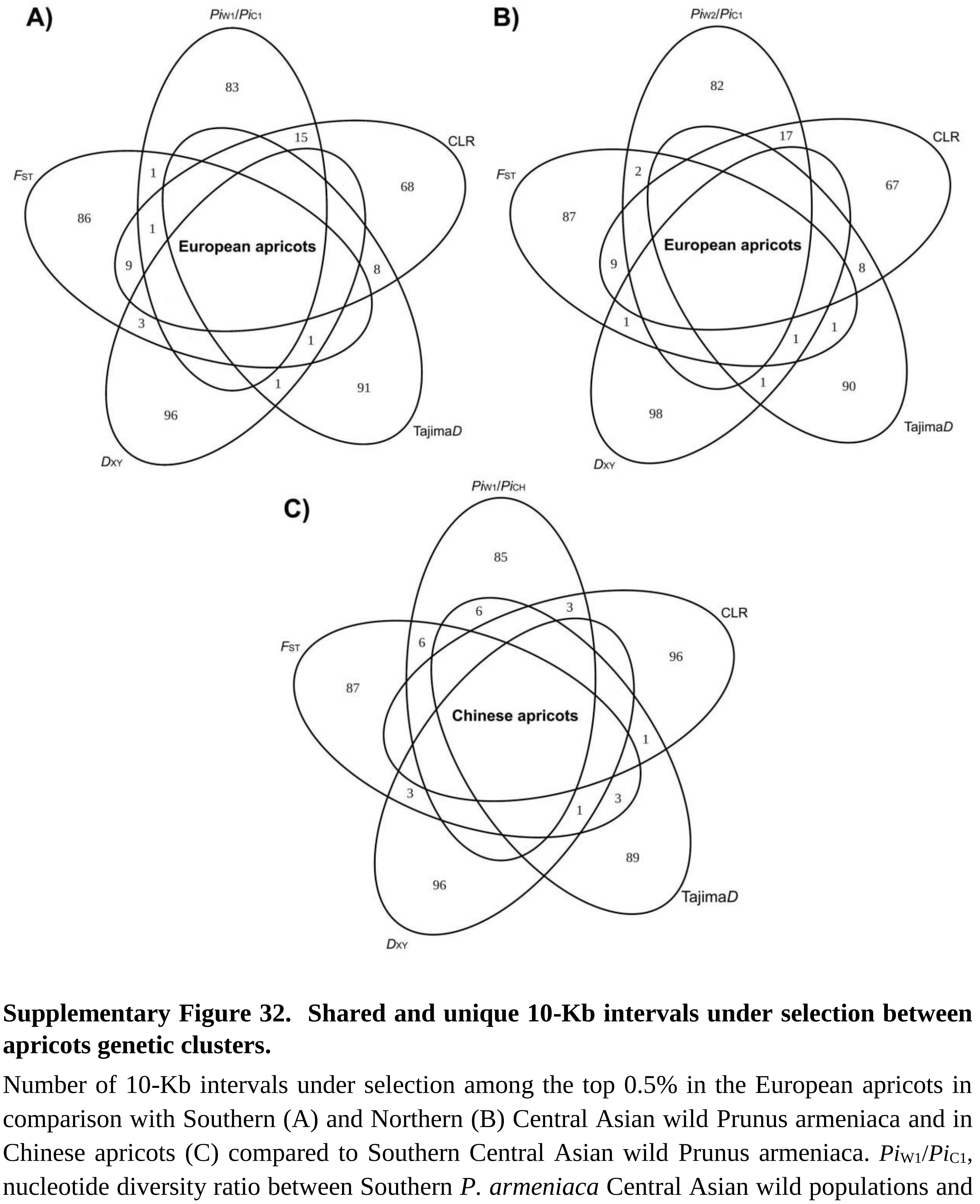

Supplementary Figure 32. Shared and unique 10-Kb intervals under selection between apricots genetic clusters.

Number of 10-Kb intervals under selection among the top 0.5% in the European apricots in comparison with Southern (A) and Northern (B) Central Asian wild Prunus armeniaca and in Chinese apricots (C) compared to Southern Central Asian wild Prunus armeniaca. Pi_{W1}/Pi_{C1}, nucleotide diversity ratio between Southern *P. armeniaca* Central Asian wild populations and European cultivated apricots; Pi_{W2}/Pi_{C1}, nucleotide diversity ratio between Northern *P. armeniaca* Central Asian wild populations and European cultivated apricots; Pi_{W1}/Pi_{CH}, nucleotide diversity ratio between Southern *P. armeniaca* Central Asian wild populations and Chinese cultivated apricots[102]; F_{ST}, Fixation or differentiation index[126]; D_{XY}, pairwise nucleotide substitution or absolute measure of differentiation[102]; Tajima *D*, neutrality index[103]; CLR, composite likelihood ratio[101]. Numbers indicate 10Kb intervals that are unique to the test or shared between at least two tests.

Supplementary references

1. Zhang, Q. *et al*. The genome of *Prunus mume*. *Nature Communications* **3**, 1318 (2012).
2. Zhang, Q. *et al*. The genetic architecture of floral traits in the woody plant *Prunus mume. Nature Communications* **9**, 1702 (2018).
3. Liu, S. *et al.* The complex evolutionary history of apricots: Species divergence, gene flow and multiple domestication events. *Molecular Ecology* **28**, 5299-5314 (2019).
4. Decroocq, S. *et al.* New insights into the history of domesticated and wild apricots and its contribution to Plum pox virus resistance. *Molecular Ecology* **25**, 4712-4729 (2016).
5. Liu, S. *et al.* Genetic diversity and population structure analyses in the Alpine plum (*Prunus brigantina* Vill.) confirm its affiliation to the Armeniaca section. *Tree Genetics & Genomes* **17**, 2 (2021).
6. Zhebentyayeva, T., Ledbetter, C., Burgos, L. & Llácer, G. Apricot. in Fruit Breeding 415-458 (Springer, Boston, MA, 2012).
7. Mariette, S. *et al.* Genome-wide association links candidate genes to resistance to Plum Pox Virus in apricot *(Prunus armeniaca*). *New Phytologist* **209**, 773-784 (2016).
8. Doyle, J.J. & Doyle, J.L. A rapid DNA isolation procedure for small quantities of fresh leaf tissue. *Phytochemical Bulletin* **19**, 11-15 (1987).
9. Chang, S., Puryear, J. & Cairney, J. A simple and efficient method for isolating RNA from pine trees. *Plant Molecular Biology Reporter* **11**, 113-116 (1993).
10. Alberti, A. *et al.* Viral to metazoan marine plankton nucleotide sequences from the Tara Oceans expedition. *Scientific Data* **4**, 170093 (2017).
11. Chin, C.-S. *et al*. Phased diploid genome assembly with single-molecule real-time sequencing. *Nature Methods* **13**, 1050-1054 (2016).
12. Chin, C.-S. *et al.* Nonhybrid, finished microbial genome assemblies from long-read SMRT sequencing data. *Nature Methods* **10**, 563-569 (2013).
13. Chaisson, M.J. & Tesler, G. Mapping single molecule sequencing reads using basic local alignment with successive refinement (BLASR): application and theory. *BMC Bioinformatics* **13**, 238 (2012).
14. Roach, M.J., Schmidt, S.A. & Borneman, A.R. Purge Haplotigs: allelic contig reassignment for third-gen diploid genome assemblies. *BMC Bioinformatics* **19**, 460 (2018).
15. Li, H. Minimap2: pairwise alignment for nucleotide sequences. Bioinformatics **34**, 3094-3100 (2018).
16. Luo, R. *et al.* SOAPdenovo2: an empirically improved memory-efficient short-read de novo assembler. *GigaScience* **1**, 18 (2012).
17. Vaser, R., Sović, I., Nagarajan, N. & Šikić, M. Fast and accurate *de novo* genome assembly from long uncorrected reads. *Genome Research* **27**, 737-746 (2017).
18. Walker, B.J. *et al.* Pilon: An integrated tool for comprehensive microbial variant detection and genome assembly improvement. *PLoS One* **9**, e112963 (2014).
19. Belser, C. *et al.* Chromosome-scale assemblies of plant genomes using nanopore long reads and optical maps. *Nature Plants* **4**, 879-887 (2018).
20. Istace, B., Belser, C. & Aury, J.-M. BiSCoT: improving large eukaryotic genome assemblies with optical maps. *PeerJ* **8**, e10150-e10150 (2020).
21. Aury, J.-M. & Istace, B. Hapo-G, Haplotype-aware polishing of genome assemblies. *BioRxiv*, 2020.12.14.422624 (2020).

22. Seppey, M., Manni, M. & Zdobnov, E.M. BUSCO: Assessing genome assembly and annotation completeness. *Methods in Molecular Biology* **1962**, 227-245 (2019).
23. Alonge, M. *et al.* RaGOO: fast and accurate reference-guided scaffolding of draft genomes. *Genome Biology* **20**, 224 (2019).
24. Jiang, F. *et al.* The apricot *(Prunus armeniaca* L.) genome elucidates Rosaceae evolution and beta-carotenoid synthesis. *Horticulture Research* **6**, 128 (2019).
25. Campoy, J.A. *et al.* Gamete binning: chromosome-level and haplotype-resolved genome assembly enabled by high-throughput single-cell sequencing of gamete genomes. *Genome Biology* **21**, 306 (2020).
26. Marçais, G. & Kingsford, C. A fast, lock-free approach for efficient parallel counting of occurrences of k-mers. *Bioinformatics* **27**, 764-70 (2011).
27. Ranallo-Benavidez, T.R., Jaron, K.S. & Schatz, M.C. GenomeScope 2.0 and Smudgeplot for reference-free profiling of polyploid genomes. *Nature Communications* **11**, 1432 (2020).
28. Dondini, L. *et al.* Development of a new SSR-based linkage map in apricot and analysis of synteny with existing Prunus maps. *Tree Genetics & Genomes* **3**, 239-249 (2007).
29. Soriano, J.M. *et al.* Identification of simple sequence repeat markers tightly linked to plum pox virus resistance in apricot. *Molecular breeding* **30**, 1017-1026 (2012).
30. Illa, E. *et al.* Linkage map saturation, construction, and comparison in four populations of Prunus. Journal of Horticultural Science and Biotechnology **84**(2009).
31. Marandel, G., Salava, J., Abbott, A., Candresse, T. & Decroocq, V. Quantitative trait loci meta-analysis of Plum pox virus resistance in apricot *(Prunus armeniaca* L.): new insights on the organization and the identification of genomic resistance factors. *Molecular Plant Pathology* **10**, 347-360 (2009).
32. Kuhn, R.M., Haussler, D. & Kent, W.J. The UCSC genome browser and associated tools. *Briefings in Bioinformatics* **14**, 144-161 (2012).
33. Tang, H. *et al.* ALLMAPS: robust scaffold ordering based on multiple maps. *Genome Biology* **16**, 3 (2015).
34. Chen, S., Zhou, Y., Chen, Y. & Gu, J. fastp: an ultra-fast all-in-one FASTQ preprocessor. *Bioinformatics* **34**, i884-i890 (2018).
35. Dobin, A. *et al.* STAR: ultrafast universal RNA-seq aligner. *Bioinformatics* **29**, 15-21 (2013).
36. Grabherr, M.G. *et al.* Full-length transcriptome assembly from RNA-Seq data without a reference genome. *Nature Biotechnology* **29**, 644-652 (2011).
37. Haas, B.J. *et al. De novo* transcript sequence reconstruction from RNA-seq using the Trinity platform for reference generation and analysis. *Nature Protocols* **8**, 1494-1512 (2013).
38. Verde, I. *et al.* The high-quality draft genome of peach *(Prunus persica*) identifies unique patterns of genetic diversity, domestication and genome evolution. *Nature Genetics* **45**, 487-494 (2013).
39. Bruna, T., Hoff, K., Stanke, M., Lomsadze, A. & Borodovsky, M. BRAKER2: Automatic eukaryotic genome annotation with GeneMark-EP+ and AUGUSTUS supported by a protein database *NAR Genomics and Bioinformatics* **3**, lqaa108 (2020).
40. Hoff, K.J., Lange, S., Lomsadze, A., Borodovsky, M. & Stanke, M. BRAKER1: Unsupervised RNA-Seq-based genome annotation with GeneMark-ET and AUGUSTUS. *Bioinformatics* **32**, 767-769 (2015).
41. Bryant, D.M. *et al.* A Tissue-mapped axolotl *de novo* transcriptome enables identification of limb regeneration factors. *Cell Reports* **18**, 762-776 (2017).
42. Altschul, S.F., Gish, W., Miller, W., Myers, E.W. & Lipman, D.J. Basic local alignment search tool. *Journal of Molecular Biology* **215**, 403-410 (1990).

43. Shah, N., Nute, M.G., Warnow, T. & Pop, M. Misunderstood parameter of NCBI BLAST impacts the correctness of bioinformatics workflows. *Bioinformatics* **35**, 1613-1614 (2019).
44. Finn, R.D., Clements, J. & Eddy, S.R. HMMER web server: interactive sequence similarity searching. *Nucleic Acids Research* **39**, W29-W37 (2011).
45. Punta, M. *et al.* The Pfam protein families database. *Nucleic Acids Res*earch **40**, D290-301 (2012).
46. Cabanettes, F. & Klopp, C. D-GENIES: dot plot large genomes in an interactive, efficient and simple way. *PeerJ* **6**, e4958 (2018).
47. Hu, Y. *et al*. OmicCircos: A simple-to-use R package for the circular visualization of multidimensional omics data. *Cancer Informatics* **13**, CIN.S13495 (2014).
48. Emms, D.M. & Kelly, S. OrthoFinder: solving fundamental biases in whole genome comparisons dramatically improves orthogroup inference accuracy. *Genome Biology* **16**, 157 (2015).
49. Emms, D.M. & Kelly, S. OrthoFinder: phylogenetic orthology inference for comparative genomics. *Genome Biology* **20**, 238 (2019).
50. Katoh, K. & Standley, D.M. MAFFT multiple sequence alignment software version 7: improvements in performance and usability. *Molecular Biology and Evolution* **30**, 772-80 (2013).
51. Anisimova, M. & Gascuel, O. Approximate likelihood-ratio test for branches: a fast, accurate, and powerful alternative. *Systematic Biology* **55**, 539-552 (2006).
52. Guindon, S. & Gascuel, O. A simple, fast, and accurate algorithm to estimate large phylogenies by maximum likelihood. *Systematic Biology* **52**, 696-704 (2003).
53. Bouckaert, R. *et al.* BEAST 2.5: An advanced software platform for Bayesian evolutionary analysis. *PLoS Computational Biology* **15**, e1006650 (2019).
54. Drummond, A.J., Suchard, M.A., Xie, D. & Rambaut, A. Bayesian phylogenetics with BEAUti and the BEAST 1.7. *Molecular Biology and Evolution* **29**, 1969-1973 (2012).
55. Heled, J. & Drummond, A.J. Calibrated tree priors for relaxed phylogenetics and divergence time estimation. *Systematic Biology* **61**, 138-149 (2011).
56. Töpel, M., Antonelli, A., Yesson, C. & Eriksen, B. Past climate change and plant evolution in Western North America: a case study in Rosaceae. *PLoS ONE* **7**, e50358 (2012).
57. Tuskan, G.A. *et al*. The genome of black cottonwood, *Populus trichocarpa* (Torr. & Gray). *Science* **313**, 1596-1604 (2006).
58. Rambaut, A., Drummond, A.J., Xie, D., Baele, G. & Suchard, M.A. Posterior Summarization in Bayesian phylogenetics using tracer 1.7. *Systematic Biology* **67**, 901-904 (2018).
59. Pont, C. *et al.* Paleogenomics: reconstruction of plant evolutionary trajectories from modern and ancient DNA. *Genome Biology* **20**, 29 (2019).
60. Raymond, O. *et al.* The Rosa genome provides new insights into the domestication of modern roses. *Nature Genetics* **50**, 772-777 (2018).
61. Alioto, T. *et al.* Transposons played a major role in the diversification between the closely related almond and peach genomes: results from the almond genome sequence. *Plant Journal* **101**, 455-472 (2020).
62. Sánchez-Pérez, R. *et al.* Mutation of a bHLH transcription factor allowed almond domestication. *Science* **364**, 1095-1098 (2019).
63. Velasco, R. *et al.* The genome of the domesticated apple (*Malus* × *domestica* Borkh.). *Nature Genetics* **42**, 833-839 (2010).
64. Wu, J. *et al.* The genome of the pear (*Pyrus bretschneideri* Rehd.). *Genome Research* **23**, 396-408 (2013).

65. Shulaev, V. *et al.* The genome of woodland strawberry (*Fragaria vesca*). *Nature Genetics* **43**, 109-116 (2011).
66. Jaillon, O. *et al*. The grapevine genome sequence suggests ancestral hexaploidization in major angiosperm phyla. *Nature* **449**, 463-7 (2007).
67. Salse, J. Ancestors of modern plant crops. *Current Opinion in Plant Biology* **30**, 134-42 (2016).
68. Bolger, A.M., Lohse, M. & Usadel, B. Trimmomatic: a flexible trimmer for Illumina sequence data. *Bioinformatics* **30**, 2114-20 (2014).
69. Nguyen, L.-T., Schmidt, H.A., von Haeseler, A. & Minh, B.Q. IQ-TREE: A fast and effective stochastic algorithm for estimating maximum-likelihood phylogenies. *Molecular Biology and Evolution* **32**, 268-274 (2014).
70. Bandelt, H.J., Forster, P. & Röhl, A. Median-joining networks for inferring intraspecific phylogenies. *Molecular Biology and Evolution* **16**, 37-48 (1999).
71. Martin, M. Cutadapt removes adapter sequences from high-throughput sequencing reads. *EMBnet.journal* **17**, 10-12 (2011).
72. Patel, R.K. & Jain, M. NGS QC Toolkit: a toolkit for quality control of next generation sequencing data. *PLoS One* **7**, e30619 (2012).
73. Li, H. & Durbin, R. Fast and accurate short read alignment with Burrows-Wheeler transform. *Bioinformatics* **25**, 1754-60 (2009).
74. McKenna, A. *et al.* The genome analysis toolkit: a MapReduce framework for analyzing next-generation DNA sequencing data. *Genome Research* **20**, 1297-1303 (2010).
75. Chang, C.C. *et al*. Second-generation PLINK: rising to the challenge of larger and richer datasets. *GigaScience* **4**(2015).
76. Vos, P.G. *et al*. Evaluation of LD decay and various LD-decay estimators in simulated and SNP-array data of tetraploid potato. T*heoretical and Applied Genetics* **130**, 123-135 (2017).
77. Duchen, P. & Salamin, N. A cautionary note on the use of haplotype callers in Phylogenomics. B*ioRxiv*, 2020.06.10.145011 (2020).
78. Malinsky, M., Matschiner, M. & Svardal, H. Dsuite - Fast D-statistics and related admixture evidence from VCF files. *Molecular Ecology Resources* **21**, 584-595 (2021).
79. Martin, S.H., Davey, J.W. & Jiggins, C.D. Evaluating the use of ABBA-BABA statistics to locate introgressed loci. *Molecular Biology and Evolution* **32**, 244-57 (2015).
80. Raj, A., Stephens, M. & Pritchard, J.K. fastSTRUCTURE: Variational inference of population structure in large SNP data sets. *Genetics* **197**, 573-589 (2014).
81. Bourguiba, H. *et al*. Genetic structure of a worldwide germplasm collection of *Prunus armeniaca* L. reveals three major diffusion routes for varieties coming from the species' center of origin. *Frontiers in Plant Science* **11**(2020).
82. Myles, S. *et al.* Genetic structure and domestication history of the grape. *Proceedings of the National Academy of Sciences* **108**, 3530-3535 (2011).
83. Patterson, N., Price, A.L. & Reich, D. Population structure and eigenanalysis. *PLoS Genetics* **2**, e190 (2006).
84. Pfeifer, B., Wittelsbürger, U., Ramos-Onsins, S.E. & Lercher, M.J. PopGenome: an efficient Swiss army knife for population genomic analyses in R. *Molecular Biology and Evolution* **31**, 1929-1936 (2014).
85. Huson, D.H. & Bryant, D. Application of phylogenetic networks in evolutionary studies. *Molecular Biology and Evolution* **23**, 254-67 (2006).
86. Cingolani, P. *et al.* A program for annotating and predicting the effects of single nucleotide polymorphisms, *SnpEff. Fly* **6**, 80-92 (2012).

87. Excoffier, L., Dupanloup, I., Huerta-Sánchez, E., Sousa, V.C. & Foll, M. Robust demographic inference from genomic and SNP data. *PLoS Genetics* **9**, e1003905 (2013).
88. Pudlo, P. *et al*. Reliable ABC model choice via random forests. *Bioinformatics* **32**, 859-66 (2016).
89. Marin, J.-M. *et al.* Approximate Bayesian Computation using Random Forest. in *Validating and Expanding Approximate Bayesian Computation Methods (17w5025),* Banff, Canada (2017).
90. Raynal, L. *et al*. ABC random forests for Bayesian parameter inference. *Bioinformatics* **35**, 1720-1728 (2019).
91. Estoup, A., Raynal, L., Verdu, P. & Marin, J.-M. Model choice using Approximate Bayesian Computation and Random Forests: analyses based on model grouping to make inferences about the genetic history of Pygmy human populations. *Journal de la SFdS* **159**, 3 (2018).
92. Wegmann, D., Leuenberger, C., Neuenschwander, S. & Excoffier, L. ABCtoolbox: a versatile toolkit for approximate Bayesian computations. *BMC Bioinformatics* **11**, 116 (2010).
93. Excoffier, L. & Foll, M. fastsimcoal: a continuous-time coalescent simulator of genomic diversity under arbitrarily complex evolutionary scenarios. *Bioinformatics* **27**, 1332-4 (2011).
94. Zaurov, D. *et al*. Genetic Resources of Apricots (*Prunus armeniaca* L.) in Central Asia. *HortScience* **48**, 681-691 (2013).
95. Excoffier, L. & Lischer, H.E. Arlequin suite ver 3.5: a new series of programs to perform population genetics analyses under Linux and Windows. *Molecular Ecology Resources* **10**, 564-7 (2010).
96. Tellier, A. *et al*. Estimating parameters of speciation models based on refined summaries of the joint site-frequency spectrum. *PLoS One* **6**, e18155 (2011).
97. Wakeley, J. & Hey, J. Estimating ancestral population parameters. *Genetics* **145**, 847-55 (1997).
98. Breiman, L. Random Forests. *Machine Learning* **45**, 5-32 (2001).
99. Terhorst, J., Kamm, J.A. & Song, Y.S. Robust and scalable inference of population history from hundreds of unphased whole genomes. *Nature Genetics* **49**, 303-309 (2017).
100. Wang, Z. *et al*. Phylogeography study of the Siberian apricot (*Prunus sibirica L.*) in Northern China assessed by chloroplast microsatellite and DNA makers. *Frontiers in Plant Science* **8**, 1989-1989 (2017).
101. Pavlidis, P., Živkovic, D., Stamatakis, A. & Alachiotis, N. SweeD: likelihood-based detection of selective sweeps in thousands of genomes. *Molecular Biology and Evolution* **30**, 2224-2234 (2013).
102. Nei, M. Molecular Evolutionary Genetics (Columbia University Press, 1987).
103. Tajima, F. Statistical method for testing the neutral mutation hypothesis by DNA polymorphism. *Genetics* **123**, 585-95 (1989).
104. Danecek, P. *et al.* The variant call format and VCFtools. Bioinformatics **27**, 2156-8 (2011).
105. Alachiotis, N., Stamatakis, A. & Pavlidis, P. OmegaPlus: A scalable tool for rapid detection of selective sweeps in whole-genome datasets. *Bioinformatics* (Oxford, England) **28**, 2274-5 (2012).
106. Kim, Y. & Nielsen, R. Linkage disequilibrium as a signature of selective sweeps. *Genetics* **167**, 1513-1524 (2004).

107. Kitamura, Y. *et al.* Identification of QTLs controlling chilling and heat requirements for dormancy release and bud break in Japanese apricot *(Prunus mume). Tree Genetics & Genomes* **14**, 33 (2018).
108. Salazar, J.A. *et al.* Inheritance of reproductive phenology traits and related QTL identification in apricot. *Tree Genetics & Genome*s **12**, 71 (2016).
109. Bielenberg, D.G. *et al.* Genotyping by sequencing for SNP-based linkage map construction and QTL analysis of chilling requirement and bloom date in peach [*Prunus persica* (L.) Batsch]. *PLoS One* **10**, e0139406 (2015).
110. Jiang, D., Kong, N.C., Gu, X., Li, Z. & He, Y. *Arabidopsis* COMPASS-like complexes mediate histone H3 lysine-4 trimethylation to control floral transition and plant development. *PLoS Genetics* **7**, e1001330 (2011).
111. Wang, M. *et al.* PDC1, a pyruvate/α-ketoacid decarboxylase, is involved in acetaldehyde, propanal and pentanal biosynthesis in melon (*Cucumis melo* L.) fruit. *Plant Journal* **98**, 112-125 (2019).
112. García-Gómez, B.E., Salazar, J.A., Dondini, L., Martínez-Gómez, P. & Ruiz, D. Identification of QTLs linked to fruit quality traits in apricot (*Prunus armeniaca* L.) and biological validation through gene expression analysis using qPCR. *Molecular Breeding* **39**, 28 (2019).
113. Pirona, R. *et al.* Fine mapping and identification of a candidate gene for a major locus controlling maturity date in peach. *BMC Plant Biology* **13**, 166-166 (2013).
114. Calle, A. & Wünsch, A. Multiple-population QTL mapping of maturity and fruit-quality traits reveals LG4 region as a breeding target in sweet cherry (*Prunus avium* L.). *Horticulture Research* **7**, 127 (2020).
115. Carrasco-Orellana, C. *et al.* Characterization of a ripening-related transcription factor FcNAC1 from *Fragaria chiloensis* fruit. *Scientific Reports* **8**, 10524 (2018).
116. Gao, Y. *et al.* A NAC transcription factor, NOR-like1, is a new positive regulator of tomato fruit ripening. *Horticulture Research* **5**, 75 (2018).
117. Cai, L. *et al*. A fruit firmness QTL identified on linkage group 4 in sweet cherry (*Prunus avium* L.) is associated with domesticated and bred germplasm. *Scientific Reports* **9**, 5008 (2019).
118. Yeats, T.H. *et al.* Allelic diversity of NAC18.1 is a major determinant of fruit firmness and harvest date in apple (*Malus domestica*). *BioRxiv,* 708040 (2019).
119. Aharoni, A. *et al.* Gain and loss of fruit flavor compounds produced by wild and cultivated strawberry species. *Plant Cell* **16**, 3110-31 (2004).
120. Truffault, V., Riqueau, G., Garchery, C., Gautier, H. & Stevens, R.G. Is monodehydroascorbate reductase activity in leaf tissue critical for the maintenance of yield in tomato? *Journal of Plant Physiology* **222**, 1-8 (2018).
121. Decros, G. *et al.* Get the balance right: ROS homeostasis and redox signalling in fruit. *Frontiers in Plant Science* **10**(2019).
122. Hernández Mora, J.R. *et al*. Integrated QTL detection for key breeding traits in multiple peach progenies. *BMC Genomics* **18**, 404 (2017).
123. Edge, P., Bafna, V. & Bansal, V. HapCUT2: robust and accurate haplotype assembly for diverse sequencing technologies. *Genome Research* **27**, 801-812 (2017).
124. Mächler, M. & Ligges, U. scatterplot3d - An R package for visualizing multivariate data. *Journal of Statistical Software* **08**(2003).
125. Köster, J. & Rahmann, S. Snakemake--a scalable bioinformatics workflow engine. *Bioinformatics* **28**, 2520-2 (2012).
126. Wright, S. Evolution in Mendelian populations. *Genetics* **16**, 97-159 (1931).
