# Supplementary material for: Population genomics of apricots unravels domestication history and adaptive events
Source: Nat Commun. 2021 Jun 25;12:3956. doi: 10.1038/s41467-021-24283-6 (PMC8233370; doi:10.1038/s41467-021-24283-6)
Supplement: Supplementary file 27 — Description of Additional Supplementary Files [file 41467_2021_24283_MOESM27_ESM.pdf]

Description of additional supplementary files

Title: Supplementary Data 1

Description: Information about the sequenced Prunus accessions (accession ID, species, name of the accession, biological status -wild or cultivated-, their geographical origin, provider), and about their genomes (ENA ID, number of reads and percentage of mapping on the reference Marouch genomes).

(1) EIC: European-Irano-Caucasian *Prunus armeniaca*; CA, Central Asian *Prunus armeniaca*; CH, Chinese *Prunus armeniaca* (except from Xinjiang province; NW; North-Western *Prunus sibirica*; NE, North-Eastern *Prunus sibirica*. (2) Interspecific hybrid *P. armeniaca* x plum. -- other Prunus species.

Title: Supplementary Data 2

Description: RNA-seq data used for Prunus genome annotation and transcriptome analysis.

Table depicting library species, tissue, number of read pairs, read length, total base pair numbers, percentage in GC, use in this study and sample ID. (a) Data file submitted to ENA: the data uploaded to ENA are raw data.

Title: Supplementary Data 3

Description: Statistics of CH264_4 and CH320_5 sequencing data and assembly.

a. Statistics of the sequencing datasets (MinION-ONT or Illumina for *Prunus mandshurica* and *P. sibirica*: total size, number of reads, coverage, N50 and accession numbers). b. Oxford Nanopore genome sequencing assembly statistics and assembler used for assembling the Prunus genomes. c. Molecule and optical maps statistics for Prunus genomes with the two enzymes (BspQ1 and DLE). d. Hybrid scaffolding statistics of the assemblies of *Prunus mandshurica* and *P. sibirica*. e. *Prunus mandshurica* and *P. sibirica* genome assembly statistics

Title: Supplementary Data 4

Description: Statistics of Marouch #14 and cv. Stella sequencing data and assembly.

a. Statistics of the sequencing datasets (PacBio or Illumina) for *Prunus armeniaca* Marouch #14 and *P. armeniaca* cv Stella: total size, number of reads, coverage, N50 and accession numbers. b. *Prunus armeniaca* Marouch #14 and *P. armeniaca* cv Stella PacBio assembly statistics. c. *Prunus armeniaca* Marouch #14 and *P. armeniaca* cv Stella final assembly statistics. (*): pseudo chromosomes. (**): 8 pseudo chromosomes + 4 unplaced scaffolds

Title: Supplementary Data 5

Description: Comparison of existing *Prunus armeniaca* genome assemblies.

Table depicting sequencing technology, total size, number of scaffolds, scaffold N50, percentage of gaps, number of contigs, contig N50, and comparison with BUSCO (percentage of total genes, duplicated genes, fragmented genes and missing genes). Gene completion scores were calculated using BUSCO and the eudicotyledons database (*N*=2,121 genes).

Title: Supplementary Data 6

Description: Markers and genetic map information used to assemble Armeniaca genomes.

List of molecular markers (SSR and SNP) and primer sequences used in the alignment between physical and genetic maps in apricot. a. Information on genetic markers used for the 'Bergeron' map: marker name and genetic position: linkage group (LG), scaffold ID and position. b. Information on genetic markers used for the 'Bakour' map. c. Information on genetic markers used for the 'Moniqui' map. d. Information on genetic markers used for the 'Goldrich' map. e. Information on genetic markers used for the second-generation 'Goldrich' map. f. Information on genetic markers used for the 'Marlén' map. g. Information on genetic markers used for the 'Harlayne' map. h. Information on genetic markers used for the 'Lito_F2' map. i. Information on genetic markers used for the 'Lito' map. j. Information on genetic markers used for the 'BO81604311' map. k. Unpublished SSR markers developed for chromosome anchoring and mapping in the 'Goldrich' x 'Moniqui'. l. New SNP markers developed and mapping in the 'Bergeron' x 'Bakour' progenies, including information on the primer and contig sequences.

Title: Supplementary Data 7

Description: Functional annotation (proteins and repeat elements) of the four Armeniaca genome assemblies.

a. Functional annotation and protein prediction of the four Armeniaca high-quality genome assemblies. b. Analysis of repetitive elements within the four Armeniaca high-quality genome assemblies. Transposable elements include the main contributing superfamilies of Class I and Class II elements. The portion of the genome that was identified as composed of repetitive sequences but that could not be classified as transposable elements was depicted as unclassified repeats. bp is the total length of each repeat superfamily in base pair and % is the coverage percentage of repeat elements in relation to genome size.

Title: Supplementary Data 8

Description: Structural variants identified in cv. Stella, CH320_5 and CH264_4 genomes in comparison with Marouch #14 reference genome.

a. SMAP file header description. The Bionano Genomics® SMAP file contains a list of structural variants (SV) detected between query maps and reference maps. b. Structural variants identified in cv. Stella, CH320_5 and CH264_4 genomes in comparison with Marouch #14 reference genome. In red, SMAP IDs that correspond to approx. 600 Kb inversion in Marouch #14 genome in comparison with *P. armeniaca* cv. Stella, Siberian CH320_5 and *P. mandshurica* CH264_4. b. Structural variants identified in cv. Stella genome in comparison

with Marouch #14 reference genome. c. Structural variants identified in CH320_5 genome in comparison with Marouch #14 reference genome. d. Structural variants identified in CH264_4 genome in comparison with Marouch #14 reference genome.

Title: Supplementary Data 9

Description: Number and type of structural variations (SV, insertions, deletions, inversions, duplications, translocations) identified by comparing cv. Stella, CH320_5 and CH264_4 genomes to the Marouch #14 genome.

Title: Supplementary Data 10

Description: List of shared orthologs used in reconstruction of Armeniaca phylogeny.

a. Name and source of the proteome fasta files used in this study. b. List of the 298 orthologs shared among the genomes of *Arabidopsis thaliana* Col 0, *Populus trichocarpa*, *Vitis vinifera* cv. PN40024, *Fragaria vesca* v4.0, *Rosa chinensis* cv. Old Blush, *Prunus dulcis* cv.Texas, *Prunus persica* cv. Lovell, *Prunus mume*, *Prunus mandshurica* CH264_4, CH320_5, *Prunus armeniaca* cv. Stella, *Prunus armeniaca* Marouch #14. c. List of 8,848 Rosaceae shared orthologs

Title: Supplementary Data 11

Description: Synteny relationships between Rosaceae.

Illustration of the synteny between peach (*Prunus persica*), Prunus mandshurica CH264_4, *Prunus sibirica* CH320_5, *Prunus armeniaca* Marouch #14 and cv. Stella, *Prunus mume* (columns) with the conserved chromosomes and associated orthologous gene numbers (in parentheses) in lines for each of the nine protochromosomes of the ancestral Rosaceae karyotype (ARK).

Title: Supplementary Data 12

Description: Results of the ABBA-BABA test for testing introgression between Prunus populations.

Populations tested, D-statistics, Z-score, p-value, f4 ratio and number of SNPs in the different categories (BBAA, ABBA, BABA). EIC, European cultivated apricots; CA, Central Asian cultivated apricots; wild *P. armeniaca* corresponds to the two N_Par and S_Par natural populations from Central Asia (Figure 1a); CH, cultivated Chinese apricots; NW_Psib, North_Western Chinese *P. sibirica*. The North_Eastern *P. sibirica* was used as an outgroup in the test.

Title: Supplementary Data 13

Description: Identity by Descent analysis for pedigree construction and clonal relationships

a.Confirmation of parent-offspring relationships (as known from pedigres) by IBD (Identity by Descent) in PLINK V1.9. b. identity by descent (IBD) values observed by pairs of *Prunus armeniaca* accessions, for the cultivated European and Central Asian populations. c. IBD values observed by pairs of Chinese *Prunus armeniaca* accessions. d. IBD values observed by pairs of the Southern *Prunus armeniaca* (S_Par) accessions. e. IBD values observed by pairs of the Northern *Prunus armeniaca* (N_Par) accessions. f. IBD values observed by pairs of accessions in the North Western *Prunus sibirica* population. No NW_Psib accessions were removed from the dataset based on the IBD value. g. IBD values observed by pairs of in the North Eastern *Prunus sibirica* population. No NE_Psib accessions were removed from the dataset based on the IBD value. h. IBD values observed by pairs of *Prunus mume* G1 accessions. i. IBD values observed by pairs of *Prunus mume* G2 accessions. j. IBD values observed by pairs of *Prunus mume* G3 accessions. k. IBD values observed by pairs of *Prunus mume* G4 accessions. l. IBD values observed by pairs of *Prunus mume* G5 accessions. m. IBD values observed by pairs of *Prunus mume* G6 accessions. n. IBD values observed by pairs of *Prunus mume* G7 accessions. o. IBD values observed by pairs of *Prunus mume* G8 accessions.

Known parent-offsprings are depicted in Supplementary Data 1. FID1: Family ID for first individual; FID2: Family ID for second sample; RT: Relationship type inferred from .fam/.ped file; EZ: IBD sharing expected value, based on just .fam/.ped relationship; Z0: P(IBD=0); Z1, P(IBD=1); Z2: P(IBD=2); PI_HAT: Proportion IBD, i.e. P(IBD=2) + 0.5*P(IBD=1); PHE: Pairwise phenotypic code (1, 0, -1 = AA, AU, and UU pairs, respectively); DST: IBS distance, i.e. (IBS2 + 0.5*IBS1) / (IBS0 + IBS1 + IBS2); PPC: IBS binomial test; RATIO: ratio of HETHET IBS0 SNP ratio (expected value 2); IBS0: Number of IBS 0 nonmissing loci; IBS1: Number of IBS 1 nonmissing loci; IBS2: Number of IBS 2 nonmissing loci; HOMHOM: Number of IBS 0 SNP pairs used in PPC test; HETHET: Number of IBS 2 het/het SNP pairs in PPC test. In dark grey, one of the pair of closely related individuals that was subsquently eliminated from our Dataset. The blue color indicates the non-admixed Central Asian (CA) cultivated apricots. In red, threshold used in this study.

Title: Supplementary Data 14

Description: Armeniaca individuals used in the population genetic analyses

a. Armeniaca individuals used in the approximate Bayesian computation analysis (ABC); ID and cluster assignment for *K*=7. The total number of individuals is 163 (W4=23, CH=10, W1=33, W2=43, C1=25, Prunus mume=29). b. Armeniaca individuals used for selective sweep identification: ID and population

Title: Supplementary Data 15

Description: Genetic differentiation estimates (FST lower triangle, Nei's D upper triangle) among the seven Prunus populations detected at *K*=7 with fastSTRUCTURE, and genetic diversity estimates (last eight columns) for the six populations used for ABC-RF inferences.

The pairwise F_{ST} values (below the diagonal) were calculated with vcftools and Nei's *D* values (above the diagonal) with STAMPP R package. All pairwise F_{ST} were significant ($P<0.05$, Number of permutations = 1,000 calculated with 9,613 SNPs). Last eight column are genetic diversity estimates for populations used for ABC-RD inferences on the history of apricot domestication. *N*: number of individuals; S: number of polymorphic sites, π: mean standardized pairwise differences, H_O: observed heterozygosity, H_E: expected heterozygosity, F_{IS}: inbreeding coefficient. Cluster names are as follows: C1, *Prunus armeniaca* European and Irano-Caucasian cultivars subgroup; CH, Chinese landraces; W1, *Prunus armeniaca* Ferghana valley Kyrgyz sampling site; W2, wild Central Asian *P. armeniaca*; W3, Chinese North Western *Prunus sibirica*; W4, Chinese North Eastern *P. sibirica*; mume, *Prunus mume* landraces; brigantina, wild French *P. brigantina* samples. EUR: European genetic clusters; China, Chinese genetic clusters.

Title: Supplementary Data 16

Description: Scenarios and datasets for random-forest approximate Bayesian computation (ABC) simulations

a. Steps in the comparison of groups of scenarios (sets 2 to 4) or separate scenarios (set 1) to reconstruct the evolutionary history of the wild and cultivated apricots (*Prunus armeniaca*) with Random-Forest approximate Bayesian computation and 9,613 SNP markers. b. Results of the ABC-RF algorithm comparing the wild apricot evolutionary history for step 1: repartition of votes for each scenario for each replicate, and mean and standard deviations over replicates for each scenario, posterior probability and prior error rate for the best scenario, i.e., the scenario with the highest number of votes. The most likely model is highlighted in bold (10 out of 10 votes for sc2_005). c. Results of the ABC-RF algorithm comparing the wild apricot evolutionary history for step 2: repartition of votes for each scenario for each replicate, and mean and standard deviations over replicates for each scenario, posterior probability and prior error rate for the best scenario, i.e., the scenario with the highest number of votes. The most likely model is highlighted in bold (10 out of 10 votes for sc309_g2). d. Results of the ABC-RF algorithm comparing the domestication history of Chinese apricot for step 3a: repartition of votes for each scenario for each replicate, and mean and standard deviations over replicates for each scenario, posterior probability and prior error rate for the best scenario, i.e., the scenario with the highest number of votes. The most likely model is highlighted in bold (7 out of 10 votes for sc309_g2_CHN_2_GF). e. Results of the ABC-RF algorithm comparing the domestication history of European apricot for step 3b: repartition of votes for each scenario for each replicate, and mean and standard deviations over replicates for each scenario; posterior probability and prior error rate for the best scenario, i.e., the scenario with the highest number of votes. The most likely model is highlighted in bold (10 out of 10 votes for sc309_g2_C1a_3_GF). f. Results of the ABC-RF algorithm comparing the relative timing of domestication history of the Chinese and European cultivated apricots for step 4: repartition of votes for each scenario for each replicate, and mean and standard deviations over replicates for each scenario, posterior probability and prior error rate for the best scenario, i.e., the scenario with the highest number of votes. The most likely model is highlighted in bold (10 out of 10 votes for Sc309 _g2_CC_1_noW3_GF). g. Parameters estimated with approximate Bayesian

computation random-forest for the most likely scenarios of apricot domestication inferred in step 4.

Title: Supplementary Data 17

Description: Results of the ABBA-BABA test for testing introgression between Armeniaca populations: populations tested, *D*-statistics, *p* value

D-statistics significance was assessed using jackknife on 20 blocks. Population names are as follows: C1, *Prunus armeniaca* European and Irano-Caucasian cultivars subgroup; CH, Chinese landraces; W1, *Prunus armeniaca* Ferghana valley Kyrgyz sampling site; W2, wild Central Asian *P. armeniaca*; W4, Chinese North Eastern *P. sibirica*; mume, *Prunus mume* landraces; brigantina, wild French *P. brigantina* samples.

Title: Supplementary Data 18

Description: Prior distributions used for approximate Bayesian computation to reconstruct the evolutionary history of wild and cultivated apricots.

$T_{X\text{-}Y}$: divergence time between *X* and *Y* populations; $m_{X\text{-}Y}$: migration rate per generation between the *X* and *Y* populations; *Anc*: ancestral population; wild: wild populations; cult: cultivated populations; mume: *Prunus mume*; w4: North Eastern *P. sibirica* populations; w1: Southern Central Asian *P. armeniaca* natural populations; w2: Northern *P. armeniaca* natural populations.

Title: Supplementary Data 19

Description: Selective sweeps identified in European and Chinese cultivated apricots.

a. Cut-off values used for assessing the significance of selective sweeps, chosen based on demographic simulations with SMC++ indicating the interval in which 100%, 99% or 95% of the statistics values are expected to all. b. Selective sweeps identified in European cultivated apricots in comparison with Southern (W1) and Northern (W2) Central Asian *Prunus armeniaca* natural populations. c. Selective sweeps identified in Chinese cultivated apricots in comparison with Southern (W1) Central Asian *Prunus armeniaca* natural populations.

Title: Supplementary Data 20

Description: Annotation of the most relevant European and Chinese genomic regions under selection.

a. Cut-off values used for assessing the significance of selective sweeps, chosen based on demographic inferences with SMC++ indicating the interval in which 100%, 99% or 95% of the statistics values are expected to fall in. *N*=136. Cluster names are as follows: C1, *Prunus armeniaca* European and Irano-Caucasian cultivars subgroup; CH, Chinese landraces; W1, *Prunus armeniaca* Ferghana valley Kyrgyz sampling site; W2, wild Central Asian *P.*

armeniaca. *π,* nucleotide diversity; F_{ST}, Fixation or differentiation index {Wright, 1931}; D_{XY}, pairwise nucleotide substitution or absolute measure of differentiation {Nei, 1987}; Tajima *D*, neutrality index {Tajima, 1989}; CLR, composite likelihood ratio {Pavlidis, 2013}; *ω* calculates the linkage disequilibrium (LD) in 10-Kb intervals.

b: Selective sweeps detected in the C1 cluster using the top 0.5% of the composite ratio test (CLR), their position in the genome and gene annotation and predicted function. Underlined, interval that overlaps intervals identified by McDonald and Kreitman test (MKT); in red, candidate genes displayed in Supplementary Data 24. Pos_start, start position of the 10Kb interval; pos_end, end position of the 10Kb interval.

c. Genes evolving under positive selection detected in the C1 cluster using the McDonald and Kreitman test (MKT), their position in the genome, statistics value, p value, gene annotation and predicted function. In red, candidate genes displayed in Supplementary data 24. NI neutrality index quantifies the direction and degree of departure from neutrality α, fraction of adaptive nonsynonymous substitutions or proportion of substitutions driven by positive selection. Pos_start, start position of the 10Kb interval; pos_end, end position of the 10Kb interval.

d. Selective sweeps detected in the C1 cluster using the top 0.5% of the ratios of diversities in the wild and cultivated populations, of the composite ratio test (CLR), of the McDonald and Kreitman test (MKT), their position in the genome and gene annotation and predicted function. In grey, top 0.5% threshold / shared between >or= 2 tests (CLR, Tajima's *D*, *π* ratio and LD); underlined, interval that overlaps intervals identified by McDonald and Kreitman test (MKT). Pos_start, start position of the 10Kb interval; pos_end, end position of the 10Kb interval.

e. Selective sweeps detected in the C1 cluster using the top 0.5% of the differentiation values (F_{ST} or d_{XY}), their position in the genome and gene annotation and predicted function. In grey, top 0.5% threshold / shared between differentiation tests (F_{ST} and D_{XY}); underlined, interval that overlaps intervals identified by McDonald and Kreitman test (MKT). Pos_start, start position of the 10Kb interval; pos_end, end position of the 10Kb interval.

f. Selective sweeps detected in the cultivated Chinese cluster using the top 0.5% of the composite likelihood ratio test (CLR), their position in the genome and gene annotation and predicted function. Underlined, interval that overlaps intervals identified by McDonald and Kreitman test (MKT); in red, candidate genes displayed in Supplementary Data 24. Pos_start, start position of the 10Kb interval; pos_end, end position of the 10Kb interval.

g. Genes evolving under positive selection detected in the cultivated Chinese cluster using the McDonald and Kreitman test (MKT), their position in the genome, statistics value, *p* value, gene annotation and predicted function. Underlined, loci shared with other tests; in red, candidate genes displayed in Supplementary Data 24. Pos_start, start position of the 10Kb interval; pos_end, end position of the 10Kb interval.

h. Selective sweeps detected in the cultivated Chinese cluster using the top 0.5% of the ratios of diversities in the wild and cultivated populations, of the composite ratio test (CLR), of the McDonald and Kreitman test (MKT), their position in the genome and gene annotation and predicted function. In grey, top 0.5% threshold / shared between >or= 2 tests (CLR, Tajima's *D*, π ratio and LD); underlined, interval that overlaps intervals identified by McDonald and

Kreitman test (MKT); in red, candidate genes displayed in Supplementary Data 24. Pos_start, start position of the 10Kb interval; pos_end, end position of the 10Kb interval.

i. Selective sweeps detected in the cultivated Chinese cluster using the top 0.5% of the differentiation values (F_{ST} or d_{XY}), their position in the genome and gene annotation and predicted function. Underlined, interval that overlaps intervals identified by McDonald and Kreitman test (MKT); in red, candidate genes displayed in Supplementary Data 24. Pos_start, start position of the 10Kb interval; pos_end, end position of the 10Kb interval.

Title: Supplementary Data 21

Description: Number of 10Kb regions and underlying candidate genes under positive or diverging selection in European (EIC) and Chinese (CH) apricot genomes.

Positive selection has been detected using composite likelihood ratio (CLR) and McDonald and Kreitman (MK) tests, and divergent selection by F_{ST} and D_{XY} distributions

Title: Supplementary Data 22

Description: Gene ontology (GO) enrichment among genes identified in selective sweeps in the European cultivated apricot cluster.

a.Gene ontology (GO) enrichment among genes identified in selective sweeps detected by composite likelihood ratio (CLR). b. Gene ontology (GO) enrichment among genes identified in selective sweeps detected by by taking the top 0.5% of composite likelihood ratio (CLR) scores. c. Gene ontology (GO) enrichment among genes identified as evolving under positive selection by the McDonal-Kreitman test (MKT). d. Gene ontology (GO) enrichment among genes identified in selective sweeps detected by π ratios, Tajima's *D*, Omega or linkage disequilibrium in the European cultivated apricot population. e. Gene ontology (GO) enrichment among outlier genes in the F_{ST} and D_{XY} differentiation distribution contrasting the European cultivated apricot cluster and its closest wild cluster.

Title: Supplementary Data 23

Description: Gene ontology (GO) enrichment among genes identified in selective sweeps in the Chinese cultivated apricot cluster.

a.Gene ontology (GO) enrichment among genes identified in selective sweeps detected by composite likelihood ratio (CLR). b. Gene ontology (GO) enrichment among genes identified in selective sweeps detected by taking the top 0.5% of composite likelihood ratio (CLR) scores. c. Gene ontology (GO) enrichment among genes identified as evolving under positive selection by the McDonal-Kreitman test (MKT). d. Gene ontology (GO) enrichment among genes identified in selective sweeps detected by π ratios, Tajima's *D*, Omega or linkage disequilibrium in the Chinese cultivated apricot population. e. Gene ontology (GO) enrichment among outlier genes in the F_{ST} and D_{XY} differentiation distribution contrasting the Chinese cultivated apricot cluster and its closest wild cluster.

Title: Supplementary Data 24

Description: Major candidate genes under selection during apricot domestication and linked to agronomic traits of interest: candidate genes sorted by function type, gene ID, position and type of test having detected positive selection, population where positive selection has been detected, putative function and the corresponding references for the involvement in these functions.

In red, candidate gene that colocalizes with known QTL. 1, Gene Identity within the Marouch v3.0 genome assembly. In brackets, number of tandem copies. 2, Chr-chromosome and position.
